# Supplementary material for: Impact of a mass media campaign on breast cancer symptoms awareness and screening uptake in Malaysia: findings from a quasi-experimental study
Source: BMJ Open. 2020 Aug 20;10(8):e036503. doi: 10.1136/bmjopen-2019-036503 (PMC7443282; doi:10.1136/bmjopen-2019-036503)
Supplement: Supplementary data [file bmjopen-2019-036503supp001.pdf]

Appendix Table 1 Information about all campaign activities and media used during BCAC-BC

**Time period** (2018)	**Media (Language)**	**Content**	**Implementation area**
**TV**			
24th September – 21st October<br>4 weeks<br>4-6 ads/ day	Advertisement, 30 seconds on TV3 (BM), TV9 (BM), 8TV (MAN) and YouTube (BM, MAN, ENG)	Cancer survivor highlighting the signs and symptoms she experienced, promoting early detection can save lives, highlights the importance of family support	Nationwide
**Radio**			
15th – 28th October<br>2 weeks<br>5-7 ads/ day	Advertisement, 30 seconds on I) Thr Raaga (TAM) and II) Lite FM (ENG)	Cancer survivors highlighting BC signs and symptoms, BC can be cured, highlighting that early detection can save lives and to see doctor urgently if symptoms are noticed, 9 out of 10 women survive BC if found and treated early, highlighting website and helpline	I) Peninsular Malaysia<br>II) Central Malaysia (including Petaling Jaya)
**Print materials**			
24th September – 28th October<br>5 weeks	Billboards (x7) (BM)	Displayed three BC survivors from each main ethnicity as well as Amber Chia (Malaysian supermodel) and the headline 'Don't be shy to check your breasts' and the slogan '9 out of 10 women can survive BC if found and treated early', information about the website and NCSM helpline	Petaling Jaya [a]
	Street buntings (x400) (BM)	*See billboards*	Petaling Jaya [a]
	Posters (BM and ENG)	*See billboards* + signs and symptoms of BC, see a doctor urgently if symptoms are noticed, early detection can save lives, BC can be cured	Clinics (A2 size x53) in Petaling Jaya [a]<br>Tropicana Shopping Mall (A1 size x 8)
	Roll-up banners	*See billboards*	Supermarkets (x5) in Petaling Jaya [a]
	Brochures (BM, ENG, MAN and TAM)	*See billboards* + signs and symptoms of BC, Breast-self-examination guide, early detection can save lives, BC survivor story	Clinics (x53) in Petaling Jaya [a]

8th October (x2), 14th October (x2), 28th October (x1)	5x Newspaper articles (BM and ENG), i.e. MY Metro Online, Harian Metro, The Star Online, Sunday Star, Berita Harian	*Key messages same as posters*	Respective areas of coverage
**Social media**			
24th September – 28th October<br>5 weeks	32 posts, including 11 boosted posts (BM, ENG)<br>2 x posts from Amber Chia (Malaysian supermodel)	What is BC, BCAC-BC campaign launch, signs and symptoms, breast self-examination, risk factors, prevalence of BC in Malaysia, importance of male support, prevention, campaign video, cancer survivor stories (x3), call to action, screening and diagnosis, early detection saves lives, quiz	Nationwide
**Website**			
	Link: www.becanceralert.com (BM and ENG)	Information about cancer in general, signs and symptoms, risk factors and early detection as well as access to all BCAC-BC materials, cancer survivor stories	Nationwide
**Launch event**			
27th September	1 hour launch event (ENG)	Stakeholders and media were invited to officially launch the BCAC-BC	University of Malaya, Kuala Lumpur

[a] Petaling Jaya was the study area for BCAC-BC

Ads – advertisement, BCAC – Be Cancer Alert Campaign, BM – Bahasa Melayu, BC – breast cancer, ENG- English, MAN – Mandarin, NCSM – National Cancer Society Malaysia, TAM- Tamil

Appendix Table 2 Change in average prompted knowledge score

	Pre Mean (SD)	Post Mean (SD)	Change Mean (SD)	P-value *(paired sample t-test)*	P-value *(independent sample t-test, equal variances assumed)*
All participants	7.45 (3.05)	7.84 (2.86)	0.39 (3.53)	0.005	-
BC history (n=16)	8.00 (2.99)	8.06 (2.91)	0.06 (3.26)	0.940	*Comparing BC history to no history* 0.711
No BC history (n=660)	7.44 (3.06)	7.83 (2.86)	0.39 (3.54)	0.004	
BC screening history (n=175)	7.65 (3.20)	8.31 (2.85)	0.66 (3.39)	0.011	*Comparing BC screening history to no screening history* 0.251
No BC screening history (n=498)	7.38 (3.00)	7.69 (2.83)	0.31 (3.54)	0.052	
BCAC recognisers (n=441)	7.68 (2.89)	8.26 (2.46)	0.59 (3.29)	<0.001	*Comparing BCAC recognisers to non-recognisers (equal variances not assumed)* 0.0501
BCAC non-recognisers (n=211)	6.98 (3.29)	6.96 (3.32)	-0.24 (3.88)	0.929	

Appendix Table 3 Number of CBEs (n=29,000) and mammograms (n=2,051) undertaken by ethnicity and age (July 2018-January 2019)

	**CBE** *n (%)*	**Mammogram**[a] *n (%)*
**Ethnicity**		
Malay	24 532 (84.6)	1152 (56.2)
Chinese	2 434 (8.4)	551 (26.9)
Indians	1 516 (5.2)	331 (16.1)
Others	518 (1.8)	17 (0.8)
**Age group**		
20-29	16 687 (57.5)	1 (0.1)
30-39	10 722 (37.0)	14 (1.6)
40-49	1 283 (4.4)	332 (37.0)
50-59	265 (0.9)	333 (37.1)
>60	43 (0.1)	-
60-69	-	172 (19.2)
>70	-	46 (5.1)

CBE – clinical breast examination

[a] Mammogram data on age is from hospital Sg Buloh only (n=898)

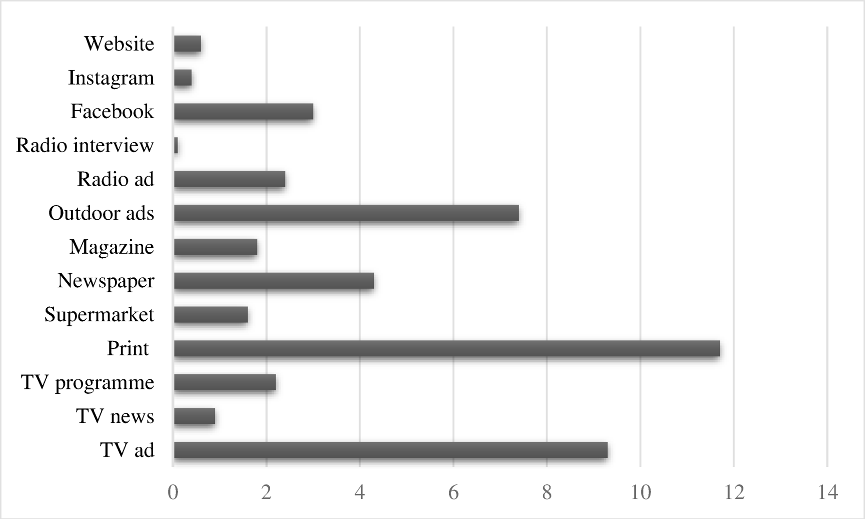

Appendix Figure 1 Advertisement channels through which participants noticed the BCAC-BC advertisements (unprompted)

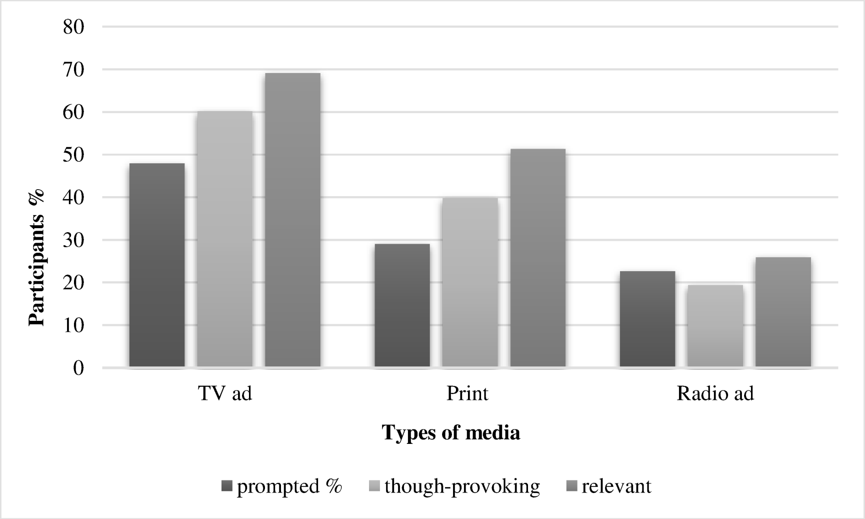

Appendix Figure 2 Advertisement channels through which participants noticed the BCAC-BC advertisements (prompted) and views on materials

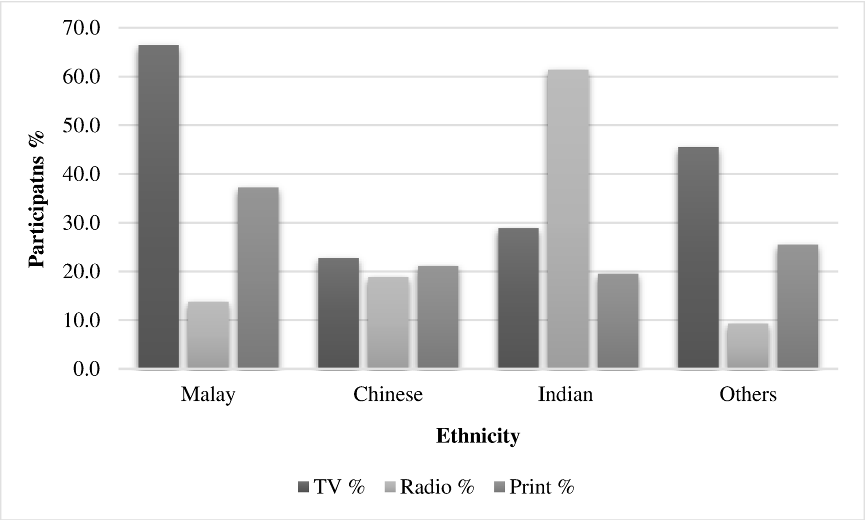

Appendix Figure 3 Difference in campaign material reach between ethnicities
