# Supplementary material for: Impact of a mass media campaign on breast cancer symptoms awareness and screening uptake in Malaysia: findings from a quasi-experimental study
Source: BMJ Open. 2020 Aug 20;10(8):e036503. doi: 10.1136/bmjopen-2019-036503 (PMC7443282; doi:10.1136/bmjopen-2019-036503)
Supplement: Author's manuscript [file bmjopen-2019-036503.draft_revisions.pdf]

BMJ Open is committed to open peer review. As part of this commitment we make the peer review history of every article we publish publicly available.

When an article is published we post the peer reviewers' comments and the authors' responses online. We also post the versions of the paper that were used during peer review. These are the versions that the peer review comments apply to.

The versions of the paper that follow are the versions that were submitted during the peer review process. They are not the versions of record or the final published versions. They should not be cited or distributed as the published version of this manuscript.

BMJ Open is an open access journal and the full, final, typeset and author-corrected version of record of the manuscript is available on our site with no access controls, subscription charges or pay-per-view fees (http://bmjopen.bmj.com).

If you have any questions on BMJ Open's open peer review process please email info.bmjopen@bmj.com

BMJ Open

The impact of mass media on breast cancer symptoms awareness and screening uptake in Malaysia

Journal:	*BMJ Open*
Manuscript ID	bmjopen-2019-036503
Article Type:	Original research
Date Submitted by the Author:	17-Dec-2019
Complete List of Authors:	Schliemann, Desiree ; Queen's University Belfast, Centre for Public Health<br>Htay , Mila; University of Malaya, Department of Social and Preventive Medicine<br>Dahlui, Maznah; University of Malaya, Department of Social and Preventive Medicine<br>Paramasivam, Darishiani; University of Malaya, Department of Social and Preventive Medicine<br>Cardwell, Chris; Queen's University Belfast, Centre for Public Health<br>Ibrahim Tamin, Nor Saleha; Ministry of Health Malaysia Disease Control Division<br>Somasundaram, Saunthari ; National Cancer Society Malaysia<br>Donnelly, Conan; National Cancer Registry<br>Su, Tin ; Monash University - Malaysia Campus, Jeffrey Cheah School of Medicine and Health Sciences<br>Donnelly, Michael; Queen's University Belfast, Centre for Public Health
Keywords:	PUBLIC HEALTH, Breast tumours < ONCOLOGY, EDUCATION & TRAINING (see Medical Education & Training)

SCHOLARONE™
Manuscripts

I, the Submitting Author has the right to grant and does grant on behalf of all authors of the Work (as defined in the below author licence), an exclusive licence and/or a non-exclusive licence for contributions from authors who are: i) UK Crown employees; ii) where BMJ has agreed a CC-BY licence shall apply, and/or iii) in accordance with the terms applicable for US Federal Government officers or employees acting as part of their official duties; on a worldwide, perpetual, irrevocable, royalty-free basis to BMJ Publishing Group Ltd ("BMJ") its licensees and where the relevant Journal is co-owned by BMJ to the co-owners of the Journal, to publish the Work in this journal and any other BMJ products and to exploit all rights, as set out in our licence.

The Submitting Author accepts and understands that any supply made under these terms is made by BMJ to the Submitting Author unless you are acting as an employee on behalf of your employer or a postgraduate student of an affiliated institution which is paying any applicable article publishing charge ("APC") for Open Access articles. Where the Submitting Author wishes to make the Work available on an Open Access basis (and intends to pay the relevant APC), the terms of reuse of such Open Access shall be governed by a Creative Commons licence – details of these licences and which Creative Commons licence will apply to this Work are set out in our licence referred to above.

Other than as permitted in any relevant BMJ Author's Self Archiving Policies, I confirm this Work has not been accepted for publication elsewhere, is not being considered for publication elsewhere and does not duplicate material already published. I confirm all authors consent to publication of this Work and authorise the granting of this licence.

For peer review only

Title: The impact of mass media on breast cancer symptoms awareness and screening uptake in Malaysia

Authors: Désirée Schliemann[1] (PhD); Mila Nu Nu Htay[2,3] (MMedSc); Maznah Dahlui[2,4] (PhD); Darishiani Paramasivam[2] (MSc), Christopher R. Cardwell[1] (PhD); Nor Saleha Binti Ibrahim Tamin[5] (MPH); Saunthari Somasundaram[6] (MBA), Conan Donnelly[7] (PhD); Tin Tin Su[2,8] (PhD); Michael Donnelly[1] (PhD)

[1] Centre for Public Health and UKCRC Centre of Excellence for Public Health, Queen's University Belfast, Belfast, UK

[2] Centre for Population Health (CePH), Department of Social and Preventive Medicine, University of Malaya, Kuala Lumpur, Malaysia

[3] Department of Community Medicine, Melaka-Manipal Medical College, Manipal Academy of Higher Education (MAHE), Melaka, Malaysia

[4] Facultas Public Health, University Airlangga, Indonesia

[5] Ministry of Health Malaysia, Kuala Lumpur, Malaysia

[6] National Cancer Society Malaysia, Kuala Lumpur, Malaysia

[7] National Cancer Registry, Cork, Ireland

[8] South East Asia Community Observatory (SEACO), Jeffrey Cheah School of Medicine and Health Sciences, Monash University Malaysia

Word count main text: 3451

Corresponding author: Désirée Schliemann / email: d.schliemann@qub.ac.uk/ phone: +44 28 9097 6350/ fax: +44 28 90235900 / ORCID ID: 0000-0002-8746-3002

For peer review only - http://bmjopen.bmj.com/site/about/guidelines.xhtml

Abstract

Objective: To evaluate the impact mass media of a campaign in terms of improving breast cancer (BC) symptoms awareness and screening uptake.

Design: Before- and after- study with comparator groups.

Setting: Selangor State, Malaysia.

Participants: Malaysian women aged ≥40 years (n=676) from randomly selected households.

Intervention: A culturally adapted mass media campaign (TV, Radio, print media and social media).

Primary and secondary outcome measures: The primary endpoint was BC symptom awareness, which was assessed with the *Breast Cancer Awareness Measure* pre- and post-campaign. Secondary outcomes included campaign reach, self-efficacy to notice BC symptoms and clinical outcomes. Clinical breast examination and mammogram screening data were collected from hospitals and clinics.

Results: Most participants recognised at least one of the campaign materials (65.2%). The odds of seeing the campaign were lowest for Chinese women (adjusted OR 0.69, 95% CI 0.42, 1.14) compared to Malays and for women aged ≥70 years (adjusted OR 0.47, 95% CI 0.23, 0.94) compared to younger women. Participants who recognised the campaign were significantly more likely to have improved awareness post-campaign compared to non-recognisers particularly for key symptoms such as 'a lump or thickening in your breast' (88.9% vs 62.1%) and 'discharge or bleeding from nipple' (79.7% vs 55.3%). Improvement in symptoms awareness scores was not associated with socio-demographic variables.

Conclusions: Implementation in Malaysia of an evidence-based mass media campaign from the UK that was culturally adapted appeared to lead to improved awareness about BC symptoms, though various modes of media communication and perhaps other health education approaches may be required to extend the reach to diverse, multi-ethnic populations and all age groups.

Key words: public health, health promotion, breast cancer, awareness, symptoms, screening, signs, clinical breast examination, mammogram, social media, media campaign.

For peer review only - http://bmjopen.bmj.com/site/about/guidelines.xhtml

Article Summary

Strengths and limitations

- The public health intervention was subjected to a systematic cultural adaptation process and drew upon best available evidence.
- This was the first study to evaluate a mass media campaign designed to improve breast cancer awareness in Malaysia.
- The study outcomes were assessed using psychometrically validated measures that were pilot-tested with the Malaysian multi-ethnic population
- The development and evaluation approach provides a blueprint for public health researchers in other Asian countries.
- The nationwide distribution of the mass media campaign meant that it was not possible to have conventional control groups though the study was able to create internal comparator groups of 'recognisers' vs 'non-recognisers'.

For peer review only

Introduction

Breast cancer (BC) comprises 17.7% of all cancers in Malaysia[1] and it is the commonest cause of cancer deaths among women in Malaysia[2]. The high cancer mortality rate is due to several factors particularly late detection. For example, between 2007–2011, 43% of women were diagnosed with BC at a late stage (stage 3 and 4)[1] compared to <20% of women in the UK[3], supporting the benefits of early detection interventions that are more commonly implemented in high income countries[4]. Late presentation is due, at least partly, to low cancer awareness. Research indicates that there is a lack of awareness among Malaysian women about common symptoms of BC[5,6], e.g. only 34% of women recognised 'a painless breast lump' as a BC symptom[6]. Other causes of delayed detection and diagnosis include denial, lack of knowledge, negative perceptions of the disease, over-reliance on traditional medicine, misperceived risk, emotional barriers and negative perceptions towards screening[7–9].

Particularly in settings where resources are limited and population-based cancer screening is unavailable, nation-wide education programmes are encouraged as a first step to improve early detection of breast cancer[10]. However, robust evaluations of mass media interventions for cancer health promotion in Asia, and particularly Malaysia, are lacking[11]. In high income countries, mass media campaigns have improved symptoms awareness[12,13] and increased the number of BC referrals[14]. Industry and non-governmental organisations (NGOs) exert mass media type efforts regularly to raise awareness about BC but these interventions tend to be short-lived and are not subjected to robust evaluation[15]. In response to this knowledge gap, our collaboration developed a culturally acceptable, evidenced-based mass media campaign for Malaysia - the *Be Cancer Alert Campaign* (BCAC)[16,17]. The primary endpoint of this study was BC symptoms awareness. Secondary outcomes included the reach of the campaign, perceived self-efficacy to detect symptoms, visits to a health care professional to discuss BC symptoms, number of BC screenings undertaken (clinical breast examination (CBE) and mammogram) and the number of BC cases diagnosed.

Methods

The protocol for the evaluation of the BCAC-BC was published previously[16] and is explained here in brief. The Research Ethics Committee, University of Malaya (ID: 2016126–4668) and the National Medical Research Register (ID: NMRR-18-1961-42562), granted ethical approval.

Study population and sampling

Malaysia comprises three main ethnicities: Malay (69%), Chinese (23%) and Indian (7%)[18]. The sample was drawn from Petaling District (Sungai Buloh and Petaling Jaya), Selangor State, with a multi-ethnic

For peer review only - http://bmjopen.bmj.com/site/about/guidelines.xhtml

population composition[16]. Trained interviewers visited randomly selected households and invited female residents to participate if they were aged ≥40 years, spoke English or Malay, were able to provide answers without support from others and provided informed consent. Participants were interviewed one-to-twelve weeks before-and-after BCAC-BC implementation.

Intervention

Implementation of the BCAC-BC mass media campaign occurred over a 5-week period (September-October 2018). Appendix Table 1 provides a description of campaign materials**.** Nationwide TV and radio presented advertisements; the study area received print materials (i.e. billboards, street bunting, posters and brochures); the National Cancer Society Malaysia (NCSM) delivered a social media campaign via their Facebook page. All materials contained a link to a bespoke BCAC website and highlighted the NCSM helpline.

Patient and public involvement

This study involved the leading advocacy organisation for cancer prevention and cancer care in Malaysia – NCSM. Representatives from NCSM, the Ministry of Health (MoH), Malaysia and the university researchers worked in partnership to address the research questions. In particular, we involved our partners, members of the public and cancer survivors in focus groups and interviews regarding the preparation of the campaign materials and their delivery and we included cancer survivors in the TV advertisements. Finally, the results of the research were disseminated in collaboration with the NCSM, MoH and cancer survivors.

Data collection

Questionnaire

Pre- and post-campaign surveys were conducted to evaluate the impact of the mass media campaign on BC awareness with questions informed by the *Breast Cancer Awareness Measure* (CAM)[19]. We assessed *Unprompted* BC symptoms knowledge by asking, 'There are many warning signs and symptoms of BC. Please name as many as you can think of'. We assessed *Prompted* knowledge by asking, 'Do you think [symptom] could be a sign for BC?' A total score for unprompted knowledge and prompted knowledge, respectively, was calculated by summing the correct answers. Socio-demographic characteristics, cancer history (of respondent) and monthly household income were assessed pre-campaign only. Post-campaign, specific questions assessed campaign reach[20]. Participants

For peer review only - http://bmjopen.bmj.com/site/about/guidelines.xhtml

were asked whether they noticed the BCAC logo and other materials. The final set of questions asked participants whether or not they found the materials relevant and acceptable; shared/discussed the campaign information and whether or not they or their family and/or friends visited a health professional as a result of seeing the BCAC-BC.

Social media monitoring

The social media activity was monitored daily by an external agency; and it was evaluated in terms of post reach (total number of unique users who saw the advertisement/post on their Facebook feed), interaction (total number of emoji reactions including like, love, smile, wow, sad and angry), amplification (number of shares per post), conversation (number of comments per post) and total engagement (total number of interactions, amplification and conversation per post).

Helpline

Trained nurses recorded (with consent) NCSM Helpline callers who said that they got the helpline number from one of the BCAC campaign materials in terms of date of call, gender of caller, reason for calling and campaign source for the number.

Health service use

Staff from local health clinics and hospitals recorded the number of CBE and mammograms undertaken between July 2018 and January 2019 as well as basic socio-demographic information.

Sample size

It was estimated that 550 participants would provide 80% power to detect, as statistically significant at a 5% level, an increase by 6% in the proportion of individuals who were aware that an unexplained lump or swelling was a symptom of breast cancer[16] using a two-sided McNemar Test.

Data analysis

The McNemar test assessed pre- and post-campaign proportional differences in knowledge/awareness. Chi-square tests examined associations between recognition of one or more BCAC materials when prompted (i.e. 'BCAC-recognisers' and 'non-recognisers') and BC awareness; and tested associations between BC history or BC screening history and BC symptoms awareness. Logistic regression

For peer review only - http://bmjopen.bmj.com/site/about/guidelines.xhtml

investigated the relationship between BCAC recognition (yes versus no) and potential explanatory variables. The final model from which adjusted estimates were calculated contained age, gender, ethnicity, marital status, education, monthly family household income, BC history and BC screening history (received BC screening – either CBE or mammogram- in the past two years). Similar models were applied to the outcome, 'knowledge improved' (yes vs no). Logistic regression analyses were repeated using robust standard errors to adjust for potential clustering within households[21] (the results were similar to the results that are presented here). Service utilisation data were charted over the relevant time-periods. All available information was included in the analysis.

Results

The BCAC-BC was implemented as planned (except that the TV advertisement was conducted for four weeks instead of five weeks (Appendix Table 1).

Study population

A total of 992 participants completed the pre-campaign survey and 68% (676/992) completed the post-campaign survey (**Table 1**). Participants who did not complete the follow-up survey could not be reached or refused to participate post-campaign. Forty-one percent of participants who completed both surveys were aged between 40 and 49 years and about 30% of women were aged between 50-59 years. Malays were the most commonly represented ethnic group (51.6 %), followed by Chinese (22.3%) and Indians (17.8%). Most women were married (86.8%); just over half completed secondary education (54.2%); and most (71%) had a monthly low household income of less than RM 4,000[22]. Sixteen women (2.4%) had a personal history of BC and 25.9% underwent a mammogram in the previous two years. Women who completed the follow-up assessment were significantly younger compared to women who completed only the pre-campaign survey. There was a higher proportion of Malay and Indian participants and fewer Chinese participants at follow-up (**Table 1**).

Campaign reach

Eighteen percent of participants reported that they previously saw the BCAC logo. Participants remembered (unprompted) seeing BCAC posters in clinics (11.7%), TV advertisements (9.3%), outdoor materials (bunting/billboards, 7.4%), newspaper articles (4.3%) and hearing BCAC radio advertisements (2.4%) (Appendix Figure 1). Furthermore, 26.9% recognised the slogan, '*Don't be shy to check your breast*'. When participants were prompted or shown BCAC materials, 65.2% recognised BCAC materials (i.e. 47.9% recognised the TV advertisements, 29% recognised print materials and

For peer review only - http://bmjopen.bmj.com/site/about/guidelines.xhtml

22.6% recognised the radio advertisement). TV advertisements were deemed thought-provoking by 60% and relevant by 69% (Appendix Figure 2). Print materials were thought-provoking for 40% and relevant for 51.3%; fewer participants described radio ads in these terms (19.4% and 25.9%). Almost 40% discussed the advertisements with friends/family and 21.7% stated that they or their family/friends went to see a doctor as a result of seeing the advertisements. TV advertisements were most commonly recognised by Malays (66.4%) followed by 'others' (45.5%). Indian participants (61.3%) recognised radio advertisements more often than other ethnic groups. Print advertisements were recognised by more Malays (37%) compared to between 19.5% and 25.5% of other ethnicities (Appendix Figure 3). Between-ethnic groups differences in campaign reach were confirmed by regression analysis (**Table 2**). The odds that participants saw one or more of the BCAC materials (when prompted) were significantly lower for Chinese and 'others' compared to Malays (adjusted OR 0.25, 95% CI 0.15; 0.40, $p<0.001$ and OR 0.34, 95% CI 0.18; 0.65, $p=0.001$). Campaign reach towards people aged ≥70 years appeared to be relatively poor (adjusted OR 0.47, 95% CI 0.23; 0.94, $p=0.032$).

Thirty-two social media 'posts' were shared through Facebook and 11/32 posts were boosted. The boosted posts reached significantly more people than 'ordinary' posts. The boosted post describing BC symptoms in Malay achieved the highest reach (reach: 202,430; total engagement: 4,498 and shares: 1,379) compared to the same post in English (reach: 18,012, total engagement: 126 and shares: 25). The second most popular post showed highlights of the campaign launch (reach: 19,071, total engagement: 816 and shares: 45) and a post on breast self-examination in English (reach: 15,673, total engagement: 322 and shares: 87). Posts that were not boosted reached between 1,495 and 486 Facebook users whereas posts that were boosted reached between 202,430 and 3,412 users.

The helpline received five calls from women (aged 22-42 years old) who called after seeing a BCAC advertisement; 4/5 women reported experiencing potential BC symptoms and one woman had had a negative biopsy result and enquired about follow-up appointments.

For peer review only

Campaign impact

Knowledge improved significantly for six BC symptoms (unprompted): 'Pain in one of your breasts or armpits' (18.9% vs 23.2%), 'discharge or bleeding from nipple' (10.1% vs 16.4%), 'nipple rash' (1.3% vs 3.6%), 'redness of your breast skin' (1.5% vs 7.2%), 'lump or thickening under armpit' (3.4% vs 7.5%) and 'changes in the shape of your breast or nipple' (1.5% vs 3.7%) significantly at follow-up (**Table 3**). Knowledge/awareness (prompted) increased significantly for three BC symptoms: 'change in the position of your nipple' (58.7% vs 67.3%), 'pain in one of your breasts or armpits' (72.5% vs 77.5%) and 'redness of your breast skin' (54.9% vs 62.4%). Overall symptom knowledge/awareness scores (prompted) improved significantly at follow up (pre-Mean 7.45 (SD: 3.05) and post-Mean 7.84 (SD: 2.86)). BCAC recognisers identified on average a higher number of BC symptoms at follow-up

For peer review only - http://bmjopen.bmj.com/site/about/guidelines.xhtml

compared to baseline (pre-Mean 7.68 (SD: 2.89) and post-Mean 8.26 (SD: 2.46)). There was some evidence that the improvement in awareness by BCAC recognisers was higher compared to non-recognisers (Mean change 0.59 (SD: 3.29) vs Mean change -0.24 (SD: 3.88)). **(**Appendix Table 2**).**

A significantly higher proportion of BCAC recognisers compared to non-recognisers who were not aware of BC symptoms at baseline, improved their knowledge (unprompted) about the symptom 'a lump or thickening in your breast' at follow-up (55.9% vs 41.0%) (**Table 3**). Furthermore, significantly more BCAC recognisers improved their awareness (prompted) about the following symptoms 'change in the position of your nipple' (62.7% vs 41.6%), 'discharge or bleeding from nipple' (79.7% vs 55.3%), 'a lump or thickening in your breast' (88.9% vs 62.1%) and 'changes in the size of your breast or nipple' (67.2% vs 46.2%) compared to non-recognisers. The proportion of women who were very/fairly confident that they would recognise a BC symptom increased at follow-up (58.9% vs 68.9%) (**Table 3**) and there was no significant difference between BCAC recognisers and non-recognisers (63.7% vs 54.4%). Pre-campaign, 96.1% of women reported that they would see their doctor within two weeks if they noticed a BC symptom and there was no change at follow-up. Regression analysis indicated that none of the socio-demographic or campaign recognition variables exerted a marked influence on BC symptoms awareness post-campaign after adjustment (**Table 4**).

From July 2018 to January 2019, 29,000 CBEs were performed (**Figure 1**) as well as 2,051 mammograms (**Figure 2**). More mammograms and CBEs were conducted in October (337 and 47,892), January (335 and 4,978) and July (331 and 4,415) compared to other months. Most CBEs were performed on Malay ethnic women (85%) (Appendix Table 3) and women aged 20-29 years (58%) and 30-39 years (37%). Mammograms were undertaken for Malays (56%), Chinese (27%) and Indians (16%).

Discussion

Research-informed BC mass media campaigns with robust evaluation are lacking in Asian countries like Malaysia[15]. The need to improve BC awareness was confirmed by the limited symptoms awareness and low confidence to notice symptoms that were identified in the pre-campaign survey results. The majority of women noticed the BCAC-BC materials, in particular, the TV advertisements (even though numerous BC awareness activities took place in October as part of BC awareness month); and they found the culturally adapted campaign materials acceptable and relevant to their life circumstances. The salience of TV advertisements as the most commonly recognised campaign material is in keeping with other robust evaluations[23,24]. Furthermore, awareness about individual BC symptoms was higher amongst BCAC-recognisers compared to non-recognisers. The high reach and improvement in symptoms awareness suggested that the campaign impacted successfully and achieved its primary objective.

For peer review only - http://bmjopen.bmj.com/site/about/guidelines.xhtml

Campaign reach varied between ethnicities though improved awareness of BC symptoms did not appear to vary across socio-demographic variables including ethnicity. The differential reach that was experienced by ethnic groups was similar to the findings from the BCAC-CRC campaign in Malaysia[25]. Different attitudes and behaviours across ethnic groups regarding help-seeking may help to explain the apparent discrepancy. For example, USA health survey participants who did not seek health information in the media and preferred to trust their health service provider tended to be older and have a lower socio-economic status[26]. Mass media as a public health programme or intervention requires careful tailoring to maximise the reach and impact of given public health messages.

Although the BCAC-BC had greater reach compared to the BCAC-CRC, the BCAC-CRC appeared to be more successful in terms of improving symptoms awareness[25], perhaps, because of the higher pre-campaign symptom knowledge/awareness (prompted and unprompted) among the BCAC-BC sample compared to BCAC-CRC participants. Awareness improved significantly at follow-up for three prompted BC symptoms whereas awareness about all prompted CRC symptoms improved. The success of the BCAC-BC was due partly to the systematic cultural adaptation process[17] that was undertaken in order to address, for example, the need to refer to breast-related issues with contextual sensitivity and to include only information about a lump. Although a lump appeared already to be a commonly known symptom, it was unacceptable culturally to present other symptoms of BC. Such restrictions did not apply to the BCAC-CRC.

BC is the best-known cancer site in most countries. It was the most common cancer reported in major online newspapers in the USA[27] and it is the most 'promoted' cancer in Malaysia[15]. Also, online information-seeking behaviour related to BC is higher during October (BC awareness month)[28,29]. Findings from the BCAC social media campaigns demonstrated higher reach and engagement for the BC social media posts compared to the CRC posts (i.e. highest reach of CRC-related post: 92,678 users vs BC-related post: 202,430 users; highest engagement with CRC-related post: 2,065 users vs BC-related post: 4,498 users). CRC campaign material about symptoms may be perceived to be less pleasant to share compared to BC symptoms. Overall, it may not be surprising that BC awareness levels are higher than other cancer sites such as CRC and that the gains from a mass media campaign directed towards BC would be smaller given this elevated baseline awareness. It might be more impactful to reserve the use of mass media campaigns for areas where there is a known low level of awareness and knowledge. The number of calls reported by NCSM as a result of both BCAC campaigns was low compared to caller volume reported in other campaign evaluations, e.g. a print and online media campaign targeting low-income African American women for 2.5 months received 97 calls[30].

The apparently high number of CBEs was encouraging and may suggest good implementation of CBE guidelines among younger women (i.e. CBE every three years) but not regarding women aged ≥40 years (5.4%) (i.e. an annual CBE)[31]. Previous research on CBE uptake in Malaysia found similar CBE

For peer review only - http://bmjopen.bmj.com/site/about/guidelines.xhtml

rates across age groups[32]. The fact that data reflects public health clinic activity may explain why most CBEs were performed on Malay women (85%) - i.e. public health clinics were utilised mainly by Malays while Chinese tended to visit private clinics for screening activity though they may avail of public health care depending on costs). Mammogram activity in this study did not appear to be affected by ethnicity and were conducted among women aged ≥40 years (98.4%) which is in line with BC screening guidelines (i.e. mammograms biennially for women aged 50-74 years)[33]. Yet, the relatively low number of mammograms may highlight gaps in secondary BC prevention and care in Malaysia. Health service use findings from the *Be Clear on Cancer* campaign in England for women aged 70 years and older suggested that though screening referrals increased, the number of cancer cases detected did not increase thereby raising questions about the value of the campaign in the face of the higher workload and low conversion rate[14]. Findings from studies in the USA present mixed results about improved screening uptake. For example, one study found that screening activity in November (following BC awareness month) increased during the 90s when BC awareness and screening were low; however, increments in screening activity of this kind lessened with time as overall screening levels reached a high threshold[34]. The USA Health Communication Survey found that TV viewing (news or entertainment) prompted health information-seeking behaviour beyond socio-demographic variables and influenced cancer screening behaviour[35]. Data covered only seven months and CBE data and private clinic data were unavailable. Mindful of service data limitations, this is the first study to present CBE and mammogram activity over a series of consecutive months in Malaysia. The highest number of mammograms and CBEs were conducted in July, October and January. October is 'breast cancer awareness month' and June and December are holiday months. Although a public awareness BC campaign may increase screening rates, due to wide-spread routine screening activity throughout the year, campaigns may lose impact regarding the detection BC cases (i.e. people at low- or medium-risk and the 'worried well' may be more likely to respond to cancer awareness campaigns)[34,36].

A recent report[37] stated that evidence about the role and effectiveness of media channels in public health campaigns is limited due, partly and for obvious reasons, to a lack of controlled studies and randomised control trials. There have been very few mass media campaigns in Asia that have been scientifically evaluated[11]. This study afforded a degree of control by creating and comparing internal groups based on whether or not they recognised the campaign. The suggestion that longer, more intensive campaigns would be more impactful[37,38] is a reasonable one. However, our evaluation demonstrated that even with a modest and short-term campaign, it is possible to improve knowledge about BC symptoms and influence help-seeking behaviour. Of course, the long-term impact of the campaign and sustainability of the improvements are unknown.

Conclusion

For peer review only - http://bmjopen.bmj.com/site/about/guidelines.xhtml

The BCAC-BC study addresses a knowledge gap and adds important insights into the impact of mass media for breast cancer health promotion as well as implementation challenges related to (South East) Asia. Overall, the findings presented here favour positively the use of mass media in breast health promotion. Future campaigns may be enhanced via increased collaboration with disciplines such as social marketers and health journalism[39]. There appears to be a need for mass media campaigns to be tailored to particular sub-populations or hard to reach subgroups, especially in the context of multi-cultural societies. Women aged ≥70 years or above for example, were less likely to observe or notice the BCAC-BC materials and to have received a mammogram (5%). Targeting older women and women from ethnic minorities may be an important consideration in efforts to improve the reach of future BC awareness activities in Malaysia[14].

Declarations

Ethics approval and consent to participate

Ethics approval for the study was granted by the Medical Research Ethics Committee, University Malaya Medical Centre (ID: 2016126–4668) and by the National Medical Research Register (NMRR-18-1961-42562). Informed consent to participate was provided by all study participants. The study was performed in accordance with the Declaration of Helsinki.

Consent for publication

Consent for publication was signed by all study participants.

Data availability

Data can be made available for inspection upon request.

Conflict of interest

The authors declare no conflict of interest.

Funding

This study is funded by UK MRC-Newton Ungku Omar Funding (MR/P013910/1). The collaborative grant application was subjected to peer-review by individual academic reviewers and the final decision

For peer review only - http://bmjopen.bmj.com/site/about/guidelines.xhtml

about funding was made by an expert panel. The funder had no role in the design of the study, collection, analysis, and interpretation of data or in writing the manuscript.

Author contributions

MDo and TTS conceptualised and planned the project and are the Co-PIs of the successful grant award from UK MRC-Newton Ungku Omar Fund. CD conducted the power calculation and provided guidance on the study design. DS, MNNH, DP, TTS and MDa planned and coordinated the study and data collection. MNNH collected data from the hospitals and clinics. DS and CC conducted the statistical analysis. DS drafted the manuscript. MDo led the editing and refinement of the manuscript. All authors contributed to, reviewed and approved the final manuscript.

Acknowledgements

We would like to acknowledge all involved in the development of the Be Cancer Alert Campaign materials and would like to acknowledge that the Be Cancer Alert Campaign materials were adapted from materials produced by the Public Health Agency, Northern Ireland for the Be Cancer Aware Campaign. We thank the Department of Statistics Malaysia for providing the randomly selected households. We would also like to acknowledge Datin Dr. Premila A/P Kanagasabai (Selayang hopsital)Dr. Yun Sii Ing (Sg Buloh hospital), Dr. Farah Nadrah Mohd Nasir (Sg Buloh Hospital), Dr Hazwan Amzar (Sungai Buloh), Dr Ainur Syahidza Bt Abd Manan (NCD Unit, PKD Petaling), Dr Nurhaidi Bt Abdullah (KK Kota Damansara), Dr. Nur Es Naini (KK Kelana Jaya), Dr. Ee Li Min (KK Taman Medan), and Dr Tengku Nor Shafadilah Bt Tg Mohd Nordin (KK Paya Jaras) for providing data on breast cancer screening and all participants for completing the surveys.

For peer review only - http://bmjopen.bmj.com/site/about/guidelines.xhtml

References

1 Azizah AM, Nor Saleha I, Noor Hashimah A, Asmah Z, Mastulu W. Malaysian National Cancer Registry Report 2007-2011. 2013 doi:MOH/P/KN/01.16(AR).

2 International Agency for Research on Cancer. GLOBOCAN. 2018http://gco.iarc.fr/today/home.

3 Cancer Research UK. Breast cancer incidence (invasive) statistics. 2017.https://www.cancerresearchuk.org/health-professional/cancer-statistics/statistics-by-cancer-type/breast-cancer/incidence-invasive#heading-Three (accessed 24 Jul2019).

4 Koo MM, Swann R, Mcphail S, Abel GA, Elliss-brookes L, Rubin GP *et al.* Presenting symptoms of cancer and stage at diagnosis: evidence from a cross-sectional, population-based study. *Lancet Oncol* 2019; **2045**: 1–7.

5 Al-Dubai SAR, Qureshi AM, Saif-Ali R, Ganasegeran K, Alwan MR, Hadi JIS. Awareness and knowledge of breast cancer and mammography among a group of malaysian women in shah alam. *Asian Pacific J Cancer Prev* 2011; **12**: 2531–2538.

6 Abdul Hadi M, Hassali MA, Shafie AA, Awaisu A. Knowledge and perception of breast cancer among women of various ethnic groups in the state of penang: A cross-sectional survey. *Med Princ Pract* 2010; **19**: 61–67.

7 Khan TM, Leong JPY, Ming LC, Khan AH. Association of knowledge and cultural perceptions of Malaysian women with delay in diagnosis and treatment of breast cancer: A systematic review. *Asian Pacific J Cancer Prev* 2015; **16**: 5349–5357.

8 Yu FQ, Murugiah MK, Khan AH, Mehmood T. Meta-synthesis exploring barriers to health seeking behaviour among Malaysian breast cancer patients. *Asian Pac J Cancer Prev* 2015; **16**: 145–152.

9 Mahmud A, Aljunid SM. The uptake of mammogram screening in Malaysia and its associated factors: A systematic review. *Med J Malaysia* 2018; **73**: 202–211.

10 Talib Z, Amersi F, Harit A, Saleh M. Promoting Breast Cancer Awareness and Clinical Breast Examination in the LMIC: Experiences from Tajikistan, Pakistan and Kenya. *Curr Breast Cancer Rep* 2019; : 152–157.

11 Schliemann D, Su TT, Paramasivam D, Treanor C, Dahlui M, Loh SY *et al.* Effectiveness of Mass and Small Media Campaigns to Improve Cancer Awareness and Screening Rates in Asia: A Systematic Review. *J Glob Oncol* 2019.

12 Public Health Agency. Evaluation Report Breast Campaign. 2016.

For peer review only - http://bmjopen.bmj.com/site/about/guidelines.xhtml

13 Croager EJ, Gray V, Pratt IS, Slevin T, Pettigrew S, D'arcy Holman C *et al.* Find Cancer Early: Evaluation of a community education campaign to increase awareness of cancer signs and symptoms in people in regional Western Australians. *Front Public Heal* 2018; **6**: 4–9.

14 Mazari FAK, Holt S, Azmy IA. The impact of "Be Clear on Cancer" campaign on breast care services provided by a specialist oncoplastic unit – A retrospective case control study. *Int J Surg* 2017; **47**: 54–60.

15 Loh SY, Somasundaram S, Su TT. A Review of Cancer Awareness in Malaysia – What ' s Next ? *Open Access J Cancer Oncol* 2017; **1**: 1–6.

16 Schliemann D, Donnelly M, Dahlui M, Loh SY, Tamin Ibrahim NSB, Somasundaram S *et al.* The 'Be Cancer Alert Campaign': protocol to evaluate a mass media campaign to raise awareness about breast and colorectal cancer in Malaysia. *BMC Cancer* 2018; **18**: 881.

17 Schliemann D, Su TT, Paramasivam D, Ibrahim Tamin NSB, Dahlui M, Loh SY *et al.* The systematic cultural adaptation of a UK public health cancer awareness raising programme for Malaysia: The Be Cancer Alert Campaign. *Transl Behav Med* 2019.

18 Department of Statistics Malaysia. Current Population Estimates, Malaysia, 2017-2018. 2018.https://www.dosm.gov.my/v1/index.php?r=column/cthemeByCat&cat=155&bul_id=c1pqTnFjb29HSnNYNUpiTmNWZHArdz09&menu_id=L0pheU43NWJwRWVSZklWdzQ4TlhUUT09 (accessed 28 Jan2019).

19 Cancer Research UK KCL& UCL. Breast Module of the Cancer Awareness Measure. 2010http://www.cancerresearchuk.org/sites/default/files/health_professional_breast_cam_toolkit_09.02.11.pdf.

20 Public Health Agency. Evaluation Report Primer and Lung Campaigns. 2015.

21 Kirkwood B, Sterne J. *Essential Medical Statistics (2nd Edition)*. Blackwell Publishing, 2003.

22 Ng A, Firouz AMM, Khalidi JR, Muhtar MA, Tumin SA, Man TK *et al.* The state of households 2018: different realities. 2018.

23 Public Health England. Evaluation of the Be Clear on Cancer lung cancer awareness campaign regional pilot, East and West Midlands. London, 2015.

24 PHA. Evaluation Report Breast Campaign. 2016.

25 Schliemann D, Paramasivam D, Dahlui M, Cardwell CR, Somasundaram S, Ibrahim Tamin NSB *et al.* Change in public awareness of colorectal cancer symptoms following the Be Cancer Alert Campaign in the multiethnic population of Malaysia. *(submitted)* 2019.

For peer review only - http://bmjopen.bmj.com/site/about/guidelines.xhtml

26 Ramanadhan S, Viswanath K. Health and the information nonseeker: a profile. *Health Commun* 2006; **20**: 131–139.

27 Hurley RJ, Riles JM, Sangalang A. Online Cancer News: Trends Regarding Article Types, Specific Cancers, and the Cancer Continuum. *Health Commun* 2014; **29**: 41–50.

28 Glynn RW, Kelly JC, Coffey N, Sweeney KJ, Kerin MJ. The effect of breast cancer awareness month on internet search activity - a comparison with awareness campaigns for lung and prostate cancer. *BMC Cancer* 2011; **11**: 442.

29 Phillips CA, Leahy AB, Li Y, Shapira MM, Bailey L. Relationship Between State-Level Google Online Search Volume and Cancer Incidence in the United States: Retrospective Study. *J Med Internet Res* 2018; **20**.

30 Wallington SF, Oppong B, Iddirisu M, Adams-Campbell LL. Developing a Mass Media Campaign to Promote Mammography Awareness in African American Women in the Nation's Capital. *J Community Health* 2018; **43**: 633–638.

31 Ministry of Health Malaysia. Garis Panduan Program Pengesanan Awal Kanser Payudara Kebangsaan. Malaysia: Bahagian Pembangunan Kesihatan Keluarga, Kementerian Kesihatan Malaysia; 2011. Report No.: MOH/K/ASA/44.11(GU). Putrajaya, 2011.

32 Farid NDN, Aziz NA, Al-Sadat N, Jamaludin M, Dahlui M. Clinical breast examination as the recommended breast cancer screening modality in a rural community in Malaysia; what are the factors that could enhance its uptake? *PLoS One* 2014; **9**: 1–6.

33 Ministry of Health Malaysia. Clinical Practice Guidelines Management of Breast Cancer Malaysia 2010. Putrajaya, 2010http://www.moh.gov.my/penerbitan/CPG2017/6915.pdf.

34 Jacobsen GD, Jacobsen KH. Health awareness campaigns and diagnosis rates: Evidence from National Breast Cancer Awareness Month. *J Health Econ* 2011; **30**: 55–61.

35 Li W, Watts J, Tan N. From Screen to Screening: Entertainment and News Television Media Effects on Cancer Screening Behaviors. *J Health Commun* 2019; **24**: 385–394.

36 Karabay O, Hasbahceci M, Kadioglu H. Impact of breast cancer awareness month on detection of breast cancer in a private hospital. *J Int Med Res* 2018; **46**: 619–625.

37 Stead M, Angus K, Langley T, Katikireddi SV, Hinds K, Hilton S *et al.* Mass media to communicate public health messages in six health topic areas: a systematic review and other reviews of the evidence. *Public Heal Res* 2019; **7**: 1–206.

38 Wakefield MA, Loken B, Hornik RC. Use of mass media campaigns to change health behaviour. *Lancet* 2010; **376**: 1261–1271.

For peer review only - http://bmjopen.bmj.com/site/about/guidelines.xhtml

39 Hannon P, Lloyd GP, Viswanath K, Smith T, Basen-Engquist K, Vernon SW *et al.* Mass media and marketing communication promoting primary and secondary cancer prevention. *J Health Commun* 2009; **14**: 30–37.

For peer review only

For peer review only - http://bmjopen.bmj.com/site/about/guidelines.xhtml

Table legends

Table 1 Socio-demographic characteristics of respondents regarding the breast cancer campaign (n=676)

Table 2 The relationship between the socio-demographic characteristics of respondents and their recognition of any aspect of the BCAC-BC campaign [a]

Table 3 Be Cancer Alert Campaign – Breast Cancer awareness pre- and post-campaign (n=676)

Table 4 Improvement in overall prompted symptom awareness by socio-demographic characteristics and recognition of BCAC-BC advertisements (binary logistic regression)

Figure legends

Figure 1 CBEs undertaken in clinics between July 2018 and January 2019

Figure 2 Mammograms undertaken in hospitals between July 2018 and January 2019

Appendix

Appendix Table 1 Information about all campaign activities and media used during BCAC-BC

Appendix Table 2 Change in average prompted knowledge score

Appendix Table 3 Number of CBEs (n=29,000) and mammograms (n=2 051) undertaken by ethnicity and age (July 2018-January 2019)

Appendix Figure 1 Advertisement channels through which participants noticed the BCAC-BC advertisements (unprompted)

Appendix Figure 2 Advertisement channels through which participants noticed the BCAC-BC advertisements (prompted) and thoughts on materials

Appendix Figure 3 Difference in campaign material reach between ethnicities

For peer review only - http://bmjopen.bmj.com/site/about/guidelines.xhtml

Tables

Table 1 Socio-demographic characteristics of respondents pre- and post-campaign

	Pre only<br>*n (%)*<br>*n=316*	Pre and post<br>*n (%)*<br>*n=676*
**Age**		
40-49 years	104 (32.9)	274 (41.0)
50-59 years	102 (32.3)	199 (29.8)
60-69 years	67 (21.2)	137 (20.5)
≥70 years	41 (13.0)	58 (8.7)
**Ethnicity**		
Malay	150 (47.5)	349 (51.6)
Chinese	102 (32.3)	151 (22.3)
Indian	39 (12.3)	120 (17.8)
Others	25 (7.9)	56 (8.3)
**Religion**		
Islam	169 (53.5)	406 (60.1)
Christianity	26 (8.2)	50 (7.4)
Buddhism	81 (25.6)	109 (16.1)
Hinduism	31 (9.8)	100 (14.8)
Others	3 (0.9)	6 (0.8)
**Marital status**		
Married	265 (83.9)	587 (86.8)
Single	51 (16.1)	89 (13.2)
**Education**		
No formal education	45 (14.2)	88 (13.0)
Primary	52 (16.5)	97 (14.4)
Secondary	158 (50.0)	366 (54.2)
Tertiary	59 (18.7)	124 (18.4)
**Family income**		
<RM 4,000	189 (59.8)	457 (71.2)
RM 4,000-10,000	72 (22.8)	140 (21.8)
>RM 10,000	23 (7.3)	45 (7.0)
**BC history** (self only)		
No	307 (97.2)	660 (97.6)
Yes	9 (2.8)	16 (2.4)
**BC screening history** [d]		
No	222 (70.3)	498 (75.5)
Yes	94 (29.7)	175 (25.9)

[a] Participants who are widowed, divorced and who never married

[b] No formal education – includes never schooled/ never completed primary school; primary education – includes completed primary school; secondary education – includes completed form 3/ completed form 5/ certificate/ A-level/ STPM/ HSC; tertiary education – includes diploma/ bachelor degree/ post-graduate degree

[c] Income of all household member combined

[d] BC screening history refers to mammogram in the past 2 years

Missing information: Age (n=3), Religion (n=5), Monthly family income (n=34), BC screening history (n=3)

For peer review only

Table 2 The relationship between the socio-demographic characteristics of respondents and their recognition of any aspect of the BCAC-BC campaign[a]

	n (%)	OR (95% CI) (unadjusted)	*P*	OR (95% CI) (adjusted)[b]	*P*
**Age**					
40-49 years	198/274 (72.3)	*Reference*		*Reference*	
50-59 years	132/199 (66.3)	0.72 (0.48, 1.09)	0.120	0.74 (0.47, 1.16)	0.187
60-69 years	78/137 (56.9)	0.48 (0.31, 0.75)	0.001	0.65 (0.39, 1.10)	0.107
≥70 years	28/58 (48.3)	0.33 (0.18, 0.60)	<0.001	0.47 (0.23, 0.94)	0.032
**Ethnicity**					
Malay	265/349 (75.9)	*Reference*		*Reference*	
Chinese	68/151 (45.0)	0.25 (0.16, 0.37)	<0.001	0.25 (0.15, 0.40)	<0.001
Indian	81/120 (67.5)	0.62 (0.39, 0.99)	0.046	0.69 (0.42, 1.14)	0.145
Others	27/56 (48.2)	0.29 (0.16, 0.52)	<0.001	0.34 (0.18, 0.65)	0.001
**Marital Status**					
Married	380/566 (67.1)	*Reference*		*Reference*	
Single	61/86 (70.9)	1.19 (0.73, 1.96)	0.484	1.26 (0.72, 2.20)	0.422
**Education**					
No formal education	46/88 (52.3)	*Reference*		*Reference*	
Primary	57/97 (58.8)	1.34 (0.74, 2.44)	0.333	1.33 (0.68, 2.57)	0.404
Secondary	255/366 (69.7)	2.14 (1.32, 3.48)	0.002	1.65 (0.93, 2.91)	0.085
Tertiary	82/124 (66.1)	1.99 (1.11, 3.55)	0.021	1.98 (0.93, 4.18)	0.075
**Monthly family income**					
<RM 4,000	311/443 (70.2)	*Reference*		*Reference*	
RM 4,000 -10,000	82/135 (60.7)	0.66 (0.44, 0.98)	0.040	0.73 (0.46, 1.18)	0.202
RM > 10,000	27/43 (62.8)	0.72 (0.37, 1.37)	0.315	0.88 (0.41, 1.86)	0.728
**BC history**					
No	430/660 (65.2)	*Reference*		*Reference*	
Yes	11/16 (68.8)	1.05 (0.36, 3.07)	0.923	1.21 (0.37, 3.98)	0.754
**BC screening history** [c]					
No	337/510 (66.1)	*Reference*		*Reference*	
Yes	103/163 (63.2)	0.93 (0.63, 1.36)	0.705	1.03 (0.67, 1.60)	0.893

n- number of participants 'reached' or who reported that they saw (one or more parts of) the campaign divided by the total number of survey participants

[a] This includes participants who reported that they have been exposed to either the BCAC-BC TV, radio and/or print materials when prompted with the advertisement at follow-up

[b] adjusted for age, ethnicity, marital status, education, monthly family income, BC history and BC screening history

[c] If people replied 'yes' to BC history and BC screening history, they were coded as 'yes' for BC history and 'no' for BC screening history in this model

For peer review only

Table 3 Be Cancer Alert Campaign – Breast Cancer awareness pre- and post-campaign (n=676)

Survey question	**Pre** n (%)	**Post** n (%)	***P*** *(McNemar)*	**Knowledge improvement in BCAC recognisers**[a] n (%)	**Knowledge improvement in BCAC non-recognisers**[b] n (%)	***P*** *(chi-square)*
**Signs and symptoms (unprompted)**						
Change in the position of your nipple	9 (1.3)	7 (1.0)	0.804	7/435 (1.6)	0/208 (0.0)	0.152
Pulling in your nipple	7 (1.0)	16 (2.4)	0.064	11/438 (2.5)	3/207 (1.4)	0.565
Pain in one of your breasts or armpits	128 (18.9)	157 (23.2)	0.041	74/351 (21.1)	27/179 (15.1)	0.122
Puckering or dimpling of your breast skin	4 (0.6)	7 (1.0)	0.549	5/439 (1.1)	2/210 (1.0)	0.999
Discharge or bleeding from your nipple	68 (10.1)	111 (16.4)	<0.001	55/401 (13.7)	19/188 (10.1)	0.272
A lump or thickening in your breast	440 (65.1)	437 (64.6)	0.897	80/143 (55.9)	34/83 (41.0)	0.042
Nipple rash	9 (1.3)	24 (3.6)	0.012	19/437 (4.3)	4/207 (1.9)	0.188
Redness of your breast skin	10 (1.5)	49 (7.2)	<0.001	35/436 (8.0)	11/207 (5.3)	0.279
Lump or thickening under your armpit	23 (3.4)	51 (7.5)	0.001	35/426 (8.2)	10/205 (4.9)	0.174
Changes in the size of your breast or nipple	12 (1.8)	18 (2.7)	0.327	13/435 (3.0)	2/206 (9.7)	0.194
Changes in the shape of your breast or nipple	10 (1.5)	25 (3.7)	0.015	16/434 (3.7)	8/208 (3.8)	1.000
**Signs and symptoms (prompted)**						
Change in the position of your nipple	397 (58.7)	455 (67.3)	<0.001	106/169 (62.7)	42/101 (41.6)	0.001
Pulling in your nipple	391 (57.8)	397 (58.7)	0.745	85/182 (46.7)	32/92 (34.8)	0.079
Pain in one of your breasts or armpits	490 (72.5)	524 (77.5)	0.029	81/110 (44.5)	45/69 (65.2)	0.302
Puckering or dimpling of your breast skin	379 (56.1)	380 (56.2)	0.999	100/187 (53.5)	43/97 (44.3)	0.181
Discharge or bleeding from your nipple	557 (82.4)	560 (82.8)	0.814	55/69 (79.7)	26/47 (55.3)	0.009
A lump or thickening in your breast	598 (88.5)	612 (90.5)	0.211	40/45 (88.9)	18/29 (62.1)	0.014
Nipple rash	345 (51.0)	367 (54.3)	0.227	111/213 (52.1)	46/109 (42.2)	0.117
Redness of your breast skin	371 (54.9)	422 (62.4)	0.003	112/193(58.0)	47/103 (45.6)	0.055
Lump or thickening under your armpit	549 (81.2)	566 (83.7)	0.213	54/68 (79.4)	34/54 (63.0)	0.070
Changes in the size of your breast or nipple	476 (70.4)	497 (73.5)	0.173	78/116 (67.2)	36/78 (46.2)	0.005
Changes in the shape of your breast or nipple	486 (71.9)	511 (75.6)	0.105	80/114 (70.2)	38/69 (55.1)	0.056
				**Improved attitudes in recognisers**[a]	**Improved attitudes in non-recogniser**[b]	
How confident are you that you would notice a BC symptom? *(those very confident/ fairly confident)*	391 (58.9)	439 (68.9)	<0.001	100/157 (63.7)	56/103 (54.4)	0.170
How soon would you go and see a doctor if you noticed a BC sign/symptom? *(those within 2 weeks)*	614 (96.1)	637 (97.1)	0.337	19/20 (95.0)	13/14 (92.9)	0.999

Missing information: confidence BL n=12; confidence FU n = 39; delayed help seeking BL n=20; delayed help seeking FU n=37
[a] This includes participants who reported that they have been exposed to either the BCAC-BC TV, radio and/or print materials when prompted at follow-up
[b] This includes all participants who reported that they have not seen any of the BCAC-BC TV, radio and/ or print materials when prompted at follow-up

For peer review only - http://bmjopen.bmj.com/site/about/guidelines.xhtml

Table 4 Improvement in overall prompted symptom awareness by socio-demographic characteristics and recognition of BCAC-BC advertisements (binary logistic regression)

	n (%)	OR (95% CI) (unadjusted)	*P*	OR (95% CI) (adjusted)[a]	*P*
**Age**					
40-49 years	122/274 (44.5)	*Reference*		*Reference*	
50-59 years	101/199 (50.8)	1.28 (0.89, 1.85)	0.181	1.36 (0.91, 2.04)	0.130
60-69 years	60/137 (43.8)	0.97 (0.64, 1.47)	0.888	0.97 (0.60, 1.56)	0.890
≥70 years	26/58 (44.8)	1.01 (0.57, 1.79)	0.966	0.99 (0.51, 1.93)	0.982
**Ethnicity**					
Malay	162/349 (46.4)	*Reference*		*Reference*	
Chinese	63/151 (41.7)	0.83 (0.56, 1.22)	0.333	0.82 (0.51, 1.33)	0.420
Indian	58/120 (48.3)	1.08 (0.71, 1.64)	0.717	1.26 (0.75, 2.12)	0.392
Others	30/56 (53.6)	1.33 (0.76, 2.35)	0.321	1.27 (0.67, 2.41)	0.461
**Marital Status**					
Married	270/587 (46.0)	*Reference*		*Reference*	
Single	43/89 (48.3)	1.10 (0.70, 1.72)	0.683	1.17 (0.72, 1.90)	0.532
**Education**					
No formal education	39/88 (44.3)	*Reference*		*Reference*	
Primary	56/97 (57.7)	1.72 (0.96, 3.07)	0.069	1.50 (0.80, 2.81)	0.210
Secondary	156/366 (42.6)	0.93 (0.58, 1.49)	0.773	0.93 (0.54, 1.59)	0.789
Tertiary	62/124 (50.0)	1.26 (0.73, 2.17)	0.415	1.53 (0.77, 3.04)	0.227
**Monthly family income**					
<RM 4000	210/457 (46.0)	*Reference*		*Reference*	
RM 4000-10,000	67/140 (47.9)	1.08 (0.74, 1.58)	0.693	1.15 (0.74, 1.79)	0.528
RM >10,000	19/45 (42.2)	0.86 (0.46, 1.60)	0.632	0.84 (0.42, 1.71)	0.636
**BC history**					
No	307/660 (46.5)	*Reference*		*Reference*	
Yes	6/16 (37.5)	0.69 (0.25, 1.92)	0.477	0.54 (0.18, 1.65)	0.281
**BC screening history**					
No	237/510 (46.5)	*Reference*		*Reference*	
Yes	75/163 (46.0)	0.98 (0.69, 1.40)	0.919	0.83 (0.56, 1.24)	0.358
**TV ad recognition**					
No	155/347 (44.7)	*Reference*		*Reference*	
Yes	154/324 (47.5)	1.12 (0.83, 1.52)	0.457	1.22 (0.85, 1.77)	0.281
**Radio ad recognition**					
No	240/511 (47.0)	*Reference*		*Reference*	
Yes	68/153 (44.4)	0.90 (0.63, 1.30)	0.583	0.79 (0.51, 1.22)	0.289
**Print ad recognition**					
No	221/468 (47.2)	*Reference*		*Reference*	
Yes	88/196 (44.9)	0.91 (0.65, 1.27)	0.584	0.96 (0.66, 1.40)	0.847

[a]Adjusted for age, ethnicity, marital status, education, monthly family income, BC history, BC screening history, TV ad recognition, Radio ad recognition, Print at recognition

For peer review only

Figures

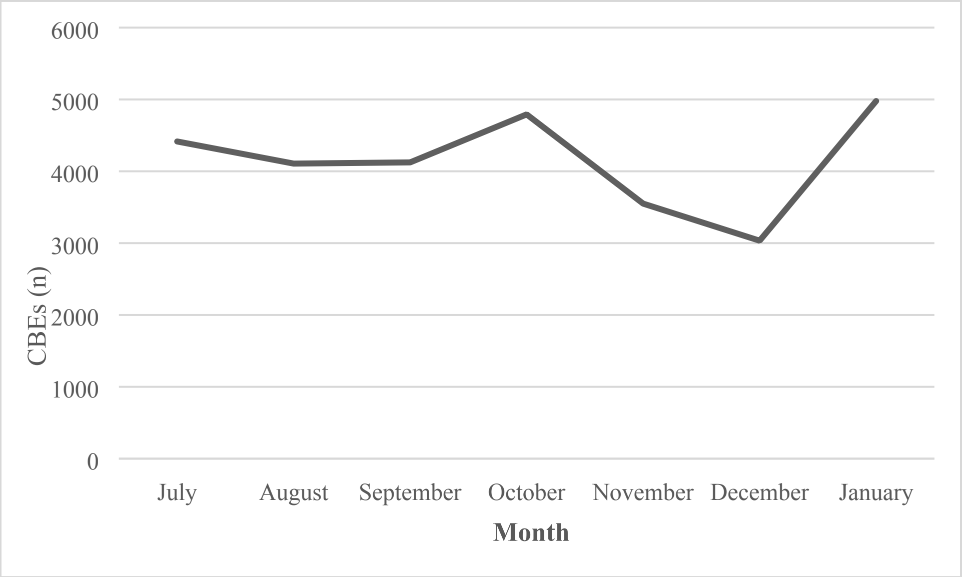

Figure 1 Clinical Breast Examinations (CBEs) undertaken in four public clinics (Petaling district) between July 2018 and January 2019

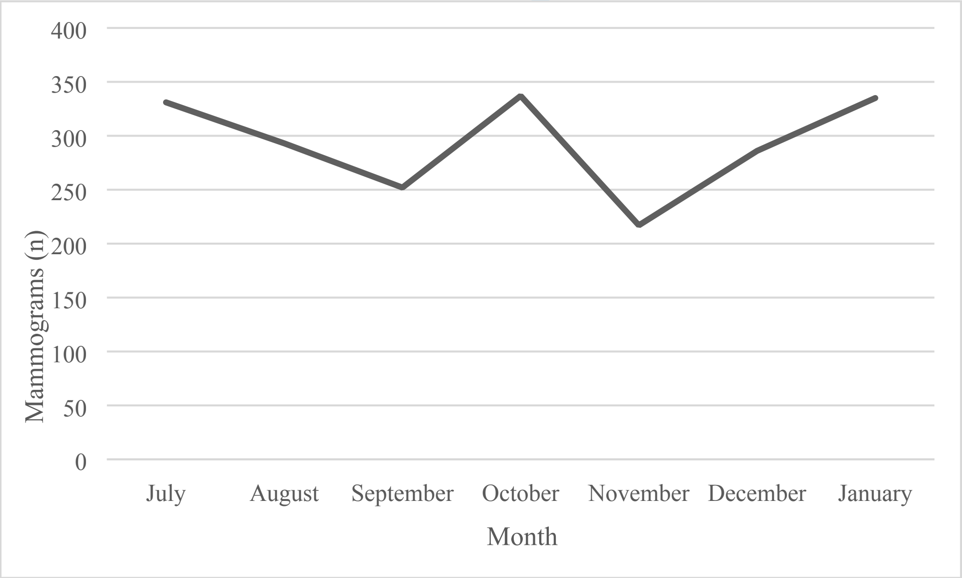

Figure 2 Mammograms undertaken in two public hospitals (Petaling district) between July 2018 and January 2019

For peer review only - http://bmjopen.bmj.com/site/about/guidelines.xhtml

Appendix Table 1 Information about all campaign activities and media used during BCAC-BC

**Time period** (2018)	**Media (Language)**	**Content**	**Implementation area**
**TV**			
2nd April – 6th May<br>4 weeks<br>4-6 ads/ day	Advertisement, 30 seconds on TV3 (BM), TV9 (BM), 8TV (MAN) and YouTube (BM, MAN, ENG)	Cancer survivor highlighting the signs and symptoms she experienced, promoting early detection can save lives, highlights the importance of family support	Nationwide
**Radio**			
9th – 22nd April<br>2 weeks<br>5-7 ads/ day	Advertisement, 30 seconds on I) Thr Raaga (TAM) and II) Lite FM (ENG)	Cancer survivors highlighting BC signs and symptoms, BC can be cured, highlighting that early detection can save lives and to see doctor urgently if symptoms are noticed, 9 out of 10 women survive BC if found and treated early, highlighting website and helpline	I) Peninsular Malaysia<br>II) Central Malaysia (including Petaling Jaya)
**Print materials**			
2nd April – 6th May<br>5 weeks	Billboards (x7) (BM)	Displayed three BC survivors from each main ethnicity as well as Amber Chia (Malaysian supermodel) and the headline 'Don't be shy to check your breasts' and the slogan '9 out of 10 women can survive BC if found and treated early', information about the website and NCSM helpline	Petaling Jaya [a]
	Street buntings (x400) (BM)	*See billboards*	Petaling Jaya [a]
	Posters (BM and ENG)	*See billboards* + signs and symptoms of BC, see a doctor urgently if symptoms are noticed, early detection can save lives, BC can be cured	Clinics (A2 size x53) in Petaling Jaya [a]<br>Tropicana Shopping Mall (A1 size x 8)
	Roll-up banners	*See billboards*	Supermarkets (x5) in Petaling Jaya [a]
	Brochures (BM, ENG, MAN and TAM)	*See billboards* + signs and symptoms of BC, Breast-self-examination guide, early detection can save lives, BC survivor story	Clinics (x53) in Petaling Jaya [a]

8th October (x2), 14th October (x2), 28th October (x1)	5x Newspaper articles (BM and ENG), i.e. MY Metro Online, Harian Metro, The Star Online, Sunday Star, Berita Harian	*Key messages same as posters*	Respective areas of coverage
**Social media**			
	32 posts, including 11 boosted posts (BM, ENG) 2 x posts from Amber Chia (Malaysian supermodel)	What is BC, BCAC-BC campaign launch, signs and symptoms, breast self-examination, risk factors, prevalence of BC in Malaysia, importance of male support, prevention, campaign video, cancer survivor stories (x3), call to action, screening and diagnosis, early detection saves lives, quiz	Nationwide
**Website**			
	Link: www.becanceralert.com (BM and ENG)	Information about cancer in general, signs and symptoms, risk factors and early detection as well as access to all BCAC-BC materials, cancer survivor stories	Nationwide
**Launch event**			
27th September	1 hour launch event (ENG)	Stakeholders and media were invited to officially launch the BCAC-BC	University of Malaya, Kuala Lumpur

[a] Petaling Jaya was the study area for BCAC-BC

Ads – advertisement, BCAC – Be Cancer Alert Campaign, BM – Bahasa Melayu, BC – breast cancer, ENG- English, MAN – Mandarin, NCSM – National Cancer Society Malaysia, TAM- Tamil

For peer review only

Appendix Table 2 Change in average prompted knowledge score

	Pre Mean (SD)	Post Mean (SD)	Change Mean (SD)	P-value *(paired sample t-test)*	P-value *(independent sample t-test, equal variances assumed)*
All participants	7.45 (3.05)	7.84 (2.86)	0.39 (3.53)	0.005	-
BC history (n=16)	8.00 (2.99)	8.06 (2.91)	0.06 (3.26)	0.940	*Comparing BC history to no history* 0.711
No BC history (n=660)	7.44 (3.06)	7.83 (2.86)	0.39 (3.54)	0.004	
BC screening history (n=175)	7.65 (3.20)	8.31 (2.85)	0.66 (3.39)	0.011	*Comparing BC screening history to no screening history* 0.251
No BC screening history (n=498)	7.38 (3.00)	7.69 (2.83)	0.31 (3.54)	0.052	
BCAC recognisers (n=441)	7.68 (2.89)	8.26 (2.46)	0.59 (3.29)	<0.001	*Comparing BCAC recognisers to non-recognisers (equal variances not assumed)* 0.0501
BCAC non-recognisers (n=211)	6.98 (3.29)	6.96 (3.32)	-0.24 (3.88)	0.929	

Appendix Table 3 Number of CBEs (n=29,000) and mammograms (n=2 051) undertaken by ethnicity and age (July 2018-January 2019)

	**CBE** *n* (%)	**Mammogram**[a] *n* (%)
**Ethnicity**		
Malay	24 532 (84.6)	1152 (56.2)
Chinese	2 434 (8.4)	551 (26.9)
Indians	1 516 (5.2)	331 (16.1)
Others	518 (1.8)	17 (0.8)
**Age group**		
20-29	16 687 (57.5)	1 (0.1)
30-39	10 722 (37.0)	14 (1.6)
40-49	1 283 (4.4)	332 (37.0)
50-59	265 (0.9)	333 (37.1)
>60	43 (0.1)	-
60-69	-	172 (19.2)
>70	-	46 (5.1)

CBE – clinical breast examination

[a] Mammogram data on age is from hospital Sg Buloh only (n=898)

For peer review only

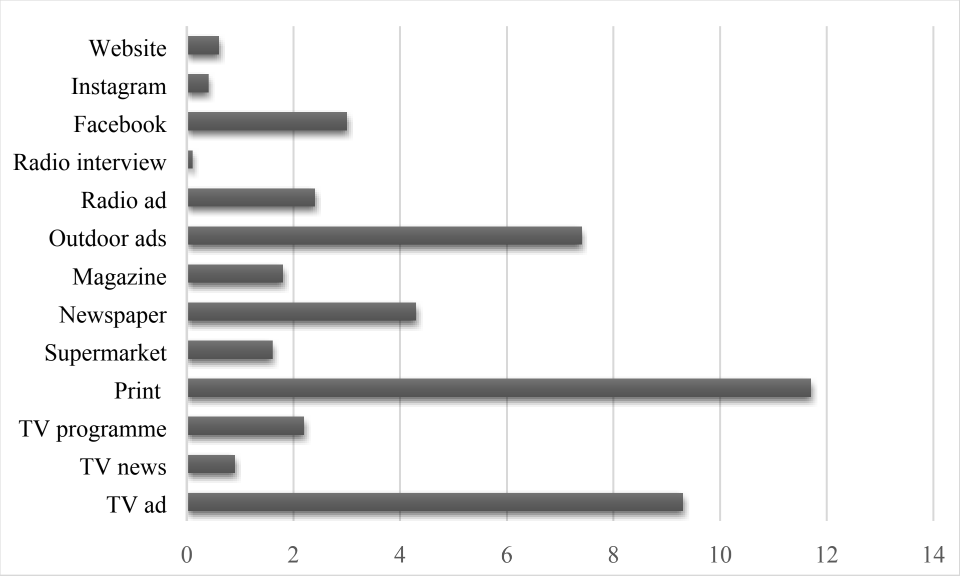

Appendix Figure 1 Advertisement channels through which participants noticed the BCAC-BC advertisements (unprompted)

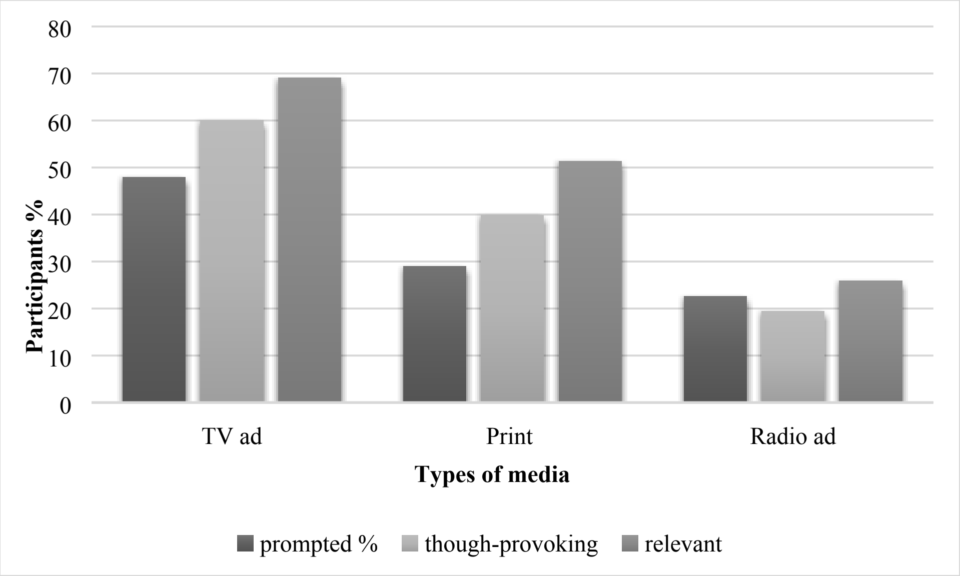

Appendix Figure 2 Advertisement channels through which participants noticed the BCAC-BC advertisements (prompted) and views on materials

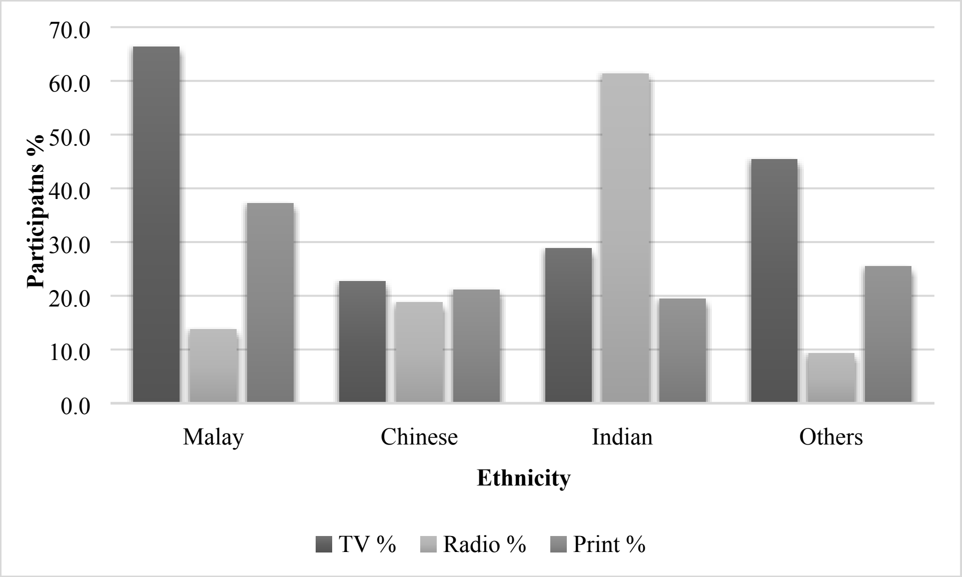

Appendix Figure 3 Difference in campaign material reach between ethnicities

For peer review only

STROBE 2007 (v4) checklist of items to be included in reports of observational studies in epidemiology*
Checklist for cohort, case-control, and cross-sectional studies (combined)

Section/Topic	Item #	Recommendation	Reported on page #
**Title and abstract**	1	(*a*) Indicate the study's design with a commonly used term in the title or the abstract	2
		(*b*) Provide in the abstract an informative and balanced summary of what was done and what was found	2
**Introduction**			
Background/rationale	2	Explain the scientific background and rationale for the investigation being reported	4
Objectives	3	State specific objectives, including any pre-specified hypotheses	4
**Methods**			
Study design	4	Present key elements of study design early in the paper	4,5
Setting	5	Describe the setting, locations, and relevant dates, including periods of recruitment, exposure, follow-up, and data collection	5
Participants	6	(*a*) *Cohort study*—Give the eligibility criteria, and the sources and methods of selection of participants. Describe methods of follow-up<br>*Case-control study*—Give the eligibility criteria, and the sources and methods of case ascertainment and control selection. Give the rationale for the choice of cases and controls<br>*Cross-sectional study*—Give the eligibility criteria, and the sources and methods of selection of participants	4,5
		(*b*) *Cohort study*—For matched studies, give matching criteria and number of exposed and unexposed<br>*Case-control study*—For matched studies, give matching criteria and the number of controls per case	
Variables	7	Clearly define all outcomes, exposures, predictors, potential confounders, and effect modifiers. Give diagnostic criteria, if applicable	5
Data sources/ measurement	8*	For each variable of interest, give sources of data and details of methods of assessment (measurement). Describe comparability of assessment methods if there is more than one group	5
Bias	9	Describe any efforts to address potential sources of bias	5,6
Study size	10	Explain how the study size was arrived at	6
Quantitative variables	11	Explain how quantitative variables were handled in the analyses. If applicable, describe which groupings were chosen and why	6
Statistical methods	12	(*a*) Describe all statistical methods, including those used to control for confounding	6
		(*b*) Describe any methods used to examine subgroups and interactions	6
		(*c*) Explain how missing data were addressed	6
		(*d*) *Cohort study*—If applicable, explain how loss to follow-up was addressed<br>*Case-control study*—If applicable, explain how matching of cases and controls was addressed	

For peer review only

		Cross-sectional study—If applicable, describe analytical methods taking account of sampling strategy	
		(*e*) Describe any sensitivity analyses	
**Results**			
Participants	13*	(a) Report numbers of individuals at each stage of study—eg numbers potentially eligible, examined for eligibility, confirmed eligible, included in the study, completing follow-up, and analysed	7
		(b) Give reasons for non-participation at each stage	7
		(c) Consider use of a flow diagram	
Descriptive data	14*	(a) Give characteristics of study participants (eg demographic, clinical, social) and information on exposures and potential confounders	7
		(b) Indicate number of participants with missing data for each variable of interest	19,20
		(c) *Cohort study*—Summarise follow-up time (eg, average and total amount)	
Outcome data	15*	*Cohort study*—Report numbers of outcome events or summary measures over time	
		Case-control study—Report numbers in each exposure category, or summary measures of exposure	
		Cross-sectional study—Report numbers of outcome events or summary measures	7,8
Main results	16	(*a*) Give unadjusted estimates and, if applicable, confounder-adjusted estimates and their precision (eg, 95% confidence interval). Make clear which confounders were adjusted for and why they were included	8-10, 20,22
		(*b*) Report category boundaries when continuous variables were categorized	19
		(*c*) If relevant, consider translating estimates of relative risk into absolute risk for a meaningful time period	
Other analyses	17	Report other analyses done—eg analyses of subgroups and interactions, and sensitivity analyses	7-10, 20-23
**Discussion**			
Key results	18	Summarise key results with reference to study objectives	9,10
Limitations	19	Discuss limitations of the study, taking into account sources of potential bias or imprecision. Discuss both direction and magnitude of any potential bias	10,11
Interpretation	20	Give a cautious overall interpretation of results considering objectives, limitations, multiplicity of analyses, results from similar studies, and other relevant evidence	9-11
Generalisability	21	Discuss the generalisability (external validity) of the study results	11
**Other information**			
Funding	22	Give the source of funding and the role of the funders for the present study and, if applicable, for the original study on which the present article is based	12

*Give information separately for cases and controls in case-control studies and, if applicable, for exposed and unexposed groups in cohort and cross-sectional studies.

Note: An Explanation and Elaboration article discusses each checklist item and gives methodological background and published examples of transparent reporting. The STROBE checklist is best used in conjunction with this article (freely available on the Web sites of PLoS Medicine at http://www.plosmedicine.org/, Annals of Internal Medicine at http://www.annals.org/, and Epidemiology at http://www.epidem.com/). Information on the STROBE Initiative is available at www.strobe-statement.org.

For peer review only

BMJ Open

The impact of a mass media campaign on breast cancer symptoms awareness and screening uptake in Malaysia: findings from a quasi-experimental study

Journal:	*BMJ Open*
Manuscript ID	bmjopen-2019-036503.R1
Article Type:	Original research
Date Submitted by the Author:	23-Mar-2020
Complete List of Authors:	Schliemann, Desiree ; Queen's University Belfast, Centre for Public Health<br>Htay , Mila; University of Malaya, Department of Social and Preventive Medicine<br>Dahlui, Maznah; University of Malaya, Department of Social and Preventive Medicine<br>Paramasivam, Darishiani; University of Malaya, Department of Social and Preventive Medicine<br>Cardwell, Christopher; Queen's University Belfast Centre for Public Health<br>Ibrahim Tamin, Nor Saleha; Ministry of Health Malaysia Disease Control Division<br>Somasundaram, Saunthari ; National Cancer Society Malaysia<br>Donnelly, Conan; National Cancer Registry<br>Su, Tin ; Monash University - Malaysia Campus, Jeffrey Cheah School of Medicine and Health Sciences<br>Donnelly, Michael; Queen's University Belfast Centre for Public Health
**Primary Subject Heading**:	Public health
Secondary Subject Heading:	Communication, Global health, Oncology
Keywords:	PUBLIC HEALTH, Breast tumours < ONCOLOGY, EDUCATION & TRAINING (see Medical Education & Training)

SCHOLARONE™ Manuscripts

I, the Submitting Author has the right to grant and does grant on behalf of all authors of the Work (as defined in the below author licence), an exclusive licence and/or a non-exclusive licence for contributions from authors who are: i) UK Crown employees; ii) where BMJ has agreed a CC-BY licence shall apply, and/or iii) in accordance with the terms applicable for US Federal Government officers or employees acting as part of their official duties; on a worldwide, perpetual, irrevocable, royalty-free basis to BMJ Publishing Group Ltd ("BMJ") its licensees and where the relevant Journal is co-owned by BMJ to the co-owners of the Journal, to publish the Work in this journal and any other BMJ products and to exploit all rights, as set out in our licence.

The Submitting Author accepts and understands that any supply made under these terms is made by BMJ to the Submitting Author unless you are acting as an employee on behalf of your employer or a postgraduate student of an affiliated institution which is paying any applicable article publishing charge ("APC") for Open Access articles. Where the Submitting Author wishes to make the Work available on an Open Access basis (and intends to pay the relevant APC), the terms of reuse of such Open Access shall be governed by a Creative Commons licence – details of these licences and which Creative Commons licence will apply to this Work are set out in our licence referred to above.

Other than as permitted in any relevant BMJ Author's Self Archiving Policies, I confirm this Work has not been accepted for publication elsewhere, is not being considered for publication elsewhere and does not duplicate material already published. I confirm all authors consent to publication of this Work and authorise the granting of this licence.

For peer review only

Title: The impact of a mass media campaign on breast cancer symptoms awareness and screening uptake in Malaysia: findings from a quasi-experimental study

Authors: Désirée Schliemann[1] (PhD); Mila Nu Nu Htay[2,3] (MMedSc); Maznah Dahlui[2,4] (PhD); Darishiani Paramasivam[2] (MSc), Christopher R. Cardwell[1] (PhD); Nor Saleha Binti Ibrahim Tamin[5] (MPH); Saunthari Somasundaram[6] (MBA), Conan Donnelly[7] (PhD); Tin Tin Su[2,8] (PhD); Michael Donnelly[1] (PhD)

[1] Centre for Public Health and UKCRC Centre of Excellence for Public Health, Queen's University Belfast, Belfast, UK

[2] Centre for Population Health (CePH), Department of Social and Preventive Medicine, University of Malaya, Kuala Lumpur, Malaysia

[3] Department of Community Medicine, Melaka-Manipal Medical College, Manipal Academy of Higher Education (MAHE), Melaka, Malaysia

[4] Facultas Public Health, University Airlangga, Indonesia

[5] Ministry of Health Malaysia, Kuala Lumpur, Malaysia

[6] National Cancer Society Malaysia, Kuala Lumpur, Malaysia

[7] National Cancer Registry, Cork, Ireland

[8] South East Asia Community Observatory (SEACO), Jeffrey Cheah School of Medicine and Health Sciences, Monash University Malaysia

Word count main text: 3451

Corresponding author: Désirée Schliemann / email: d.schliemann@qub.ac.uk/ phone: +44 28 9097 6350/ fax: +44 28 90235900 / ORCID ID: 0000-0002-8746-3002

For peer review only - http://bmjopen.bmj.com/site/about/guidelines.xhtml

Abstract

Objective: To evaluate the impact mass media of a campaign in terms of improving breast cancer (BC) symptoms awareness and screening uptake.

Design: Before- and after- study with comparator groups.

Setting: Selangor State, Malaysia.

Participants: Malaysian women aged ≥40 years (n=676) from randomly selected households.

Intervention: A culturally adapted mass media campaign (TV, Radio, print media and social media).

Primary and secondary outcome measures: The primary endpoint was BC symptom awareness, which was assessed with the *Breast Cancer Awareness Measure* pre- and post-campaign. Secondary outcomes included campaign reach, self-efficacy to notice BC symptoms and clinical outcomes. Clinical breast examination and mammogram screening data were collected from hospitals and clinics.

Results: Most participants recognised at least one of the campaign materials (65.2%). The odds of seeing the campaign were lowest for Chinese women (adjusted OR 0.69, 95% CI 0.42, 1.14) compared to Malays and for women aged ≥70 years (adjusted OR 0.47, 95% CI 0.23, 0.94) compared to younger women. Participants who recognised the campaign were significantly more likely to have improved awareness post-campaign compared to non-recognisers particularly for key symptoms such as 'a lump or thickening in your breast' (88.9% vs 62.1%) and 'discharge or bleeding from nipple' (79.7% vs 55.3%). Improvement in symptoms awareness scores was not associated with socio-demographic variables.

Conclusions: Implementation in Malaysia of an evidence-based mass media campaign from the UK that was culturally adapted appeared to lead to improved awareness about some BC symptoms, though various modes of media communication and perhaps other health education approaches may be required to extend the reach to diverse, multi-ethnic populations and all age groups.

Key words: public health, health promotion, breast cancer, awareness, symptoms, screening, signs, clinical breast examination, mammogram, social media, media campaign.

For peer review only - http://bmjopen.bmj.com/site/about/guidelines.xhtml

Article Summary

Strengths and limitations

- The public health intervention was subjected to a systematic cultural adaptation process and drew upon best available evidence.
- This was the first study to evaluate a mass media campaign designed to improve breast cancer awareness in Malaysia.
- The study outcomes were assessed using psychometrically validated measures that were pilot-tested with the Malaysian multi-ethnic population
- The development and evaluation approach provides a blueprint for public health researchers in other Asian countries.
- The nationwide distribution of the mass media campaign meant that it was not possible to have conventional control groups though the study was able to create internal comparator groups of 'recognisers' vs 'non-recognisers'.

For peer review only

For peer review only - http://bmjopen.bmj.com/site/about/guidelines.xhtml

Introduction

Breast cancer (BC) comprises 17.7% of all cancers in Malaysia[1] and it is the commonest cause of cancer deaths among women in Malaysia[2]. The high cancer mortality rate is due to several factors particularly late detection. For example, between 2007–2011, 43% of women were diagnosed with BC at a late stage (stage 3 and 4)[1] compared to <20% of women in the UK[3], supporting the benefits of early detection interventions that are more commonly implemented in high income countries[4]. Late presentation is due, at least partly, to low cancer awareness. Research indicates that there is a lack of awareness among Malaysian women about common symptoms of BC[5,6], e.g. only 34% of women recognised 'a painless breast lump' as a BC symptom[6]. Other causes of delayed detection and diagnosis include denial, lack of knowledge, negative perceptions of the disease, over-reliance on traditional medicine, misperceived risk, emotional barriers and negative perceptions towards screening[7–9]. Furthermore, current screening guidelines encourage opportunistic biennially screening for breast cancer in Malaysian females aged between 50-74 years[10] and population-based screening is lacking. Nationwide education programmes are encouraged as a first step to improve early detection of breast cancer[11] in settings where population-based cancer screening is unavailable and individuals are required to self-initiate help-seeking when symptoms are experienced. However, robust evaluations of mass media interventions for cancer health promotion in Asia, and particularly Malaysia, are lacking[12]. In high income countries, mass media campaigns have improved symptoms awareness[13,14] and increased the number of BC referrals[15]. Industry and non-governmental organisations (NGOs) exert mass media type efforts regularly to raise awareness about BC but these interventions tend to be short-lived and are not subjected to robust evaluation[16]. In response to this knowledge gap, our collaboration developed a culturally acceptable, evidenced-based mass media campaign for Malaysia - the *Be Cancer Alert Campaign* (BCAC)[17,18]. The primary endpoint of this study was BC symptoms awareness. Secondary outcomes included the reach of the campaign, perceived self-efficacy to detect symptoms, visits to a health care professional to discuss BC symptoms, number of BC screenings undertaken (clinical breast examination (CBE) and mammogram) and the number of BC cases diagnosed.

Methods

The protocol for the evaluation of the BCAC-BC was published previously[17] and is explained here in brief. The Research Ethics Committee, University of Malaya (ID: 2016126–4668) and the National Medical Research Register (ID: NMRR-18-1961-42562), granted ethical approval.

Study population and sampling

For peer review only - http://bmjopen.bmj.com/site/about/guidelines.xhtml

Malaysia comprises three main ethnicities: Malay (69%), Chinese (23%) and Indian (7%)[19]. The sample was drawn from Petaling District (Sungai Buloh and Petaling Jaya), Selangor State, with a multi-ethnic population composition[17]. Trained interviewers visited randomly selected households and invited female residents to participate if they were aged ≥40 years, spoke English or Malay, were able to provide answers without support from others and provided informed consent. Participants were interviewed one-to-twelve weeks before (i.e. July to September 2018) and after (i.e. November 2018 to January 2019) the BCAC-BC implementation.

Intervention

Implementation of the BCAC-BC mass media campaign occurred over a 5-week period (September-October 2018). Appendix Table 1 provides a description of campaign materials**.** Nationwide TV and radio presented advertisements; the study area received print materials (i.e. billboards, street bunting, posters and brochures); the National Cancer Society Malaysia (NCSM) delivered a social media campaign via their Facebook page. All materials contained a link to a bespoke BCAC website and highlighted the NCSM helpline.

Patient and public involvement

This study involved the leading advocacy organisation for cancer prevention and cancer care in Malaysia – NCSM. Representatives from NCSM, the Ministry of Health (MoH), Malaysia and the university researchers worked in partnership to address the research questions. In particular, we involved our partners, members of the public and cancer survivors in focus groups and interviews regarding the preparation of the campaign materials and their delivery and we included cancer survivors in the TV advertisements. Finally, the results of the research were disseminated in collaboration with the NCSM, MoH and cancer survivors.

Data collection

Questionnaire

Pre- and post-campaign surveys were conducted to evaluate the impact of the mass media campaign on BC awareness with questions informed by the *Breast Cancer Awareness Measure* (CAM)[20]. We assessed *Unprompted* BC symptoms knowledge by asking, 'There are many warning signs and symptoms of BC. Please name as many as you can think of'. We assessed *Prompted* knowledge by asking, 'Do you think [symptom] could be a sign for BC?' A total score for unprompted knowledge and

For peer review only - http://bmjopen.bmj.com/site/about/guidelines.xhtml

prompted knowledge, respectively, was calculated by summing the correct answers. Socio-demographic characteristics, cancer history (of respondent) and monthly household income were assessed pre-campaign only. Post-campaign, specific questions assessed campaign reach[21]. Participants were asked whether they noticed the BCAC logo and other materials. The final set of questions asked participants whether or not they found the materials relevant and acceptable; shared/discussed the campaign information and whether or not they or their family and/or friends visited a health professional as a result of seeing the BCAC-BC.

Social media monitoring

The social media activity was monitored daily by an external agency; and it was evaluated in terms of post reach (total number of unique users who saw the advertisement/post on their Facebook feed), interaction (total number of emoji reactions including like, love, smile, wow, sad and angry), amplification (number of shares per post), conversation (number of comments per post) and total engagement (total number of interactions, amplification and conversation per post).

Helpline

Trained nurses recorded (with consent) NCSM Helpline callers who said that they got the helpline number from one of the BCAC campaign materials in terms of date of call, gender of caller, reason for calling and campaign source for the number.

Health service use

Staff from local health clinics and hospitals recorded the number of CBE and mammograms undertaken between July 2018 and January 2019 as well as basic socio-demographic information.

Sample size

It was estimated that 550 participants would provide 80% power to detect, as statistically significant at a 5% level, an increase by 6% in the proportion of individuals who were aware that an unexplained lump or swelling was a symptom of breast cancer[17] using a two-sided McNemar Test.

Data analysis

For peer review only - http://bmjopen.bmj.com/site/about/guidelines.xhtml

The McNemar test assessed pre- and post-campaign proportional differences in knowledge/awareness. Chi-square tests examined associations between recognition of one or more BCAC materials when prompted (i.e. 'BCAC-recognisers' and 'non-recognisers') and BC awareness; and tested associations between BC history or BC screening history and BC symptoms awareness. Logistic regression investigated the relationship between BCAC recognition (yes versus no) and potential explanatory variables. The final model from which adjusted estimates were calculated contained age, gender, ethnicity, marital status, education, monthly family household income, BC history and BC screening history (received BC screening – either CBE or mammogram- in the past two years). Similar models were applied to the outcome, 'knowledge improved' (yes vs no). Logistic regression analyses were repeated using robust standard errors to adjust for potential clustering within households[22] (the results were similar to the results that are presented here). Service utilisation data were charted over the relevant time-periods. All available information was included in the analysis.

Results

The BCAC-BC was implemented as planned (except that the TV advertisement was conducted for four weeks instead of five weeks (Appendix Table 1).

Study population

A total of 992 participants completed the pre-campaign survey and 68% (676/992) completed the post-campaign survey (**Table 1**). Participants who did not complete the follow-up survey could not be reached or refused to participate post-campaign. Forty-one percent of participants who completed both surveys were aged between 40 and 49 years and about 30% of women were aged between 50-59 years. Malays were the most commonly represented ethnic group (51.6 %), followed by Chinese (22.3%) and Indians (17.8%). Most women were married (86.8%); just over half completed secondary education (54.2%); and most (71%) had a monthly low household income of less than RM 4,000[23]. Sixteen women (2.4%) had a personal history of BC and 25.9% underwent a mammogram in the previous two years. Women who completed the follow-up assessment were significantly younger compared to women who completed only the pre-campaign survey. There was a higher proportion of Malay and Indian participants and fewer Chinese participants at follow-up compared to baseline (**Table 1**).

Campaign reach

Eighteen percent of participants reported that they previously saw the BCAC logo. Participants remembered (unprompted) seeing BCAC posters in clinics (11.7%), TV advertisements (9.3%), outdoor

For peer review only - http://bmjopen.bmj.com/site/about/guidelines.xhtml

materials (bunting/billboards, 7.4%), newspaper articles (4.3%) and hearing BCAC radio advertisements (2.4%) (Appendix Figure 1). Furthermore, 26.9% recognised the slogan, '*Don't be shy to check your breast*'. When participants were prompted or shown BCAC materials, 65.2% recognised BCAC materials (i.e. 47.9% recognised the TV advertisements, 29% recognised print materials and 22.6% recognised the radio advertisement). TV advertisements were deemed thought-provoking by 60% and relevant by 69% (Appendix Figure 2). Print materials were thought-provoking for 40% and relevant for 51.3%; fewer participants described radio ads in these terms (19.4% and 25.9%). Almost 40% discussed the advertisements with friends/family and 21.7% stated that they or their family/friends went to see a doctor as a result of seeing the advertisements. TV advertisements were most commonly recognised by Malays (66.4%) followed by 'others' (45.5%). Indian participants (61.3%) recognised radio advertisements more often than other ethnic groups. Print advertisements were recognised by more Malays (37%) compared to between 19.5% and 25.5% of other ethnicities (Appendix Figure 3). Between-ethnic groups differences in campaign reach were confirmed by regression analysis (**Table 2**). The odds that participants saw one or more of the BCAC materials (when prompted) were significantly lower for Chinese and 'others' compared to Malays (adjusted OR 0.25, 95% CI 0.15; 0.40, p<0.001 and OR 0.34, 95% CI 0.18; 0.65, p=0.001). Campaign reach towards people aged ≥70 years appeared to be relatively poor (adjusted OR 0.47, 95% CI 0.23; 0.94, p=0.032).

Thirty-two social media 'posts' were shared through Facebook and 11/32 posts were boosted. The boosted posts reached significantly more people than 'ordinary' posts. The boosted post describing BC symptoms in Malay achieved the highest reach (reach: 202,430; total engagement: 4,498 and shares: 1,379) compared to the same post in English (reach: 18,012, total engagement: 126 and shares: 25). The second most popular post showed highlights of the campaign launch (reach: 19,071, total engagement: 816 and shares: 45) and a post on breast self-examination in English (reach: 15,673, total engagement: 322 and shares: 87). Posts that were not boosted reached between 1,495 and 486 Facebook users whereas posts that were boosted reached between 202,430 and 3,412 users.

The helpline received five calls from women (aged 22-42 years old) who called after seeing a BCAC advertisement; 4/5 women reported experiencing potential BC symptoms and one woman had had a negative biopsy result and enquired about follow-up appointments.

Campaign impact

Knowledge improved significantly for six BC symptoms (unprompted): 'Pain in one of your breasts or armpits' (18.9% vs 23.2%), 'discharge or bleeding from nipple' (10.1% vs 16.4%), 'nipple rash' (1.3% vs 3.6%), 'redness of your breast skin' (1.5% vs 7.2%), 'lump or thickening under armpit' (3.4% vs 7.5%) and 'changes in the shape of your breast or nipple' (1.5% vs 3.7%) significantly at follow-up (**Table 3**). Knowledge/awareness (prompted) increased significantly for three BC symptoms: 'change

For peer review only - http://bmjopen.bmj.com/site/about/guidelines.xhtml

in the position of your nipple' (58.7% vs 67.3%), 'pain in one of your breasts or armpits' (72.5% vs 77.5%) and 'redness of your breast skin' (54.9% vs 62.4%). Overall symptom knowledge/awareness scores (prompted) improved significantly at follow up (pre-Mean 7.45 (SD: 3.05) and post-Mean 7.84 (SD: 2.86)). BCAC recognisers identified on average a higher number of BC symptoms at follow-up compared to baseline (pre-Mean 7.68 (SD: 2.89) and post-Mean 8.26 (SD: 2.46)). There was some evidence that the improvement in awareness by BCAC recognisers was higher compared to non-recognisers (Mean change 0.59 (SD: 3.29) vs Mean change -0.24 (SD: 3.88)). **(**Appendix Table 2**).**

A significantly higher proportion of BCAC recognisers compared to non-recognisers who were not aware of BC symptoms at baseline, improved their knowledge (unprompted) about the symptom 'a lump or thickening in your breast' at follow-up (55.9% vs 41.0%) (**Table 3**). Furthermore, significantly more BCAC recognisers improved their awareness (prompted) about the following symptoms 'change in the position of your nipple' (62.7% vs 41.6%), 'discharge or bleeding from nipple' (79.7% vs 55.3%), 'a lump or thickening in your breast' (88.9% vs 62.1%) and 'changes in the size of your breast or nipple' (67.2% vs 46.2%) compared to non-recognisers. The proportion of women who were very/fairly confident that they would recognise a BC symptom increased at follow-up (58.9% vs 68.9%) (**Table 3**) and there was no significant difference between BCAC recognisers and non-recognisers (63.7% vs 54.4%). Pre-campaign, 96.1% of women reported that they would see their doctor within two weeks if they noticed a BC symptom and there was no change at follow-up. Regression analysis indicated that none of the socio-demographic or campaign recognition variables exerted a marked influence on BC symptoms awareness post-campaign after adjustment (**Table 4**).

From July 2018 to January 2019, 29,000 CBEs were performed (**Figure 1**) as well as 2,051 mammograms (**Figure 2**). More mammograms and CBEs were conducted in October (337 and 47,892), January (335 and 4,978) and July (331 and 4,415) compared to other months. Most CBEs were performed on Malay ethnic women (85%) (Appendix Table 3) and women aged 20-29 years (58%) and 30-39 years (37%). Mammograms were undertaken for Malays (56%), Chinese (27%) and Indians (16%).

Discussion

Research-informed BC mass media campaigns with robust evaluation are lacking in Asian countries like Malaysia[16]. The need to improve BC awareness was confirmed by the limited symptoms awareness and low confidence to notice symptoms that were identified in the pre-campaign survey results. The majority of women noticed the BCAC-BC materials, in particular, the TV advertisements (even though numerous BC awareness activities took place in October as part of BC awareness month); and they found the culturally adapted campaign materials acceptable and relevant to their life circumstances. The salience of TV advertisements as the most commonly recognised campaign material is in keeping with

For peer review only

For peer review only - http://bmjopen.bmj.com/site/about/guidelines.xhtml

other robust evaluations[24,25]. Furthermore, awareness about individual BC symptoms was higher amongst BCAC-recognisers compared to non-recognisers. The high reach and improvement in symptoms awareness suggested that the campaign impacted in terms of improving awareness about some BC symptoms.

Campaign reach varied between ethnicities though improved awareness of BC symptoms did not appear to vary across socio-demographic variables including ethnicity. The differential reach that was experienced by ethnic groups was similar to the findings from the BCAC-CRC campaign in Malaysia[26]. Different attitudes and behaviours across ethnic groups regarding help-seeking may help to explain the apparent discrepancy. For example, USA health survey participants who did not seek health information in the media and preferred to trust their health service provider tended to be older and have a lower socio-economic status[27]. Mass media as a public health programme or intervention requires careful tailoring to maximise the reach and impact of given public health messages.

Although the BCAC-BC had greater reach compared to the BCAC-CRC, the BCAC-CRC appeared to be more successful in terms of improving symptoms awareness[26], perhaps, because of the higher pre-campaign symptom knowledge/awareness (prompted and unprompted) among the BCAC-BC sample compared to BCAC-CRC participants. Awareness improved significantly at follow-up for three prompted BC symptoms whereas awareness about all prompted CRC symptoms improved. The success of the BCAC-BC was due partly to the systematic cultural adaptation process[18] that was undertaken in order to address, for example, the need to refer to breast-related issues with contextual sensitivity and to include only information about a lump. Although a lump appeared already to be a commonly known symptom, it was unacceptable culturally to present other symptoms of BC. Such restrictions did not apply to the BCAC-CRC.

BC is the best-known cancer site in most countries. It was the most common cancer reported in major online newspapers in the USA[28] and it is the most 'promoted' cancer in Malaysia[16]. Also, online information-seeking behaviour related to BC is higher during October (BC awareness month)[29,30]. Findings from the BCAC social media campaigns demonstrated higher reach and engagement for the BC social media posts compared to the CRC posts (i.e. highest reach of CRC-related post: 92,678 users vs BC-related post: 202,430 users; highest engagement with CRC-related post: 2,065 users vs BC-related post: 4,498 users)[26]. CRC campaign material about symptoms may be perceived to be less pleasant to share compared to BC symptoms. Overall, it may not be surprising that BC awareness levels are higher than other cancer sites such as CRC and that the gains from a mass media campaign directed towards BC would be smaller given this elevated baseline awareness. It might be more impactful to reserve the use of mass media campaigns for areas where there is a known low level of awareness and knowledge. The number of calls reported by NCSM as a result of both BCAC campaigns was low

For peer review only - http://bmjopen.bmj.com/site/about/guidelines.xhtml

compared to caller volume reported in other campaign evaluations, e.g. a print and online media campaign targeting low-income African American women for 2.5 months received 97 calls[31].

The apparently high number of CBEs was encouraging and may suggest good implementation of CBE guidelines among younger women (i.e. CBE every three years) but not regarding women aged ≥40 years (5.4%) (i.e. an annual CBE)[32]. Previous research on CBE uptake in Malaysia found similar CBE rates across age groups[33]. The fact that data reflects public health clinic activity may explain why most CBEs were performed on Malay women (85%) - i.e. public health clinics were utilised mainly by Malays while Chinese tended to visit private clinics for screening activity though they may avail of public health care depending on costs). Mammogram activity in this study did not appear to be affected by ethnicity and were conducted among women aged ≥40 years (98.4%) which is in line with BC screening guidelines (i.e. mammograms biennially for women aged 50-74 years)[10]. Yet, the relatively low number of mammograms may highlight gaps in secondary BC prevention and care in Malaysia. Health service use findings from the *Be Clear on Cancer* campaign in England for women aged 70 years and older suggested that though screening referrals increased, the number of cancer cases detected did not increase thereby raising questions about the value of the campaign in the face of the higher workload and low conversion rate[15]. Findings from studies in the USA present mixed results about improved screening uptake. For example, one study found that screening activity in November (following BC awareness month) increased during the 90s when BC awareness and screening were low; however, increments in screening activity of this kind lessened with time as overall screening levels reached a high threshold[34]. The USA Health Communication Survey found that TV viewing (news or entertainment) prompted health information-seeking behaviour beyond socio-demographic variables and influenced cancer screening behaviour[35]. Data covered only seven months and CBE data and private clinic data were unavailable. Mindful of service data limitations, this is the first study to present CBE and mammogram activity over a series of consecutive months in Malaysia. The highest number of mammograms and CBEs were conducted in July, October and January. October is 'breast cancer awareness month' and June and December are holiday months. Although a public awareness BC campaign may increase screening rates, due to wide-spread routine screening activity throughout the year, campaigns may lose impact regarding the detection BC cases (i.e. people at low- or medium-risk and the 'worried well' may be more likely to respond to cancer awareness campaigns)[34,36]. Breast cancer campaign activities other than those related to the BCAC-BC may also have influenced participants' responses; and this potential limitation needs to be kept in mind when interpreting findings regarding the improvement of BC awareness. In addition, participants may have improved their knowledge due to the pre-campaign survey though the results from the comparative analysis of BCAC recognisers and non-recognisers did not support this interpretation.

A recent report[37] stated that evidence about the role and effectiveness of media channels in public health campaigns is limited due, partly and for obvious reasons, to a lack of controlled studies and randomised

For peer review only - http://bmjopen.bmj.com/site/about/guidelines.xhtml

control trials. There have been very few mass media campaigns in Asia that have been scientifically evaluated[12]. This study afforded a degree of control by creating and comparing internal groups based on whether or not they recognised the campaign. The suggestion that longer, more intensive campaigns would be more impactful[37,38] is a reasonable one. However, our evaluation demonstrated that even with a modest and short-term campaign, it is possible to improve knowledge about BC symptoms and influence help-seeking behaviour. Of course, the long-term impact of the campaign and sustainability of the improvements are unknown.

Conclusion

The BCAC-BC study addresses a knowledge gap and adds important insights into the impact of mass media for breast cancer health promotion as well as implementation challenges related to (South East) Asia. Overall, the findings presented here favour positively the use of mass media in breast health promotion. Future campaigns may be enhanced via increased collaboration with disciplines such as social marketers and health journalism[39]. There appears to be a need for mass media campaigns to be tailored to particular sub-populations or hard to reach subgroups, especially in the context of multi-cultural societies. Women aged ≥70 years or above for example, were less likely to observe or notice the BCAC-BC materials and to have received a mammogram (5%). Targeting older women and women from ethnic minorities may be an important consideration in efforts to improve the reach of future BC awareness activities in Malaysia[15].

Declarations

Ethics approval and consent to participate

Ethics approval for the study was granted by the Medical Research Ethics Committee, University Malaya Medical Centre (ID: 2016126–4668) and by the National Medical Research Register (NMRR-18-1961-42562). Informed consent to participate was provided by all study participants. The study was performed in accordance with the Declaration of Helsinki.

Consent for publication

Consent for publication was signed by all study participants.

Data availability

For peer review only

Data can be made available for inspection upon request.

Conflict of interest

The authors declare no conflict of interest.

Funding

This study is funded by UK MRC-Newton Ungku Omar Funding (MR/P013910/1). The collaborative grant application was subjected to peer-review by individual academic reviewers and the final decision about funding was made by an expert panel. The funder had no role in the design of the study, collection, analysis, and interpretation of data or in writing the manuscript.

Author contributions

MDo and TTS conceptualised and planned the project and are the Co-PIs of the successful grant award from UK MRC-Newton Ungku Omar Fund. CD conducted the power calculation and provided guidance on the study design. DS, MNNH, DP, TTS and MDa planned and coordinated the study and data collection. SS guided the BCAC campaign design and implementation and NSBIT guided the collection of health service data. MNNH collected data from the hospitals and clinics. DS and CC conducted the statistical analysis. DS drafted the manuscript. MDo led the editing and refinement of the manuscript. All authors contributed to, reviewed and approved the final manuscript.

Acknowledgements

We would like to acknowledge all involved in the development of the Be Cancer Alert Campaign materials and would like to acknowledge that the Be Cancer Alert Campaign materials were adapted from materials produced by the Public Health Agency, Northern Ireland for the Be Cancer Aware Campaign. We would also like to thank Assoc. Prof. Siew Yim Loh who assisted in the training of data collectors and the distribution of campaign materials. Furthermore, we thank the Department of Statistics Malaysia for providing the randomly selected households. We would like to acknowledge Datin Dr. Premila A/P Kanagasabai (Selayang hopsital)Dr. Yun Sii Ing (Sg Buloh hospital), Dr. Farah Nadrah Mohd Nasir (Sg Buloh Hospital), Dr Hazwan Amzar (Sungai Buloh), Dr Ainur Syahidza Bt Abd Manan (NCD Unit, PKD Petaling), Dr Nurhaidi Bt Abdullah (KK Kota Damansara), Dr. Nur Es Naini (KK Kelana Jaya), Dr. Ee Li Min (KK Taman Medan), and Dr Tengku Nor Shafadilah Bt Tg

For peer review only - http://bmjopen.bmj.com/site/about/guidelines.xhtml

Mohd Nordin (KK Paya Jaras) for providing data on breast cancer screening and all participants for completing the surveys.

References

1 Azizah AM, Nor Saleha I, Noor Hashimah A, Asmah Z, Mastulu W. Malaysian National Cancer Registry Report 2007-2011. 2013 doi:MOH/P/KN/01.16(AR).

2 International Agency for Research on Cancer. GLOBOCAN. 2018http://gco.iarc.fr/today/home.

3 Cancer Research UK. Breast cancer incidence (invasive) statistics. 2017.https://www.cancerresearchuk.org/health-professional/cancer-statistics/statistics-by-cancer-type/breast-cancer/incidence-invasive#heading-Three (accessed 24 Jul2019).

4 Koo MM, Swann R, Mcphail S, Abel GA, Elliss-brookes L, Rubin GP *et al.* Presenting symptoms of cancer and stage at diagnosis: evidence from a cross-sectional, population-based study. *Lancet Oncol* 2019; **2045**: 1–7.

5 Al-Dubai SAR, Qureshi AM, Saif-Ali R, Ganasegeran K, Alwan MR, Hadi JIS. Awareness and knowledge of breast cancer and mammography among a group of malaysian women in shah alam. *Asian Pacific J Cancer Prev* 2011; **12**: 2531–2538.

6 Abdul Hadi M, Hassali MA, Shafie AA, Awaisu A. Knowledge and perception of breast cancer among women of various ethnic groups in the state of penang: A cross-sectional survey. *Med Princ Pract* 2010; **19**: 61–67.

7 Khan TM, Leong JPY, Ming LC, Khan AH. Association of knowledge and cultural perceptions of Malaysian women with delay in diagnosis and treatment of breast cancer: A systematic review. *Asian Pacific J Cancer Prev* 2015; **16**: 5349–5357.

8 Yu FQ, Murugiah MK, Khan AH, Mehmood T. Meta-synthesis exploring barriers to health seeking behaviour among Malaysian breast cancer patients. *Asian Pac J Cancer Prev* 2015; **16**: 145–152.

9 Mahmud A, Aljunid SM. The uptake of mammogram screening in Malaysia and its associated factors: A systematic review. *Med J Malaysia* 2018; **73**: 202–211.

10 Ministry of Health Malaysia. Clinical Practice Guidelines Management of Breast Cancer Malaysia 2010. Putrajaya, 2010http://www.moh.gov.my/penerbitan/CPG2017/6915.pdf.

11 Talib Z, Amersi F, Harit A, Saleh M. Promoting Breast Cancer Awareness and Clinical Breast Examination in the LMIC: Experiences from Tajikistan, Pakistan and Kenya. *Curr Breast Cancer Rep* 2019; : 152–157.

For peer review only - http://bmjopen.bmj.com/site/about/guidelines.xhtml

12 Schliemann D, Su TT, Paramasivam D, Treanor C, Dahlui M, Loh SY *et al.* Effectiveness of Mass and Small Media Campaigns to Improve Cancer Awareness and Screening Rates in Asia: A Systematic Review. *JCO Glob Oncol* 2019; **5**: 1–20.

13 Public Health Agency. Evaluation Report Breast Campaign. 2016.

14 Croager EJ, Gray V, Pratt IS, Slevin T, Pettigrew S, D'arcy Holman C *et al.* Find Cancer Early: Evaluation of a community education campaign to increase awareness of cancer signs and symptoms in people in regional Western Australians. *Front Public Heal* 2018; **6**: 4–9.

15 Mazari FAK, Holt S, Azmy IA. The impact of "Be Clear on Cancer" campaign on breast care services provided by a specialist oncoplastic unit – A retrospective case control study. *Int J Surg* 2017; **47**: 54–60.

16 Loh SY, Somasundaram S, Su TT. A Review of Cancer Awareness in Malaysia – What ' s Next ? *Open Access J Cancer Oncol* 2017; **1**: 1–6.

17 Schliemann D, Donnelly M, Dahlui M, Loh SY, Tamin Ibrahim NSB, Somasundaram S *et al.* The 'Be Cancer Alert Campaign': protocol to evaluate a mass media campaign to raise awareness about breast and colorectal cancer in Malaysia. *BMC Cancer* 2018; **18**: 881.

18 Schliemann D, Su TT, Paramasivam D, Ibrahim Tamin NSB, Dahlui M, Loh SY *et al.* The systematic cultural adaptation of a UK public health cancer awareness raising programme for Malaysia: The Be Cancer Alert Campaign. *Transl Behav Med* 2019.

19 Department of Statistics Malaysia. Current Population Estimates, Malaysia, 2017-2018. 2018.https://www.dosm.gov.my/v1/index.php?r=column/cthemeByCat&cat=155&bul_id=c1pqTnFjb29HSnNYNUpiTmNWZHArdz09&menu_id=L0pheU43NWJwRWVSZklWdzQ4TlhUUT09 (accessed 28 Jan2019).

20 Cancer Research UK KCL& UCL. Breast Module of the Cancer Awareness Measure. 2010http://www.cancerresearchuk.org/sites/default/files/health_professional_breast_cam_toolkit_09.02.11.pdf.

21 Public Health Agency. Evaluation Report Primer and Lung Campaigns. 2015.

22 Kirkwood B, Sterne J. *Essential Medical Statistics (2nd Edition)*. Blackwell Publishing, 2003.

23 Ng A, Firouz AMM, Khalidi JR, Muhtar MA, Tumin SA, Man TK *et al.* The state of households 2018: different realities. 2018.

24 Public Health England. Evaluation of the Be Clear on Cancer lung cancer awareness campaign regional pilot, East and West Midlands. London, 2015.

For peer review only - http://bmjopen.bmj.com/site/about/guidelines.xhtml

25 PHA. Evaluation Report Breast Campaign. 2016.

26 Schliemann D, Paramasivam D, Dahlui M, Cardwell CR, Somasundaram S, Ibrahim Tamin NSB *et al.* Change in public awareness of colorectal symtpoms following the Be Cancer Alert Campaign in the multi-ethnic population of Malaysia. *BMC Cancer* 2020.

27 Ramanadhan S, Viswanath K. Health and the information nonseeker: a profile. *Health Commun* 2006; **20**: 131–139.

28 Hurley RJ, Riles JM, Sangalang A. Online Cancer News: Trends Regarding Article Types, Specific Cancers, and the Cancer Continuum. *Health Commun* 2014; **29**: 41–50.

29 Glynn RW, Kelly JC, Coffey N, Sweeney KJ, Kerin MJ. The effect of breast cancer awareness month on internet search activity - a comparison with awareness campaigns for lung and prostate cancer. *BMC Cancer* 2011; **11**: 442.

30 Phillips CA, Leahy AB, Li Y, Shapira MM, Bailey L. Relationship Between State-Level Google Online Search Volume and Cancer Incidence in the United States: Retrospective Study. *J Med Internet Res* 2018; **20**.

31 Wallington SF, Oppong B, Iddirisu M, Adams-Campbell LL. Developing a Mass Media Campaign to Promote Mammography Awareness in African American Women in the Nation's Capital. *J Community Health* 2018; **43**: 633–638.

32 Ministry of Health Malaysia. Garis Panduan Program Pengesanan Awal Kanser Payudara Kebangsaan. Malaysia: Bahagian Pembangunan Kesihatan Keluarga, Kementerian Kesihatan Malaysia; 2011. Report No.: MOH/K/ASA/44.11(GU). Putrajaya, 2011.

33 Farid NDN, Aziz NA, Al-Sadat N, Jamaludin M, Dahlui M. Clinical breast examination as the recommended breast cancer screening modality in a rural community in Malaysia; what are the factors that could enhance its uptake? *PLoS One* 2014; **9**: 1–6.

34 Jacobsen GD, Jacobsen KH. Health awareness campaigns and diagnosis rates: Evidence from National Breast Cancer Awareness Month. *J Health Econ* 2011; **30**: 55–61.

35 Li W, Watts J, Tan N. From Screen to Screening: Entertainment and News Television Media Effects on Cancer Screening Behaviors. *J Health Commun* 2019; **24**: 385–394.

36 Karabay O, Hasbahceci M, Kadioglu H. Impact of breast cancer awareness month on detection of breast cancer in a private hospital. *J Int Med Res* 2018; **46**: 619–625.

37 Stead M, Angus K, Langley T, Katikireddi SV, Hinds K, Hilton S *et al.* Mass media to communicate public health messages in six health topic areas: a systematic review and other reviews of the evidence. *Public Heal Res* 2019; **7**: 1–206.

For peer review only - http://bmjopen.bmj.com/site/about/guidelines.xhtml

38 Wakefield MA, Loken B, Hornik RC. Use of mass media campaigns to change health behaviour. *Lancet* 2010; **376**: 1261–1271.

39 Hannon P, Lloyd GP, Viswanath K, Smith T, Basen-Engquist K, Vernon SW *et al.* Mass media and marketing communication promoting primary and secondary cancer prevention. *J Health Commun* 2009; **14**: 30–37.

For peer review only

Table legends

Table 1 Socio-demographic characteristics of respondents regarding the breast cancer campaign (n=676)

Table 2 The relationship between the socio-demographic characteristics of respondents and their recognition of any aspect of the BCAC-BC campaign [a]

Table 3 Be Cancer Alert Campaign – Breast Cancer awareness pre- and post-campaign (n=676)

Table 4 Improvement in overall prompted symptom awareness by socio-demographic characteristics and recognition of BCAC-BC advertisements (binary logistic regression)

Figure legends

Figure 1 CBEs undertaken in clinics between July 2018 and January 2019

Figure 2 Mammograms undertaken in hospitals between July 2018 and January 2019

Appendix

Appendix Table 1 Information about all campaign activities and media used during BCAC-BC

Appendix Table 2 Change in average prompted knowledge score

Appendix Table 3 Number of CBEs (n=29,000) and mammograms (n=2 051) undertaken by ethnicity and age (July 2018-January 2019)

Appendix Figure 1 Advertisement channels through which participants noticed the BCAC-BC advertisements (unprompted)

Appendix Figure 2 Advertisement channels through which participants noticed the BCAC-BC advertisements (prompted) and thoughts on materials

Appendix Figure 3 Difference in campaign material reach between ethnicities

For peer review only - http://bmjopen.bmj.com/site/about/guidelines.xhtml

For peer review only

Tables

Table 1 Socio-demographic characteristics of respondents pre- and post-campaign

	Pre only *n (%)* *n=316*	Pre and post *n (%)* *n=676*
**Age**		
40-49 years	104 (32.9)	274 (41.0)
50-59 years	102 (32.3)	199 (29.8)
60-69 years	67 (21.2)	137 (20.5)
≥70 years	41 (13.0)	58 (8.7)
**Ethnicity**		
Malay	150 (47.5)	349 (51.6)
Chinese	102 (32.3)	151 (22.3)
Indian	39 (12.3)	120 (17.8)
Others	25 (7.9)	56 (8.3)
**Religion**		
Islam	169 (53.5)	406 (60.1)
Christianity	26 (8.2)	50 (7.4)
Buddhism	81 (25.6)	109 (16.1)
Hinduism	31 (9.8)	100 (14.8)
Others	3 (0.9)	6 (0.8)
**Marital status**		
Married	265 (83.9)	587 (86.8)
Single	51 (16.1)	89 (13.2)
**Education**		
No formal education	45 (14.2)	88 (13.0)
Primary	52 (16.5)	97 (14.4)
Secondary	158 (50.0)	366 (54.2)
Tertiary	59 (18.7)	124 (18.4)
**Family income**		
<RM 4,000	189 (59.8)	457 (71.2)
RM 4,000-10,000	72 (22.8)	140 (21.8)
>RM 10,000	23 (7.3)	45 (7.0)
**BC history** (self only)		
No	307 (97.2)	660 (97.6)
Yes	9 (2.8)	16 (2.4)
**BC screening history** [d]		
No	222 (70.3)	498 (75.5)
Yes	94 (29.7)	175 (25.9)

[a] Participants who are widowed, divorced and who never married
[b] No formal education – includes never schooled/ never completed primary school; primary education – includes completed primary school; secondary education – includes completed form 3/ completed form 5/ certificate/ A-level/ STPM/ HSC; tertiary education – includes diploma/ bachelor degree/ post-graduate degree
[c] Income of all household member combined
[d] BC screening history refers to mammogram in the past 2 years
Missing information: Age (n=3), Religion (n=5), Monthly family income (n=34), BC screening history (n=3)

For peer review only - http://bmjopen.bmj.com/site/about/guidelines.xhtml

Table 2 The relationship between the socio-demographic characteristics of respondents and their recognition of any aspect of the BCAC-BC campaign[a]

	n (%)	OR (95% CI) (unadjusted)	*P*	OR (95% CI) (adjusted)[b]	*P*
**Age**					
40-49 years	198/274 (72.3)	*Reference*		*Reference*	
50-59 years	132/199 (66.3)	0.72 (0.48, 1.09)	0.120	0.74 (0.47, 1.16)	0.187
60-69 years	78/137 (56.9)	0.48 (0.31, 0.75)	0.001	0.65 (0.39, 1.10)	0.107
≥70 years	28/58 (48.3)	0.33 (0.18, 0.60)	<0.001	0.47 (0.23, 0.94)	0.032
**Ethnicity**					
Malay	265/349 (75.9)	*Reference*		*Reference*	
Chinese	68/151 (45.0)	0.25 (0.16, 0.37)	<0.001	0.25 (0.15, 0.40)	<0.001
Indian	81/120 (67.5)	0.62 (0.39, 0.99)	0.046	0.69 (0.42, 1.14)	0.145
Others	27/56 (48.2)	0.29 (0.16, 0.52)	<0.001	0.34 (0.18, 0.65)	0.001
**Marital Status**					
Married	380/566 (67.1)	*Reference*		*Reference*	
Single	61/86 (70.9)	1.19 (0.73, 1.96)	0.484	1.26 (0.72, 2.20)	0.422
**Education**					
No formal education	46/88 (52.3)	*Reference*		*Reference*	
Primary	57/97 (58.8)	1.34 (0.74, 2.44)	0.333	1.33 (0.68, 2.57)	0.404
Secondary	255/366 (69.7)	2.14 (1.32, 3.48)	0.002	1.65 (0.93, 2.91)	0.085
Tertiary	82/124 (66.1)	1.99 (1.11, 3.55)	0.021	1.98 (0.93, 4.18)	0.075
**Monthly family income**					
<RM 4,000	311/443 (70.2)	*Reference*		*Reference*	
RM 4,000 -10,000	82/135 (60.7)	0.66 (0.44, 0.98)	0.040	0.73 (0.46, 1.18)	0.202
RM > 10,000	27/43 (62.8)	0.72 (0.37, 1.37)	0.315	0.88 (0.41, 1.86)	0.728
**BC history**					
No	430/660 (65.2)	*Reference*		*Reference*	
Yes	11/16 (68.8)	1.05 (0.36, 3.07)	0.923	1.21 (0.37, 3.98)	0.754
**BC screening history** [c]					
No	337/510 (66.1)	*Reference*		*Reference*	
Yes	103/163 (63.2)	0.93 (0.63, 1.36)	0.705	1.03 (0.67, 1.60)	0.893

n- number of participants 'reached' or who reported that they saw (one or more parts of) the campaign divided by the total number of survey participants

[a] This includes participants who reported that they have been exposed to either the BCAC-BC TV, radio and/or print materials when prompted with the advertisement at follow-up

[b] adjusted for age, ethnicity, marital status, education, monthly family income, BC history and BC screening history

[c] If people replied 'yes' to BC history and BC screening history, they were coded as 'yes' for BC history and 'no' for BC screening history in this model

For peer review only

Table 3 Be Cancer Alert Campaign – Breast Cancer awareness pre- and post-campaign (n=676)

Survey question	**Pre** n (%)	**Post** n (%)	***P*** *(McNemar)*	**Knowledge improvement in BCAC recognisers**[a] n (%)	**Knowledge improvement in BCAC non-recognisers**[b] n (%)	***P*** *(chi-square)*
**Signs and symptoms (unprompted)**						
Change in the position of your nipple	9 (1.3)	7 (1.0)	0.804	7/435 (1.6)	0/208 (0.0)	0.152
Pulling in your nipple	7 (1.0)	16 (2.4)	0.064	11/438 (2.5)	3/207 (1.4)	0.565
Pain in one of your breasts or armpits	128 (18.9)	157 (23.2)	0.041	74/351 (21.1)	27/179 (15.1)	0.122
Puckering or dimpling of your breast skin	4 (0.6)	7 (1.0)	0.549	5/439 (1.1)	2/210 (1.0)	0.999
Discharge or bleeding from your nipple	68 (10.1)	111 (16.4)	<0.001	55/401 (13.7)	19/188 (10.1)	0.272
A lump or thickening in your breast	440 (65.1)	437 (64.6)	0.897	80/143 (55.9)	34/83 (41.0)	0.042
Nipple rash	9 (1.3)	24 (3.6)	0.012	19/437 (4.3)	4/207 (1.9)	0.188
Redness of your breast skin	10 (1.5)	49 (7.2)	<0.001	35/436 (8.0)	11/207 (5.3)	0.279
Lump or thickening under your armpit	23 (3.4)	51 (7.5)	0.001	35/426 (8.2)	10/205 (4.9)	0.174
Changes in the size of your breast or nipple	12 (1.8)	18 (2.7)	0.327	13/435 (3.0)	2/206 (9.7)	0.194
Changes in the shape of your breast or nipple	10 (1.5)	25 (3.7)	0.015	16/434 (3.7)	8/208 (3.8)	1.000
**Signs and symptoms (prompted)**						
Change in the position of your nipple	397 (58.7)	455 (67.3)	<0.001	106/169 (62.7)	42/101 (41.6)	0.001
Pulling in your nipple	391 (57.8)	397 (58.7)	0.745	85/182 (46.7)	32/92 (34.8)	0.079
Pain in one of your breasts or armpits	490 (72.5)	524 (77.5)	0.029	81/110 (44.5)	45/69 (65.2)	0.302
Puckering or dimpling of your breast skin	379 (56.1)	380 (56.2)	0.999	100/187 (53.5)	43/97 (44.3)	0.181
Discharge or bleeding from your nipple	557 (82.4)	560 (82.8)	0.814	55/69 (79.7)	26/47 (55.3)	0.009
A lump or thickening in your breast	598 (88.5)	612 (90.5)	0.211	40/45 (88.9)	18/29 (62.1)	0.014
Nipple rash	345 (51.0)	367 (54.3)	0.227	111/213 (52.1)	46/109 (42.2)	0.117
Redness of your breast skin	371 (54.9)	422 (62.4)	0.003	112/193(58.0)	47/103 (45.6)	0.055
Lump or thickening under your armpit	549 (81.2)	566 (83.7)	0.213	54/68 (79.4)	34/54 (63.0)	0.070
Changes in the size of your breast or nipple	476 (70.4)	497 (73.5)	0.173	78/116 (67.2)	36/78 (46.2)	0.005
Changes in the shape of your breast or nipple	486 (71.9)	511 (75.6)	0.105	80/114 (70.2)	38/69 (55.1)	0.056
				**Improved attitudes in recognisers**[a]	**Improved attitudes in non-recogniser**[b]	
How confident are you that you would notice a BC symptom? *(those very confident/ fairly confident)*	391 (58.9)	439 (68.9)	<0.001	100/157 (63.7)	56/103 (54.4)	0.170
How soon would you go and see a doctor if you noticed a BC sign/symptom? *(those within 2 weeks)*	614 (96.1)	637 (97.1)	0.337	19/20 (95.0)	13/14 (92.9)	0.999

Missing information: confidence BL n=12; confidence FU n = 39; delayed help seeking BL n=20; delayed help seeking FU n=37

[a] This includes participants who reported that they have been exposed to either the BCAC-BC TV, radio and/or print materials when prompted at follow-up

[b] This includes all participants who reported that they have not seen any of the BCAC-BC TV, radio and/ or print materials when prompted at follow-up

For peer review only - http://bmjopen.bmj.com/site/about/guidelines.xhtml

Table 4 Improvement in overall prompted symptom awareness by socio-demographic characteristics and recognition of BCAC-BC advertisements (binary logistic regression)

	n (%)	OR (95% CI) (unadjusted)	*P*	OR (95% CI) (adjusted)[a]	*P*
**Age**					
40-49 years	122/274 (44.5)	*Reference*		*Reference*	
50-59 years	101/199 (50.8)	1.28 (0.89, 1.85)	0.181	1.36 (0.91, 2.04)	0.130
60-69 years	60/137 (43.8)	0.97 (0.64, 1.47)	0.888	0.97 (0.60, 1.56)	0.890
≥70 years	26/58 (44.8)	1.01 (0.57, 1.79)	0.966	0.99 (0.51, 1.93)	0.982
**Ethnicity**					
Malay	162/349 (46.4)	*Reference*		*Reference*	
Chinese	63/151 (41.7)	0.83 (0.56, 1.22)	0.333	0.82 (0.51, 1.33)	0.420
Indian	58/120 (48.3)	1.08 (0.71, 1.64)	0.717	1.26 (0.75, 2.12)	0.392
Others	30/56 (53.6)	1.33 (0.76, 2.35)	0.321	1.27 (0.67, 2.41)	0.461
**Marital Status**					
Married	270/587 (46.0)	*Reference*		*Reference*	
Single	43/89 (48.3)	1.10 (0.70, 1.72)	0.683	1.17 (0.72, 1.90)	0.532
**Education**					
No formal education	39/88 (44.3)	*Reference*		*Reference*	
Primary	56/97 (57.7)	1.72 (0.96, 3.07)	0.069	1.50 (0.80, 2.81)	0.210
Secondary	156/366 (42.6)	0.93 (0.58, 1.49)	0.773	0.93 (0.54, 1.59)	0.789
Tertiary	62/124 (50.0)	1.26 (0.73, 2.17)	0.415	1.53 (0.77, 3.04)	0.227
**Monthly family income**					
<RM 4000	210/457 (46.0)	*Reference*		*Reference*	
RM 4000-10,000	67/140 (47.9)	1.08 (0.74, 1.58)	0.693	1.15 (0.74, 1.79)	0.528
RM >10,000	19/45 (42.2)	0.86 (0.46, 1.60)	0.632	0.84 (0.42, 1.71)	0.636
**BC history**					
No	307/660 (46.5)	*Reference*		*Reference*	
Yes	6/16 (37.5)	0.69 (0.25, 1.92)	0.477	0.54 (0.18, 1.65)	0.281
**BC screening history**					
No	237/510 (46.5)	*Reference*		*Reference*	
Yes	75/163 (46.0)	0.98 (0.69, 1.40)	0.919	0.83 (0.56, 1.24)	0.358
**TV ad recognition**					
No	155/347 (44.7)	*Reference*		*Reference*	
Yes	154/324 (47.5)	1.12 (0.83, 1.52)	0.457	1.22 (0.85, 1.77)	0.281
**Radio ad recognition**					
No	240/511 (47.0)	*Reference*		*Reference*	
Yes	68/153 (44.4)	0.90 (0.63, 1.30)	0.583	0.79 (0.51, 1.22)	0.289
**Print ad recognition**					
No	221/468 (47.2)	*Reference*		*Reference*	
Yes	88/196 (44.9)	0.91 (0.65, 1.27)	0.584	0.96 (0.66, 1.40)	0.847

[a]Adjusted for age, ethnicity, marital status, education, monthly family income, BC history, BC screening history, TV ad recognition, Radio ad recognition, Print at recognition

For peer review only

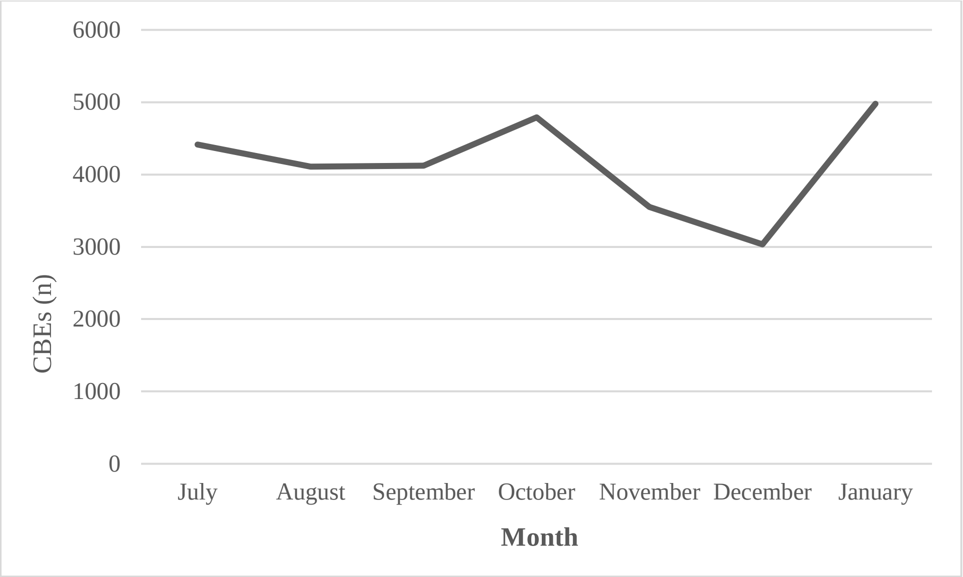

Figure 1 Clinical Breast Examinations (CBEs) undertaken in four public clinics (Petaling district) between July 2018 and January 2019

For peer review only

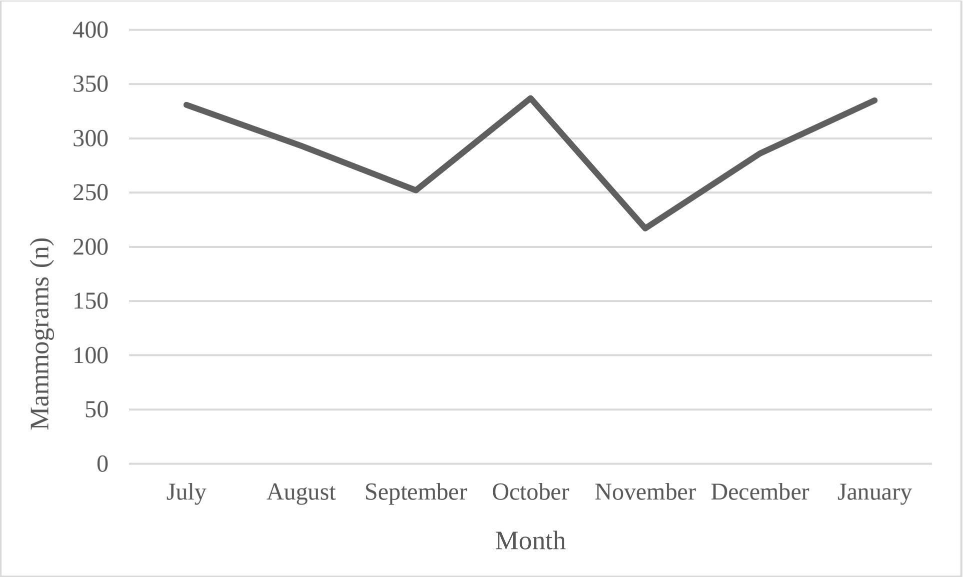

Figure 2 Mammograms undertaken in two public hospitals (Petaling district) between July 2018 and January 2019

For peer review only

Appendix Table 1 Information about all campaign activities and media used during BCAC-BC

**Time period** (2018)	**Media (Language)**	**Content**	**Implementation area**
**TV**			
24th September – 21st October<br>4 weeks<br>4-6 ads/ day	Advertisement, 30 seconds on TV3 (BM), TV9 (BM), 8TV (MAN) and YouTube (BM, MAN, ENG)	Cancer survivor highlighting the signs and symptoms she experienced, promoting early detection can save lives, highlights the importance of family support	Nationwide
**Radio**			
15th – 28th October<br>2 weeks<br>5-7 ads/ day	Advertisement, 30 seconds on I) Thr Raaga (TAM) and II) Lite FM (ENG)	Cancer survivors highlighting BC signs and symptoms, BC can be cured, highlighting that early detection can save lives and to see doctor urgently if symptoms are noticed, 9 out of 10 women survive BC if found and treated early, highlighting website and helpline	I) Peninsular Malaysia<br>II) Central Malaysia (including Petaling Jaya)
**Print materials**			
24th September – 28th October<br>5 weeks	Billboards (x7) (BM)	Displayed three BC survivors from each main ethnicity as well as Amber Chia (Malaysian supermodel) and the headline 'Don't be shy to check your breasts' and the slogan '9 out of 10 women can survive BC if found and treated early', information about the website and NCSM helpline	Petaling Jaya [a]
	Street buntings (x400) (BM)	*See billboards*	Petaling Jaya [a]
	Posters (BM and ENG)	*See billboards* + signs and symptoms of BC, see a doctor urgently if symptoms are noticed, early detection can save lives, BC can be cured	Clinics (A2 size x53) in Petaling Jaya [a]<br>Tropicana Shopping Mall (A1 size x 8)
	Roll-up banners	*See billboards*	Supermarkets (x5) in Petaling Jaya [a]
	Brochures (BM, ENG, MAN and TAM)	*See billboards* + signs and symptoms of BC, Breast-self-examination guide, early detection can save lives, BC survivor story	Clinics (x53) in Petaling Jaya [a]

For peer review only

8th October (x2), 14th October (x2), 28th October (x1)	5x Newspaper articles (BM and ENG), i.e. MY Metro Online, Harian Metro, The Star Online, Sunday Star, Berita Harian	*Key messages same as posters*	Respective areas of coverage
**Social media**			
24th September – 28th October<br>5 weeks	32 posts, including 11 boosted posts (BM, ENG)<br>2 x posts from Amber Chia (Malaysian supermodel)	What is BC, BCAC-BC campaign launch, signs and symptoms, breast self-examination, risk factors, prevalence of BC in Malaysia, importance of male support, prevention, campaign video, cancer survivor stories (x3), call to action, screening and diagnosis, early detection saves lives, quiz	Nationwide
**Website**			
	Link: www.becanceralert.com (BM and ENG)	Information about cancer in general, signs and symptoms, risk factors and early detection as well as access to all BCAC-BC materials, cancer survivor stories	Nationwide
**Launch event**			
27th September	1 hour launch event (ENG)	Stakeholders and media were invited to officially launch the BCAC-BC	University of Malaya, Kuala Lumpur

[a] Petaling Jaya was the study area for BCAC-BC

Ads – advertisement, BCAC – Be Cancer Alert Campaign, BM – Bahasa Melayu, BC – breast cancer, ENG- English, MAN – Mandarin, NCSM – National Cancer Society Malaysia, TAM- Tamil

For peer review only

Appendix Table 2 Change in average prompted knowledge score

	Pre Mean (SD)	Post Mean (SD)	Change Mean (SD)	P-value *(paired sample t-test)*	P-value *(independent sample t-test, equal variances assumed)*
All participants	7.45 (3.05)	7.84 (2.86)	0.39 (3.53)	0.005	-
BC history (n=16)	8.00 (2.99)	8.06 (2.91)	0.06 (3.26)	0.940	*Comparing BC history to no history* 0.711
No BC history (n=660)	7.44 (3.06)	7.83 (2.86)	0.39 (3.54)	0.004	
BC screening history (n=175)	7.65 (3.20)	8.31 (2.85)	0.66 (3.39)	0.011	*Comparing BC screening history to no screening history* 0.251
No BC screening history (n=498)	7.38 (3.00)	7.69 (2.83)	0.31 (3.54)	0.052	
BCAC recognisers (n=441)	7.68 (2.89)	8.26 (2.46)	0.59 (3.29)	<0.001	*Comparing BCAC recognisers to non-recognisers (equal variances not assumed)* 0.0501
BCAC non-recognisers (n=211)	6.98 (3.29)	6.96 (3.32)	-0.24 (3.88)	0.929	

Appendix Table 3 Number of CBEs (n=29,000) and mammograms (n=2,051) undertaken by ethnicity and age (July 2018-January 2019)

	**CBE** *n* (%)	**Mammogram**[a] *n* (%)
**Ethnicity**		
Malay	24 532 (84.6)	1152 (56.2)
Chinese	2 434 (8.4)	551 (26.9)
Indians	1 516 (5.2)	331 (16.1)
Others	518 (1.8)	17 (0.8)
**Age group**		
20-29	16 687 (57.5)	1 (0.1)
30-39	10 722 (37.0)	14 (1.6)
40-49	1 283 (4.4)	332 (37.0)
50-59	265 (0.9)	333 (37.1)
>60	43 (0.1)	-
60-69	-	172 (19.2)
>70	-	46 (5.1)

CBE – clinical breast examination

[a] Mammogram data on age is from hospital Sg Buloh only (n=898)

For peer review only

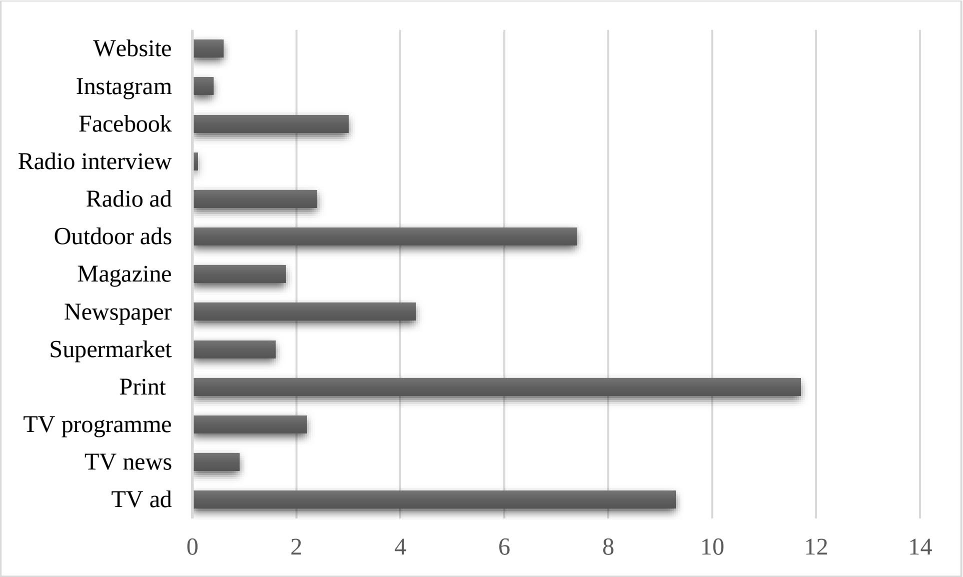

Appendix Figure 1 Advertisement channels through which participants noticed the BCAC-BC advertisements (unprompted)

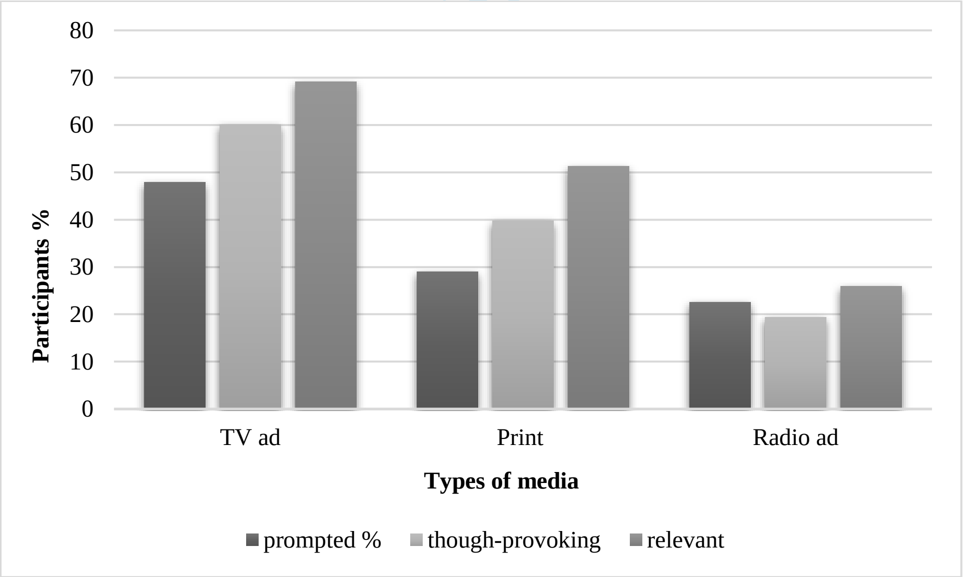

Appendix Figure 2 Advertisement channels through which participants noticed the BCAC-BC advertisements (prompted) and views on materials

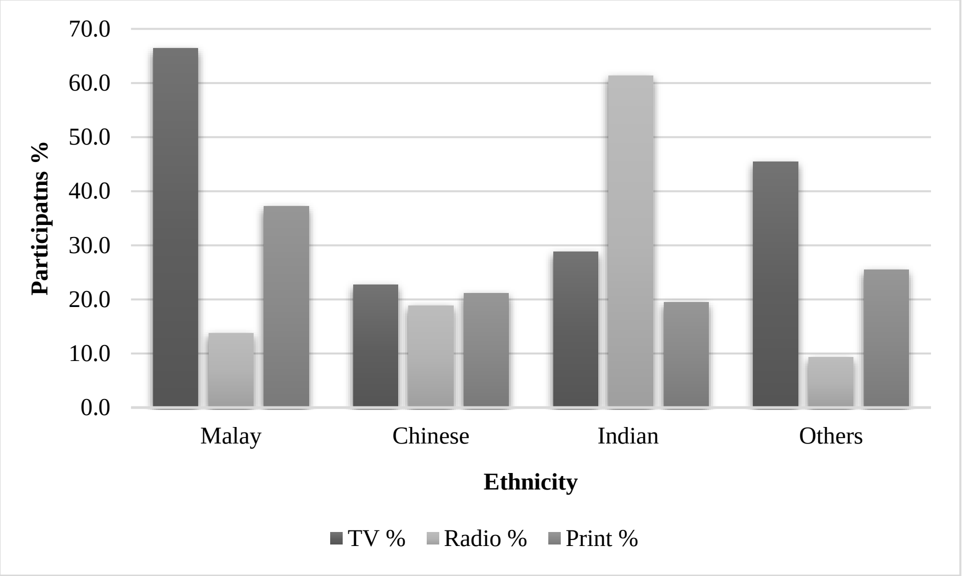

Appendix Figure 3 Difference in campaign material reach between ethnicities

For peer review only

STROBE 2007 (v4) checklist of items to be included in reports of observational studies in epidemiology*
Checklist for cohort, case-control, and cross-sectional studies (combined)

Section/Topic	Item #	Recommendation	Reported on page #
**Title and abstract**	1	(*a*) Indicate the study's design with a commonly used term in the title or the abstract	2
		(*b*) Provide in the abstract an informative and balanced summary of what was done and what was found	2
**Introduction**			
Background/rationale	2	Explain the scientific background and rationale for the investigation being reported	4
Objectives	3	State specific objectives, including any pre-specified hypotheses	4
**Methods**			
Study design	4	Present key elements of study design early in the paper	4,5
Setting	5	Describe the setting, locations, and relevant dates, including periods of recruitment, exposure, follow-up, and data collection	5
Participants	6	(*a*) *Cohort study*—Give the eligibility criteria, and the sources and methods of selection of participants. Describe methods of follow-up<br>*Case-control study*—Give the eligibility criteria, and the sources and methods of case ascertainment and control selection. Give the rationale for the choice of cases and controls<br>*Cross-sectional study*—Give the eligibility criteria, and the sources and methods of selection of participants	4,5
		(*b*) *Cohort study*—For matched studies, give matching criteria and number of exposed and unexposed<br>*Case-control study*—For matched studies, give matching criteria and the number of controls per case	
Variables	7	Clearly define all outcomes, exposures, predictors, potential confounders, and effect modifiers. Give diagnostic criteria, if applicable	5
Data sources/ measurement	8*	For each variable of interest, give sources of data and details of methods of assessment (measurement). Describe comparability of assessment methods if there is more than one group	5
Bias	9	Describe any efforts to address potential sources of bias	5,6
Study size	10	Explain how the study size was arrived at	6
Quantitative variables	11	Explain how quantitative variables were handled in the analyses. If applicable, describe which groupings were chosen and why	6
Statistical methods	12	(*a*) Describe all statistical methods, including those used to control for confounding	6
		(*b*) Describe any methods used to examine subgroups and interactions	6
		(*c*) Explain how missing data were addressed	6
		(*d*) *Cohort study*—If applicable, explain how loss to follow-up was addressed<br>*Case-control study*—If applicable, explain how matching of cases and controls was addressed	

For peer review only

		Cross-sectional study—If applicable, describe analytical methods taking account of sampling strategy	
		(*e*) Describe any sensitivity analyses	
**Results**			
Participants	13*	(a) Report numbers of individuals at each stage of study—eg numbers potentially eligible, examined for eligibility, confirmed eligible, included in the study, completing follow-up, and analysed	7
		(b) Give reasons for non-participation at each stage	7
		(c) Consider use of a flow diagram	
Descriptive data	14*	(a) Give characteristics of study participants (eg demographic, clinical, social) and information on exposures and potential confounders	7
		(b) Indicate number of participants with missing data for each variable of interest	19,20
		(c) *Cohort study*—Summarise follow-up time (eg, average and total amount)	
Outcome data	15*	*Cohort study*—Report numbers of outcome events or summary measures over time	
		Case-control study—Report numbers in each exposure category, or summary measures of exposure	
		Cross-sectional study—Report numbers of outcome events or summary measures	7,8
Main results	16	(*a*) Give unadjusted estimates and, if applicable, confounder-adjusted estimates and their precision (eg, 95% confidence interval). Make clear which confounders were adjusted for and why they were included	8-10, 20,22
		(*b*) Report category boundaries when continuous variables were categorized	19
		(*c*) If relevant, consider translating estimates of relative risk into absolute risk for a meaningful time period	
Other analyses	17	Report other analyses done—eg analyses of subgroups and interactions, and sensitivity analyses	7-10, 20-23
**Discussion**			
Key results	18	Summarise key results with reference to study objectives	9,10
Limitations	19	Discuss limitations of the study, taking into account sources of potential bias or imprecision. Discuss both direction and magnitude of any potential bias	10,11
Interpretation	20	Give a cautious overall interpretation of results considering objectives, limitations, multiplicity of analyses, results from similar studies, and other relevant evidence	9-11
Generalisability	21	Discuss the generalisability (external validity) of the study results	11
**Other information**			
Funding	22	Give the source of funding and the role of the funders for the present study and, if applicable, for the original study on which the present article is based	12

*Give information separately for cases and controls in case-control studies and, if applicable, for exposed and unexposed groups in cohort and cross-sectional studies.
Note: An Explanation and Elaboration article discusses each checklist item and gives methodological background and published examples of transparent reporting. The STROBE checklist is best used in conjunction with this article (freely available on the Web sites of PLoS Medicine at http://www.plosmedicine.org/, Annals of Internal Medicine at http://www.annals.org/, and Epidemiology at http://www.epidem.com/). Information on the STROBE Initiative is available at www.strobe-statement.org.

For peer review only

BMJ Open

The impact of a mass media campaign on breast cancer symptoms awareness and screening uptake in Malaysia: findings from a quasi-experimental study

Journal:	*BMJ Open*
Manuscript ID	bmjopen-2019-036503.R2
Article Type:	Original research
Date Submitted by the Author:	23-Jun-2020
Complete List of Authors:	Schliemann, Desiree ; Queen's University Belfast, Centre for Public Health<br>Htay , Mila; University of Malaya, Department of Social and Preventive Medicine<br>Dahlui, Maznah; University of Malaya Faculty of Medicine, Department of Social and Preventive Medicine<br>Paramasivam, Darishiani; University of Malaya Faculty of Medicine, Department of Social and Preventive Medicine<br>Cardwell, Christopher; Queen's University Belfast Centre for Public Health, School of Medicine, Dentistry and Biomedical Sciences<br>Ibrahim Tamin, Nor Saleha; Ministry of Health Malaysia Disease Control Division<br>Somasundaram, Saunthari ; National Cancer Society Malaysia<br>Donnelly, Conan; National Cancer Registry<br>Su, Tin ; Monash University - Malaysia Campus, Jeffrey Cheah School of Medicine and Health Sciences<br>Donnelly, Michael; Queen's University Belfast Centre for Public Health
**Primary Subject Heading**:	Public health
Secondary Subject Heading:	Communication, Global health, Oncology
Keywords:	PUBLIC HEALTH, Breast tumours < ONCOLOGY, EDUCATION & TRAINING (see Medical Education & Training)

SCHOLARONE™
Manuscripts

I, the Submitting Author has the right to grant and does grant on behalf of all authors of the Work (as defined in the below author licence), an exclusive licence and/or a non-exclusive licence for contributions from authors who are: i) UK Crown employees; ii) where BMJ has agreed a CC-BY licence shall apply, and/or iii) in accordance with the terms applicable for US Federal Government officers or employees acting as part of their official duties; on a worldwide, perpetual, irrevocable, royalty-free basis to BMJ Publishing Group Ltd ("BMJ") its licensees and where the relevant Journal is co-owned by BMJ to the co-owners of the Journal, to publish the Work in this journal and any other BMJ products and to exploit all rights, as set out in our licence.

The Submitting Author accepts and understands that any supply made under these terms is made by BMJ to the Submitting Author unless you are acting as an employee on behalf of your employer or a postgraduate student of an affiliated institution which is paying any applicable article publishing charge ("APC") for Open Access articles. Where the Submitting Author wishes to make the Work available on an Open Access basis (and intends to pay the relevant APC), the terms of reuse of such Open Access shall be governed by a Creative Commons licence – details of these licences and which Creative Commons licence will apply to this Work are set out in our licence referred to above.

Other than as permitted in any relevant BMJ Author's Self Archiving Policies, I confirm this Work has not been accepted for publication elsewhere, is not being considered for publication elsewhere and does not duplicate material already published. I confirm all authors consent to publication of this Work and authorise the granting of this licence.

For peer review only

Title: The impact of a mass media campaign on breast cancer symptoms awareness and screening uptake in Malaysia: findings from a quasi-experimental study

Authors: Désirée Schliemann[1] (PhD); Mila Nu Nu Htay[2,3] (MMedSc); Maznah Dahlui[2,4] (PhD); Darishiani Paramasivam[2] (MSc), Christopher R. Cardwell[1] (PhD); Nor Saleha Binti Ibrahim Tamin[5] (MPH); Saunthari Somasundaram[6] (MBA), Conan Donnelly[7] (PhD); Tin Tin Su[2,8] (PhD); Michael Donnelly[1] (PhD)

[1] Centre for Public Health and UKCRC Centre of Excellence for Public Health, Queen's University Belfast, Belfast, UK

[2] Centre for Population Health (CePH), Department of Social and Preventive Medicine, University of Malaya, Kuala Lumpur, Malaysia

[3] Department of Community Medicine, Melaka-Manipal Medical College, Manipal Academy of Higher Education (MAHE), Melaka, Malaysia

[4] Facultas Public Health, University Airlangga, Indonesia

[5] Ministry of Health Malaysia, Kuala Lumpur, Malaysia

[6] National Cancer Society Malaysia, Kuala Lumpur, Malaysia

[7] National Cancer Registry, Cork, Ireland

[8] South East Asia Community Observatory (SEACO), Jeffrey Cheah School of Medicine and Health Sciences, Monash University Malaysia

Word count main text: 3451

Corresponding author: Désirée Schliemann / email: d.schliemann@qub.ac.uk/ phone: +44 28 9097 6350/ fax: +44 28 90235900 / ORCID ID: 0000-0002-8746-3002

For peer review only - http://bmjopen.bmj.com/site/about/guidelines.xhtml

Abstract

Objective: To evaluate the impact of a mass media campaign in terms of improving breast cancer (BC) symptoms awareness and screening uptake.

Design: Before- and after- study with comparator groups.

Setting: Selangor State, Malaysia.

Participants: Malaysian women aged ≥40 years (n=676) from randomly selected households.

Intervention: A culturally adapted mass media campaign (TV, Radio, print media and social media).

Primary and secondary outcome measures: The primary endpoint was BC symptoms awareness, which was assessed with the *Breast Cancer Awareness Measure* pre- and post-campaign. Secondary outcomes included campaign reach, self-efficacy to notice BC symptoms and clinical outcomes. Clinical breast examination and mammogram screening data were collected from hospitals and clinics.

Results: Most participants recognised at least one of the campaign materials (65.2%). The odds of seeing the campaign were lowest for Chinese women (adjusted OR 0.69, 95% CI 0.42, 1.14) compared to Malays and for women aged ≥70 years (adjusted OR 0.47, 95% CI 0.23, 0.94) compared to younger women. Participants who recognised the campaign were significantly more likely to have improved awareness post-campaign compared to non-recognisers particularly for key symptoms such as 'a lump or thickening in your breast' (88.9% vs 62.1%) and 'discharge or bleeding from nipple' (79.7% vs 55.3%). Improvement in symptoms awareness scores was not associated with socio-demographic variables.

Conclusions: Implementation in Malaysia of an evidence-based mass media campaign from the UK that was culturally adapted appeared to lead to improved awareness about some BC symptoms, though various modes of media communication and perhaps other health education approaches may be required to extend the reach to diverse, multi-ethnic populations and all age groups.

Key words: public health, health promotion, breast cancer, awareness, symptoms, screening, signs, clinical breast examination, mammogram, social media, media campaign.

For peer review only - http://bmjopen.bmj.com/site/about/guidelines.xhtml

Article Summary

Strengths and limitations

- The public health intervention was subjected to a systematic cultural adaptation process and drew upon best available evidence.
- This was the first study to evaluate a mass media campaign designed to improve breast cancer awareness in Malaysia.
- The study outcomes were assessed using psychometrically validated measures that were pilot-tested with the Malaysian multi-ethnic population.
- The development and evaluation approach provides a blueprint for public health researchers in other Asian countries.
- The nationwide distribution of the mass media campaign meant that it was not possible to have conventional control groups though the study was able to create internal comparator groups of 'recognisers' vs 'non-recognisers'.

For peer review only

Introduction

Breast cancer (BC) comprises 17.7% of all cancers in Malaysia[1] and it is the commonest cause of cancer deaths among women in Malaysia[2]. The high cancer mortality rate is due to several factors particularly late detection. For example, between 2007–2011, 43% of women were diagnosed with BC at a late stage (stage 3 and 4)[1] compared to <20% of women in the UK[3], supporting the benefits of early detection interventions that are more commonly implemented in high income countries[4]. Late presentation is due, at least partly, to low cancer awareness. Research indicates that there is a lack of awareness among Malaysian women about common symptoms of BC[5,6], e.g. only 34% of women recognised 'a painless breast lump' as a BC symptom[6]. Other causes of delayed detection and diagnosis include denial, lack of knowledge, negative perceptions of the disease, over-reliance on traditional medicine, misperceived risk, emotional barriers and negative perceptions towards screening[7–9]. Furthermore, current screening guidelines encourage opportunistic biennially screening for BC in Malaysian females aged between 50-74 years[10] and population-based screening is lacking. Nationwide education programmes are encouraged as a first step to improve early detection of BC[11] in settings where population-based cancer screening is unavailable and individuals are required to self-initiate help-seeking when symptoms are experienced. However, robust evaluations of mass media interventions for cancer health promotion in Asia, and particularly Malaysia, are lacking[12]. In high income countries, mass media campaigns have improved symptoms awareness[13,14] and increased the number of BC referrals[15]. Industry and non-governmental organisations (NGOs) exert mass media type efforts regularly to raise awareness about BC but these interventions tend to be short-lived and are not subjected to robust evaluation[16]. In response to this knowledge gap, our collaboration developed a culturally acceptable, evidenced-based mass media campaign for Malaysia - the *Be Cancer Alert Campaign* (BCAC)[17,18]. The primary endpoint of this study was BC symptoms awareness. Secondary outcomes included the reach of the campaign, perceived self-efficacy to detect symptoms, visits to a health care professional to discuss BC symptoms, number of BC screenings undertaken (clinical breast examination (CBE) and mammogram) and the number of BC cases diagnosed.

Methods

The protocol for the evaluation of the BCAC-BC was published previously[17] and is explained here in brief. The Research Ethics Committee, University of Malaya (ID: 2016126–4668) and the National Medical Research Register (ID: NMRR-18-1961-42562), granted ethical approval.

Study population and sampling

For peer review only - http://bmjopen.bmj.com/site/about/guidelines.xhtml

Malaysia comprises three main ethnicities: Malay (69%), Chinese (23%) and Indian (7%)[19]. The sample was drawn from Petaling District (Sungai Buloh and Petaling Jaya), Selangor State, with a multi-ethnic population composition[17]. Trained interviewers visited randomly selected households and invited female residents to participate if they were aged ≥40 years, spoke English or Malay, were able to provide answers without support from others and provided informed consent. Participants were interviewed one-to-twelve weeks before (i.e. July to September 2018) and after (i.e. November 2018 to January 2019) the BCAC-BC implementation.

Intervention

Implementation of the BCAC-BC mass media campaign occurred over a 5-week period (September-October 2018). Appendix Table 1 provides a description of campaign materials**.** Nationwide TV and radio presented advertisements; the study area received print materials (i.e. billboards, street bunting, posters and brochures); the National Cancer Society Malaysia (NCSM) delivered a social media campaign via their Facebook page. All materials contained a link to a bespoke BCAC website and highlighted the NCSM helpline.

Patient and public involvement

This study involved the leading advocacy organisation for cancer prevention and cancer care in Malaysia – NCSM. Representatives from NCSM, the Ministry of Health (MoH), Malaysia and the university researchers worked in partnership to address the research questions. In particular, we involved our partners, members of the public and cancer survivors in focus groups and interviews regarding the preparation of the campaign materials and their delivery and we included cancer survivors in the TV advertisements. Finally, the results of the research were disseminated in collaboration with the NCSM, MoH and cancer survivors.

Data collection

Questionnaire

Pre- and post-campaign surveys were conducted to evaluate the impact of the mass media campaign on BC awareness with questions informed by the *Breast Cancer Awareness Measure* (CAM)[20]. We assessed *Unprompted* BC symptoms knowledge by asking, 'There are many warning signs and symptoms of BC. Please name as many as you can think of'. We assessed *Prompted* knowledge by asking, 'Do you think [symptom] could be a sign for BC?' A total score for unprompted knowledge and

For peer review only - http://bmjopen.bmj.com/site/about/guidelines.xhtml

prompted knowledge, respectively, was calculated by summing the correct answers. Socio-demographic characteristics, cancer history (of respondent) and monthly household income were assessed pre-campaign only. Post-campaign, specific questions assessed campaign reach[21]. Participants were asked whether they noticed the BCAC logo and other materials. The final set of questions asked participants whether or not they found the materials relevant and acceptable; shared/discussed the campaign information and whether or not they or their family and/or friends visited a health professional as a result of seeing the BCAC-BC.

Social media monitoring

The social media activity was monitored daily by an external agency; and it was evaluated in terms of post reach (total number of unique users who saw the advertisement/post on their Facebook feed), interaction (total number of emoji reactions including like, love, smile, wow, sad and angry), amplification (number of shares per post), conversation (number of comments per post) and total engagement (total number of interactions, amplification and conversation per post).

Helpline

Trained nurses recorded (with consent) NCSM Helpline callers who said that they got the helpline number from one of the BCAC campaign materials in terms of date of call, gender of caller, reason for calling and campaign source for the number.

Health service use

Staff from local health clinics and hospitals recorded the number of CBE and mammograms undertaken between July 2018 and January 2019 as well as basic socio-demographic information.

Sample size

It was estimated that 550 participants would provide 80% power to detect, as statistically significant at a 5% level, an increase by 6% in the proportion of individuals who were aware that an unexplained lump or swelling was a symptom of BC[17] using a two-sided McNemar Test.

Data analysis

For peer review only - http://bmjopen.bmj.com/site/about/guidelines.xhtml

The McNemar test assessed pre- and post-campaign proportional differences in knowledge/awareness. Chi-square tests examined associations between recognition of one or more BCAC materials when prompted (i.e. 'BCAC-recognisers' and 'non-recognisers') and BC awareness; and tested associations between BC history or BC screening history and BC symptoms awareness. Logistic regression investigated the relationship between BCAC recognition (yes versus no) and potential explanatory variables. The final model from which adjusted estimates were calculated contained age, gender, ethnicity, marital status, education, monthly family household income, BC history and BC screening history (received BC screening – either CBE or mammogram- in the past two years). Similar models were applied to the outcome, 'knowledge improved' (yes vs no). Logistic regression analyses were repeated using robust standard errors to adjust for potential clustering within households[22] (the results were similar to the results that are presented here). Service utilisation data were charted over the relevant time-periods. All available information was included in the analysis.

Results

The BCAC-BC was implemented as planned (except that the TV advertisement was conducted for four weeks instead of five weeks (Appendix Table 1).

Study population

A total of 992 participants completed the pre-campaign survey and 68% (676/992) completed the post-campaign survey (**Table 1**). Participants who did not complete the follow-up survey could not be reached or refused to participate post-campaign. Forty-one percent of participants who completed both surveys were aged between 40 and 49 years and about 30% of women were aged between 50-59 years. Malays were the most commonly represented ethnic group (51.6 %), followed by Chinese (22.3%) and Indians (17.8%). Most women were married (86.8%); just over half completed secondary education (54.2%); and most (71%) had a monthly low household income of less than RM 4,000[23]. Sixteen women (2.4%) had a personal history of BC and 25.9% underwent a mammogram in the previous two years. Women who completed the follow-up assessment were significantly younger compared to women who completed only the pre-campaign survey. There was a higher proportion of Malay and Indian participants and fewer Chinese participants at follow-up compared to baseline (**Table 1**).

Campaign reach

Eighteen percent of participants reported that they previously saw the BCAC logo. Participants remembered (unprompted) seeing BCAC posters in clinics (11.7%), TV advertisements (9.3%), outdoor

For peer review only - http://bmjopen.bmj.com/site/about/guidelines.xhtml

materials (bunting/billboards, 7.4%), newspaper articles (4.3%) and hearing BCAC radio advertisements (2.4%) (Appendix Figure 1). Furthermore, 26.9% recognised the slogan, '*Don't be shy to check your breast*'. When participants were prompted or shown BCAC materials, 65.2% recognised BCAC materials (i.e. 47.9% recognised the TV advertisements, 29% recognised print materials and 22.6% recognised the radio advertisement). TV advertisements were deemed thought-provoking by 60% and relevant by 69% (Appendix Figure 2). Print materials were thought-provoking for 40% and relevant for 51.3%; fewer participants described radio ads in these terms (19.4% and 25.9%). Almost 40% discussed the advertisements with friends/family and 21.7% stated that they or their family/friends went to see a doctor as a result of seeing the advertisements. TV advertisements were most commonly recognised by Malays (66.4%) followed by 'others' (45.5%). Indian participants (61.3%) recognised radio advertisements more often than other ethnic groups. Print advertisements were recognised by more Malays (37%) compared to between 19.5% and 25.5% of other ethnicities (Appendix Figure 3). Between-ethnic groups differences in campaign reach were confirmed by regression analysis (**Table 2**). The odds that participants saw one or more of the BCAC materials (when prompted) were significantly lower for Chinese and 'others' compared to Malays (adjusted OR 0.25, 95% CI 0.15; 0.40, $p<0.001$ and OR 0.34, 95% CI 0.18; 0.65, $p=0.001$). Campaign reach towards people aged $\geq$70 years appeared to be relatively poor (adjusted OR 0.47, 95% CI 0.23; 0.94, $p=0.032$).

Thirty-two social media 'posts' were shared through Facebook and 11/32 posts were boosted. The boosted posts reached significantly more people than 'ordinary' posts. The boosted post describing BC symptoms in Malay achieved the highest reach (reach: 202,430; total engagement: 4,498 and shares: 1,379) compared to the same post in English (reach: 18,012, total engagement: 126 and shares: 25). The second most popular post showed highlights of the campaign launch (reach: 19,071, total engagement: 816 and shares: 45) and a post on breast self-examination in English (reach: 15,673, total engagement: 322 and shares: 87). Posts that were not boosted reached between 1,495 and 486 Facebook users whereas posts that were boosted reached between 202,430 and 3,412 users.

The helpline received five calls from women (aged 22-42 years old) who called after seeing a BCAC advertisement; 4/5 women reported experiencing potential BC symptoms and one woman had had a negative biopsy result and enquired about follow-up appointments.

Campaign impact

Knowledge improved significantly for six BC symptoms (unprompted): 'Pain in one of your breasts or armpits' (18.9% vs 23.2%), 'discharge or bleeding from nipple' (10.1% vs 16.4%), 'nipple rash' (1.3% vs 3.6%), 'redness of your breast skin' (1.5% vs 7.2%), 'lump or thickening under armpit' (3.4% vs 7.5%) and 'changes in the shape of your breast or nipple' (1.5% vs 3.7%) significantly at follow-up (**Table 3**). Knowledge/awareness (prompted) increased significantly for three BC symptoms: 'change

For peer review only - http://bmjopen.bmj.com/site/about/guidelines.xhtml

in the position of your nipple' (58.7% vs 67.3%), 'pain in one of your breasts or armpits' (72.5% vs 77.5%) and 'redness of your breast skin' (54.9% vs 62.4%). Overall symptom knowledge/awareness scores (prompted) improved significantly at follow up (pre-Mean 7.45 (SD: 3.05) and post-Mean 7.84 (SD: 2.86)). BCAC recognisers identified on average a higher number of BC symptoms at follow-up compared to baseline (pre-Mean 7.68 (SD: 2.89) and post-Mean 8.26 (SD: 2.46)). There was some evidence that the improvement in awareness by BCAC recognisers was higher compared to non-recognisers (Mean change 0.59 (SD: 3.29) vs Mean change -0.24 (SD: 3.88)). **(**Appendix Table 2**).**

A significantly higher proportion of BCAC recognisers compared to non-recognisers who were not aware of BC symptoms at baseline, improved their knowledge (unprompted) about the symptom 'a lump or thickening in your breast' at follow-up (55.9% vs 41.0%) (**Table 3**). Furthermore, significantly more BCAC recognisers improved their awareness (prompted) about the following symptoms 'change in the position of your nipple' (62.7% vs 41.6%), 'discharge or bleeding from nipple' (79.7% vs 55.3%), 'a lump or thickening in your breast' (88.9% vs 62.1%) and 'changes in the size of your breast or nipple' (67.2% vs 46.2%) compared to non-recognisers. The proportion of women who were very/fairly confident that they would recognise a BC symptom increased at follow-up (58.9% vs 68.9%) (**Table 3**) and there was no significant difference between BCAC recognisers and non-recognisers (63.7% vs 54.4%). Pre-campaign, 96.1% of women reported that they would see their doctor within two weeks if they noticed a BC symptom and there was no change at follow-up. Regression analysis indicated that none of the socio-demographic or campaign recognition variables exerted a marked influence on BC symptoms awareness post-campaign after adjustment (**Table 4**).

From July 2018 to January 2019, 29,000 CBEs were performed (**Figure 1**) as well as 2,051 mammograms (**Figure 2**). More mammograms and CBEs were conducted in October (337 and 47,892), January (335 and 4,978) and July (331 and 4,415) compared to other months. Most CBEs were performed on Malay ethnic women (85%) (Appendix Table 3) and women aged 20-29 years (58%) and 30-39 years (37%). Mammograms were undertaken for Malays (56%), Chinese (27%) and Indians (16%).

Discussion

Research-informed BC mass media campaigns with robust evaluation are lacking in Asian countries like Malaysia[16]. The need to improve BC awareness was confirmed by the limited symptoms awareness and low confidence to notice symptoms that were identified in the pre-campaign survey results. The majority of women noticed the BCAC-BC materials, in particular, the TV advertisements (even though numerous BC awareness activities took place in October as part of BC awareness month); and they found the culturally adapted campaign materials acceptable and relevant to their life circumstances. The salience of TV advertisements as the most commonly recognised campaign material is in keeping with

For peer review only - http://bmjopen.bmj.com/site/about/guidelines.xhtml

For peer review only

other robust evaluations[13,24]. Furthermore, awareness about individual BC symptoms was higher amongst BCAC-recognisers compared to non-recognisers. The high reach and improvement in symptoms awareness suggested that the campaign had an impact in terms of improving awareness about some BC symptoms.

Campaign reach varied between ethnicities, though improved awareness of BC symptoms did not appear to vary across socio-demographic variables including ethnicity. The differential reach that was experienced by ethnic groups was similar to the findings from the Be Cancer Alert Campaign for Colorectal Cancer (BCAC-CRC) in Malaysia[25]. Different attitudes and behaviours across ethnic groups regarding help-seeking may help to explain the apparent discrepancy. For example, USA health survey participants who did not seek health information in the media and preferred to trust their health service provider tended to be older and have a lower socio-economic status[26]. Mass media as a public health programme or intervention requires careful tailoring to maximise the reach and impact of given public health messages.

Although the BCAC-BC had greater reach compared to the BCAC-CRC, the BCAC-CRC appeared to be more successful in terms of improving symptoms awareness[25], perhaps, because of the higher pre-campaign symptom knowledge/awareness (prompted and unprompted) among the BCAC-BC sample compared to BCAC-CRC participants. Awareness improved significantly at follow-up for three prompted BC symptoms, whereas awareness about all prompted CRC symptoms improved. The success of the BCAC-BC was due partly to the systematic cultural adaptation process[18] that was undertaken in order to address, for example, the need to refer to breast-related issues with contextual sensitivity and to include only information about a lump. Although a lump appeared already to be a commonly known symptom, it was unacceptable culturally to present other symptoms of BC. Such restrictions did not apply to the BCAC-CRC.

BC is the best-known cancer site in most countries. It was the most common cancer reported in major online newspapers in the USA[27] and it is the most 'promoted' cancer in Malaysia[16]. Also, online information-seeking behaviour related to BC is higher during October (BC awareness month)[28,29]. Findings from the BCAC social media campaigns demonstrated higher reach and engagement for the BC social media posts compared to the CRC posts (i.e. highest reach of CRC-related post: 92,678 users vs BC-related post: 202,430 users; highest engagement with CRC-related post: 2,065 users vs BC-related post: 4,498 users)[25]. CRC campaign material about symptoms may be perceived to be less pleasant to share compared to BC symptoms. Overall, it may not be surprising that BC awareness levels are higher than other cancer sites such as CRC and that the gains from a mass media campaign directed towards BC would be smaller given this elevated baseline awareness. It might be more impactful to reserve the use of mass media campaigns for areas where there is a known low level of awareness and knowledge. The number of calls reported by NCSM as a result of both BCAC campaigns was low

For peer review only - http://bmjopen.bmj.com/site/about/guidelines.xhtml

compared to caller volume reported in other campaign evaluations, e.g. a print and online media campaign targeting low-income African American women for 2.5 months received 97 calls[30].

The apparently high number of CBEs was encouraging and may suggest good implementation of CBE guidelines among younger women (i.e. CBE every three years) but not regarding women aged ≥40 years (5.4%) (i.e. an annual CBE)[31]. Previous research on CBE uptake in Malaysia found similar CBE rates across age groups[32]. The fact that data reflects public health clinic activity may explain why most CBEs were performed on Malay women (85%) - i.e. public health clinics were utilised mainly by Malays while Chinese tended to visit private clinics for screening activity though they may avail of public health care depending on costs). Mammogram activity in this study did not appear to be affected by ethnicity and were conducted among women aged ≥40 years (98.4%) which is in line with BC screening guidelines (i.e. mammograms biennially for women aged 50-74 years)[10]. Yet, the relatively low number of mammograms may highlight gaps in secondary BC prevention and care in Malaysia. Health service use findings from the *Be Clear on Cancer* campaign in England for women aged 70 years and older suggested that though screening referrals increased, the number of cancer cases detected did not increase thereby raising questions about the value of the campaign in the face of the higher workload and low conversion rate[15]. Findings from studies in the USA present mixed results about improved screening uptake. For example, one study found that screening activity in November (following BC awareness month) increased during the 90s when BC awareness and screening were low; however, increments in screening activity of this kind lessened with time as overall screening levels reached a high threshold[33]. The USA Health Communication Survey found that TV viewing (news or entertainment) prompted health information-seeking behaviour beyond socio-demographic variables and influenced cancer screening behaviour[34]. Data covered only seven months and CBE data and private clinic data were unavailable. Mindful of service data limitations, this is the first study to present CBE and mammogram activity over a series of consecutive months in Malaysia. The highest number of mammograms and CBEs were conducted in July, October and January. October is 'breast cancer awareness month' and June and December are holiday months. Although a public awareness BC campaign may increase screening rates, due to wide-spread routine screening activity throughout the year, campaigns may lose impact regarding the detection BC cases (i.e. people at low- or medium-risk and the 'worried well' may be more likely to respond to cancer awareness campaigns)[33,35]. BC campaign activities other than those related to the BCAC-BC may also have influenced participants' responses; and this potential limitation needs to be kept in mind when interpreting findings regarding the improvement of BC awareness. In addition, participants may have improved their knowledge due to the pre-campaign survey though the results from the comparative analysis of BCAC recognisers and non-recognisers did not support this interpretation.

A recent report[36] stated that evidence about the role and effectiveness of media channels in public health campaigns is limited due, partly and for obvious reasons, to a lack of controlled studies and randomised

For peer review only - http://bmjopen.bmj.com/site/about/guidelines.xhtml

control trials. There have been very few mass media campaigns in Asia that have been scientifically evaluated[12]. This study afforded a degree of control by creating and comparing internal groups based on whether or not they recognised the campaign. The suggestion that longer, more intensive campaigns would be more impactful[36,37] is a reasonable one. However, our evaluation demonstrated that even with a modest and short-term campaign, it is possible to improve knowledge about BC symptoms and influence help-seeking behaviour. Of course, the long-term impact of the campaign and sustainability of the improvements are unknown.

Conclusion

The BCAC-BC study addresses a knowledge gap and adds important insights into the impact of mass media for BC health promotion as well as implementation challenges related to (South East) Asia. Overall, the findings presented here favour positively the use of mass media in breast health promotion. Future campaigns may be enhanced via increased collaboration with disciplines such as social marketers and health journalism[38]. There appears to be a need for mass media campaigns to be tailored to particular sub-populations or hard-to-reach subgroups, especially in the context of multi-cultural societies. Women aged ≥70 years for example, were less likely to observe or notice the BCAC-BC materials and to have received a mammogram (5%). Targeting older women and women from ethnic minorities may be an important consideration in efforts to improve the reach of future BC awareness activities in Malaysia[15].

Declarations

Ethics approval and consent to participate

Ethics approval for the study was granted by the Medical Research Ethics Committee, University Malaya Medical Centre (ID: 2016126–4668) and by the National Medical Research Register (NMRR-18-1961-42562). Informed consent to participate was provided by all study participants. The study was performed in accordance with the Declaration of Helsinki.

Consent for publication

Consent for publication was signed by all study participants.

Data availability

For peer review only - http://bmjopen.bmj.com/site/about/guidelines.xhtml

For peer review only

Data can be made available upon reasonable request.

Conflict of interest

The authors declare no conflict of interest.

Funding

This study is funded by UK MRC-Newton Ungku Omar Funding (MR/P013910/1). The collaborative grant application was subjected to peer-review by individual academic reviewers and the final decision about funding was made by an expert panel. The funder had no role in the design of the study, collection, analysis, and interpretation of data or in writing the manuscript.

Contributorship statement

MDo and TTS conceptualised and planned the project and are the Co-PIs of the successful grant award from UK MRC-Newton Ungku Omar Fund. CD conducted the power calculation and provided guidance on the study design. DS, MNNH, DP, TTS and MDa planned and coordinated the study and data collection. SS guided the BCAC campaign design and implementation and NSBIT guided the collection of health service data. MNNH collected data from hospitals and clinics. DS and CC conducted the statistical analysis. DS drafted the manuscript. MDo led the editing and refinement of the manuscript. All authors contributed to, reviewed and approved the final manuscript.

Acknowledgements

We would like to acknowledge all involved in the development of the Be Cancer Alert Campaign materials and would like to acknowledge that the Be Cancer Alert Campaign materials were adapted from materials produced by the Public Health Agency, Northern Ireland for the Be Cancer Aware Campaign. We would also like to thank Assoc. Prof. Siew Yim Loh who assisted in the training of data collectors and the distribution of campaign materials. Furthermore, we thank the Department of Statistics Malaysia for providing the randomly selected households. We would like to acknowledge Datin Dr. Premila A/P Kanagasabai (Selayang hopsital)Dr. Yun Sii Ing (Sg Buloh hospital), Dr. Farah Nadrah Mohd Nasir (Sg Buloh Hospital), Dr Hazwan Amzar (Sungai Buloh), Dr Ainur Syahidza Bt Abd Manan (NCD Unit, PKD Petaling), Dr Nurhaidi Bt Abdullah (KK Kota Damansara), Dr. Nur Es Naini (KK Kelana Jaya), Dr. Ee Li Min (KK Taman Medan), and Dr Tengku Nor Shafadilah Bt Tg

For peer review only - http://bmjopen.bmj.com/site/about/guidelines.xhtml

Mohd Nordin (KK Paya Jaras) for providing data on breast cancer screening and all participants for completing the surveys.

References

1 Azizah AM, Nor Saleha I, Noor Hashimah A, Asmah Z, Mastulu W. Malaysian National Cancer Registry Report 2007-2011. 2013 doi:MOH/P/KN/01.16(AR).

2 International Agency for Research on Cancer. GLOBOCAN. 2018http://gco.iarc.fr/today/home (accessed 20 Jan 2020).

3 Cancer Research UK. Breast cancer incidence (invasive) statistics. 2017.https://www.cancerresearchuk.org/health-professional/cancer-statistics/statistics-by-cancer-type/breast-cancer/incidence-invasive#heading-Three (accessed 24 Jul 2019).

4 Koo MM, Swann R, Mcphail S, Abel GA, Elliss-brookes L, Rubin GP *et al.* Presenting symptoms of cancer and stage at diagnosis: evidence from a cross-sectional, population-based study. *Lancet Oncol* 2019; **2045**: 1–7.

5 Al-Dubai SAR, Qureshi AM, Saif-Ali R, Ganasegeran K, Alwan MR, Hadi JIS. Awareness and knowledge of breast cancer and mammography among a group of malaysian women in shah alam. *Asian Pacific J Cancer Prev* 2011; **12**: 2531–2538.

6 Abdul Hadi M, Hassali MA, Shafie AA, Awaisu A. Knowledge and perception of breast cancer among women of various ethnic groups in the state of penang: A cross-sectional survey. *Med Princ Pract* 2010; **19**: 61–67.

7 Khan TM, Leong JPY, Ming LC, Khan AH. Association of knowledge and cultural perceptions of Malaysian women with delay in diagnosis and treatment of breast cancer: A systematic review. *Asian Pacific J Cancer Prev* 2015; **16**: 5349–5357.

8 Yu FQ, Murugiah MK, Khan AH, Mehmood T. Meta-synthesis exploring barriers to health seeking behaviour among Malaysian breast cancer patients. *Asian Pac J Cancer Prev* 2015; **16**: 145–152.

9 Mahmud A, Aljunid SM. The uptake of mammogram screening in Malaysia and its associated factors: A systematic review. *Med J Malaysia* 2018; **73**: 202–211.

10 Ministry of Health Malaysia. Clinical Practice Guidelines Management of Breast Cancer Malaysia 2010. Putrajaya, 2010.

11 Talib Z, Amersi F, Harit A, Saleh M. Promoting Breast Cancer Awareness and Clinical Breast

For peer review only - http://bmjopen.bmj.com/site/about/guidelines.xhtml

Examination in the LMIC: Experiences from Tajikistan, Pakistan and Kenya. *Curr Breast Cancer Rep* 2019; **11**: 152–157.

12 Schliemann D, Su TT, Paramasivam D, Treanor C, Dahlui M, Loh SY *et al.* Effectiveness of Mass and Small Media Campaigns to Improve Cancer Awareness and Screening Rates in Asia: A Systematic Review. *JCO Glob Oncol* 2019; **5**: 1–20.

13 Public Health Agency Northern Ireland. Evaluation Report Breast Campaign. Belfast: 2016.

14 Croager EJ, Gray V, Pratt IS, Slevin T, Pettigrew S, D'arcy Holman C *et al.* Find Cancer Early: Evaluation of a community education campaign to increase awareness of cancer signs and symptoms in people in regional Western Australians. *Front Public Heal* 2018; **6**: 4–9.

15 Mazari FAK, Holt S, Azmy IA. The impact of "Be Clear on Cancer" campaign on breast care services provided by a specialist oncoplastic unit – A retrospective case control study. *Int J Surg* 2017; **47**: 54–60.

16 Loh SY, Somasundaram S, Su TT. A Review of Cancer Awareness in Malaysia – What ' s Next ? *Open Access J Cancer Oncol* 2017; **1**: 1–6.

17 Schliemann D, Donnelly M, Dahlui M, Loh SY, Tamin Ibrahim NSB, Somasundaram S *et al.* The 'Be Cancer Alert Campaign': protocol to evaluate a mass media campaign to raise awareness about breast and colorectal cancer in Malaysia. *BMC Cancer* 2018; **18**: 881.

18 Schliemann D, Su TT, Paramasivam D, Ibrahim Tamin NSB, Dahlui M, Loh SY *et al.* The systematic cultural adaptation of a UK public health cancer awareness raising programme for Malaysia: The Be Cancer Alert Campaign. *Transl Behav Med* 2019.

19 Department of Statistics Malaysia. Current Population Estimates, Malaysia, 2017-2018. 2018.https://www.dosm.gov.my/v1/index.php?r=column/cthemeByCat&cat=155&bul_id=c1pqTnFjb29HSnNYNUpiTmNWZHArdz09&menu_id=L0pheU43NWJwRWVSZklWdzQ4TlhUUT09 (accessed 28 Jan2019).

20 Cancer Research UK KCL& UCL. Breast Module of the Cancer Awareness Measure. 2010http://www.cancerresearchuk.org/sites/default/files/health_professional_breast_cam_toolkit_09.02.11.pdf.

21 Public Health Agency. Evaluation Report Primer and Lung Campaigns. 2015.

22 Kirkwood B, Sterne J. *Essential Medical Statistics (2nd Edition)*. Blackwell Publishing, 2003.

23 Ng A, Firouz AMM, Khalidi JR, Muhtar MA, Tumin SA, Man TK *et al.* The state of households 2018: different realities. 2018. http://www.krinstitute.org/assets/contentMS/img/template/editor/FullReport_KRI_SOH_2018.

For peer review only - http://bmjopen.bmj.com/site/about/guidelines.xhtml

pdf (accessed 20 Jan 2020).

24 Public Health England. Evaluation of the Be Clear on Cancer lung cancer awareness campaign regional pilot, East and West Midlands. London: 2015.

25 Schliemann D, Paramasivam D, Dahlui M, Cardwell CR, Somasundaram S, Ibrahim Tamin NSB *et al.* Change in public awareness of colorectal symtpoms following the Be Cancer Alert Campaign in the multi-ethnic population of Malaysia. *BMC Cancer* 2020; **20**: 1-12.

26 Ramanadhan S, Viswanath K. Health and the information nonseeker: a profile. *Health Commun* 2006; **20**: 131–139.

27 Hurley RJ, Riles JM, Sangalang A. Online Cancer News: Trends Regarding Article Types, Specific Cancers, and the Cancer Continuum. *Health Commun* 2014; **29**: 41–50.

28 Glynn RW, Kelly JC, Coffey N, Sweeney KJ, Kerin MJ. The effect of breast cancer awareness month on internet search activity - a comparison with awareness campaigns for lung and prostate cancer. *BMC Cancer* 2011; **11**: 442.

29 Phillips CA, Leahy AB, Li Y, Shapira MM, Bailey L. Relationship Between State-Level Google Online Search Volume and Cancer Incidence in the United States: Retrospective Study. *J Med Internet Res* 2018; **20**.

30 Wallington SF, Oppong B, Iddirisu M, Adams-Campbell LL. Developing a Mass Media Campaign to Promote Mammography Awareness in African American Women in the Nation's Capital. *J Community Health* 2018; **43**: 633–638.

31 Ministry of Health Malaysia. Garis Panduan Program Pengesanan Awal Kanser Payudara Kebangsaan. Malaysia: Bahagian Pembangunan Kesihatan Keluarga, Kementerian Kesihatan Malaysia; 2011. Report No.: MOH/K/ASA/44.11(GU). Putrajaya, 2011.

32 Farid NDN, Aziz NA, Al-Sadat N, Jamaludin M, Dahlui M. Clinical breast examination as the recommended breast cancer screening modality in a rural community in Malaysia; what are the factors that could enhance its uptake? *PLoS One* 2014; **9**: 1–6.

33 Jacobsen GD, Jacobsen KH. Health awareness campaigns and diagnosis rates: Evidence from National Breast Cancer Awareness Month. *J Health Econ* 2011; **30**: 55–61.

34 Li W, Watts J, Tan N. From Screen to Screening: Entertainment and News Television Media Effects on Cancer Screening Behaviors. *J Health Commun* 2019; **24**: 385–394.

35 Karabay O, Hasbahceci M, Kadioglu H. Impact of breast cancer awareness month on detection of breast cancer in a private hospital. *J Int Med Res* 2018; **46**: 619–625.

For peer review only

36 Stead M, Angus K, Langley T, Katikireddi SV, Hinds K, Hilton S *et al.* Mass media to communicate public health messages in six health topic areas: a systematic review and other reviews of the evidence. *Public Heal Res* 2019; **7**: 1–206.

37 Wakefield MA, Loken B, Hornik RC. Use of mass media campaigns to change health behaviour. *Lancet* 2010; **376**: 1261–1271.

38 Hannon P, Lloyd GP, Viswanath K, Smith T, Basen-Engquist K, Vernon SW *et al.* Mass media and marketing communication promoting primary and secondary cancer prevention. *J Health Commun* 2009; **14**: 30–37.

For peer review only

Table legends

Table 1 Socio-demographic characteristics of respondents pre- and post-campaign

Table 2 The relationship between the socio-demographic characteristics of respondents and their recognition of any aspect of the BCAC-BC campaign [a]

Table 3 Be Cancer Alert Campaign – Breast Cancer awareness pre- and post-campaign (n=676)

Table 4 Improvement in overall prompted symptom awareness by socio-demographic characteristics and recognition of BCAC-BC advertisements (binary logistic regression)

Figure legends

Figure 1 Clinical Breast Examinations (CBEs) undertaken in four public clinics (Petaling district) between July 2018 and January 2019

Figure 2 Mammograms undertaken in two public hospitals (Petaling district) between July 2018 and January 2019

Appendix

Appendix Table 1 Information about all campaign activities and media used during BCAC-BC

Appendix Table 2 Change in average prompted knowledge score

Appendix Table 3 Number of CBEs (n=29,000) and mammograms (n=2,051) undertaken by ethnicity and age (July 2018-January 2019)

Appendix Figure 1 Advertisement channels through which participants noticed the BCAC-BC advertisements (unprompted)

Appendix Figure 2 Advertisement channels through which participants noticed the BCAC-BC advertisements (prompted) and views on materials

Appendix Figure 3 Difference in campaign material reach between ethnicities

For peer review only

For peer review only - http://bmjopen.bmj.com/site/about/guidelines.xhtml

Tables

Table 1 Socio-demographic characteristics of respondents pre- and post-campaign

	Pre only *n (%)* *n=316*	Pre and post *n (%)* *n=676*
**Age**		
40-49 years	104 (32.9)	274 (41.0)
50-59 years	102 (32.3)	199 (29.8)
60-69 years	67 (21.2)	137 (20.5)
≥70 years	41 (13.0)	58 (8.7)
**Ethnicity**		
Malay	150 (47.5)	349 (51.6)
Chinese	102 (32.3)	151 (22.3)
Indian	39 (12.3)	120 (17.8)
Others	25 (7.9)	56 (8.3)
**Religion**		
Islam	169 (53.5)	406 (60.1)
Christianity	26 (8.2)	50 (7.4)
Buddhism	81 (25.6)	109 (16.1)
Hinduism	31 (9.8)	100 (14.8)
Others	3 (0.9)	6 (0.8)
**Marital status**		
Married	265 (83.9)	587 (86.8)
Single	51 (16.1)	89 (13.2)
**Education**		
No formal education	45 (14.2)	88 (13.0)
Primary	52 (16.5)	97 (14.4)
Secondary	158 (50.0)	366 (54.2)
Tertiary	59 (18.7)	124 (18.4)
**Family income**		
<RM 4,000	189 (59.8)	457 (71.2)
RM 4,000-10,000	72 (22.8)	140 (21.8)
>RM 10,000	23 (7.3)	45 (7.0)
**BC history** (self only)		
No	307 (97.2)	660 (97.6)
Yes	9 (2.8)	16 (2.4)
**BC screening history** [d]		
No	222 (70.3)	498 (75.5)
Yes	94 (29.7)	175 (25.9)

[a] Participants who are widowed, divorced and who never married

[b] No formal education – includes never schooled/ never completed primary school; primary education – includes completed primary school; secondary education – includes completed form 3/ completed form 5/ certificate/ A-level/ STPM/ HSC; tertiary education – includes diploma/ bachelor degree/ post-graduate degree

[c] Income of all household member combined

[d] BC screening history refers to mammogram in the past 2 years

Missing information: Age (n=3), Religion (n=5), Monthly family income (n=34), BC screening history (n=3)

For peer review only

Table 2 The relationship between the socio-demographic characteristics of respondents and their recognition of any aspect of the BCAC-BC campaign[a]

	n (%)	OR (95% CI) (unadjusted)	*P*	OR (95% CI) (adjusted)[b]	*P*
**Age**					
40-49 years	198/274 (72.3)	*Reference*		*Reference*	
50-59 years	132/199 (66.3)	0.72 (0.48, 1.09)	0.120	0.74 (0.47, 1.16)	0.187
60-69 years	78/137 (56.9)	0.48 (0.31, 0.75)	0.001	0.65 (0.39, 1.10)	0.107
≥70 years	28/58 (48.3)	0.33 (0.18, 0.60)	<0.001	0.47 (0.23, 0.94)	0.032
**Ethnicity**					
Malay	265/349 (75.9)	*Reference*		*Reference*	
Chinese	68/151 (45.0)	0.25 (0.16, 0.37)	<0.001	0.25 (0.15, 0.40)	<0.001
Indian	81/120 (67.5)	0.62 (0.39, 0.99)	0.046	0.69 (0.42, 1.14)	0.145
Others	27/56 (48.2)	0.29 (0.16, 0.52)	<0.001	0.34 (0.18, 0.65)	0.001
**Marital Status**					
Married	380/566 (67.1)	*Reference*		*Reference*	
Single	61/86 (70.9)	1.19 (0.73, 1.96)	0.484	1.26 (0.72, 2.20)	0.422
**Education**					
No formal education	46/88 (52.3)	*Reference*		*Reference*	
Primary	57/97 (58.8)	1.34 (0.74, 2.44)	0.333	1.33 (0.68, 2.57)	0.404
Secondary	255/366 (69.7)	2.14 (1.32, 3.48)	0.002	1.65 (0.93, 2.91)	0.085
Tertiary	82/124 (66.1)	1.99 (1.11, 3.55)	0.021	1.98 (0.93, 4.18)	0.075
**Monthly family income**					
<RM 4,000	311/443 (70.2)	*Reference*		*Reference*	
RM 4,000 -10,000	82/135 (60.7)	0.66 (0.44, 0.98)	0.040	0.73 (0.46, 1.18)	0.202
RM > 10,000	27/43 (62.8)	0.72 (0.37, 1.37)	0.315	0.88 (0.41, 1.86)	0.728
**BC history**					
No	430/660 (65.2)	*Reference*		*Reference*	
Yes	11/16 (68.8)	1.05 (0.36, 3.07)	0.923	1.21 (0.37, 3.98)	0.754
**BC screening history** [c]					
No	337/510 (66.1)	*Reference*		*Reference*	
Yes	103/163 (63.2)	0.93 (0.63, 1.36)	0.705	1.03 (0.67, 1.60)	0.893

n- number of participants 'reached' or who reported that they saw (one or more parts of) the campaign divided by the total number of survey participants

[a] This includes participants who reported that they have been exposed to either the BCAC-BC TV, radio and/or print materials when prompted with the advertisement at follow-up

[b] adjusted for age, ethnicity, marital status, education, monthly family income, BC history and BC screening history

[c] If people replied 'yes' to BC history and BC screening history, they were coded as 'yes' for BC history and 'no' for BC screening history in this model

For peer review only

Table 3 Be Cancer Alert Campaign – Breast Cancer awareness pre- and post-campaign (n=676)

Survey question	**Pre** n (%)	**Post** n (%)	***P*** *(McNemar)*	**Knowledge improvement in BCAC recognisers**[a] n (%)	**Knowledge improvement in BCAC non-recognisers**[b] n (%)	***P*** *(chi-square)*
**Signs and symptoms (unprompted)**						
Change in the position of your nipple	9 (1.3)	7 (1.0)	0.804	7/435 (1.6)	0/208 (0.0)	0.152
Pulling in your nipple	7 (1.0)	16 (2.4)	0.064	11/438 (2.5)	3/207 (1.4)	0.565
Pain in one of your breasts or armpits	128 (18.9)	157 (23.2)	0.041	74/351 (21.1)	27/179 (15.1)	0.122
Puckering or dimpling of your breast skin	4 (0.6)	7 (1.0)	0.549	5/439 (1.1)	2/210 (1.0)	0.999
Discharge or bleeding from your nipple	68 (10.1)	111 (16.4)	<0.001	55/401 (13.7)	19/188 (10.1)	0.272
A lump or thickening in your breast	440 (65.1)	437 (64.6)	0.897	80/143 (55.9)	34/83 (41.0)	0.042
Nipple rash	9 (1.3)	24 (3.6)	0.012	19/437 (4.3)	4/207 (1.9)	0.188
Redness of your breast skin	10 (1.5)	49 (7.2)	<0.001	35/436 (8.0)	11/207 (5.3)	0.279
Lump or thickening under your armpit	23 (3.4)	51 (7.5)	0.001	35/426 (8.2)	10/205 (4.9)	0.174
Changes in the size of your breast or nipple	12 (1.8)	18 (2.7)	0.327	13/435 (3.0)	2/206 (9.7)	0.194
Changes in the shape of your breast or nipple	10 (1.5)	25 (3.7)	0.015	16/434 (3.7)	8/208 (3.8)	1.000
**Signs and symptoms (prompted)**						
Change in the position of your nipple	397 (58.7)	455 (67.3)	<0.001	106/169 (62.7)	42/101 (41.6)	0.001
Pulling in your nipple	391 (57.8)	397 (58.7)	0.745	85/182 (46.7)	32/92 (34.8)	0.079
Pain in one of your breasts or armpits	490 (72.5)	524 (77.5)	0.029	81/110 (44.5)	45/69 (65.2)	0.302
Puckering or dimpling of your breast skin	379 (56.1)	380 (56.2)	0.999	100/187 (53.5)	43/97 (44.3)	0.181
Discharge or bleeding from your nipple	557 (82.4)	560 (82.8)	0.814	55/69 (79.7)	26/47 (55.3)	0.009
A lump or thickening in your breast	598 (88.5)	612 (90.5)	0.211	40/45 (88.9)	18/29 (62.1)	0.014
Nipple rash	345 (51.0)	367 (54.3)	0.227	111/213 (52.1)	46/109 (42.2)	0.117
Redness of your breast skin	371 (54.9)	422 (62.4)	0.003	112/193(58.0)	47/103 (45.6)	0.055
Lump or thickening under your armpit	549 (81.2)	566 (83.7)	0.213	54/68 (79.4)	34/54 (63.0)	0.070
Changes in the size of your breast or nipple	476 (70.4)	497 (73.5)	0.173	78/116 (67.2)	36/78 (46.2)	0.005
Changes in the shape of your breast or nipple	486 (71.9)	511 (75.6)	0.105	80/114 (70.2)	38/69 (55.1)	0.056
				**Improved attitudes in recognisers**[a]	**Improved attitudes in non-recogniser**[b]	
How confident are you that you would notice a BC symptom? *(those very confident/ fairly confident)*	391 (58.9)	439 (68.9)	<0.001	100/157 (63.7)	56/103 (54.4)	0.170
How soon would you go and see a doctor if you noticed a BC sign/symptom? *(those within 2 weeks)*	614 (96.1)	637 (97.1)	0.337	19/20 (95.0)	13/14 (92.9)	0.999

Missing information: confidence pre n=12; confidence post n = 39; delayed help seeking pre n=20; delayed help seeking post n=37

[a] This includes participants who reported that they have been exposed to either the BCAC-BC TV, radio and/or print materials when prompted at follow-up

[b] This includes all participants who reported that they have not seen any of the BCAC-BC TV, radio and/ or print materials when prompted at follow-up

For peer review only - http://bmjopen.bmj.com/site/about/guidelines.xhtml

Table 4 Improvement in overall prompted symptom awareness by socio-demographic characteristics and recognition of BCAC-BC advertisements (binary logistic regression)

	n (%)	OR (95% CI) (unadjusted)	*P*	OR (95% CI) (adjusted)[a]	*P*
**Age**					
40-49 years	122/274 (44.5)	*Reference*		*Reference*	
50-59 years	101/199 (50.8)	1.28 (0.89, 1.85)	0.181	1.36 (0.91, 2.04)	0.130
60-69 years	60/137 (43.8)	0.97 (0.64, 1.47)	0.888	0.97 (0.60, 1.56)	0.890
≥70 years	26/58 (44.8)	1.01 (0.57, 1.79)	0.966	0.99 (0.51, 1.93)	0.982
**Ethnicity**					
Malay	162/349 (46.4)	*Reference*		*Reference*	
Chinese	63/151 (41.7)	0.83 (0.56, 1.22)	0.333	0.82 (0.51, 1.33)	0.420
Indian	58/120 (48.3)	1.08 (0.71, 1.64)	0.717	1.26 (0.75, 2.12)	0.392
Others	30/56 (53.6)	1.33 (0.76, 2.35)	0.321	1.27 (0.67, 2.41)	0.461
**Marital Status**					
Married	270/587 (46.0)	*Reference*		*Reference*	
Single	43/89 (48.3)	1.10 (0.70, 1.72)	0.683	1.17 (0.72, 1.90)	0.532
**Education**					
No formal education	39/88 (44.3)	*Reference*		*Reference*	
Primary	56/97 (57.7)	1.72 (0.96, 3.07)	0.069	1.50 (0.80, 2.81)	0.210
Secondary	156/366 (42.6)	0.93 (0.58, 1.49)	0.773	0.93 (0.54, 1.59)	0.789
Tertiary	62/124 (50.0)	1.26 (0.73, 2.17)	0.415	1.53 (0.77, 3.04)	0.227
**Monthly family income**					
<RM 4000	210/457 (46.0)	*Reference*		*Reference*	
RM 4000-10,000	67/140 (47.9)	1.08 (0.74, 1.58)	0.693	1.15 (0.74, 1.79)	0.528
RM >10,000	19/45 (42.2)	0.86 (0.46, 1.60)	0.632	0.84 (0.42, 1.71)	0.636
**BC history**					
No	307/660 (46.5)	*Reference*		*Reference*	
Yes	6/16 (37.5)	0.69 (0.25, 1.92)	0.477	0.54 (0.18, 1.65)	0.281
**BC screening history**					
No	237/510 (46.5)	*Reference*		*Reference*	
Yes	75/163 (46.0)	0.98 (0.69, 1.40)	0.919	0.83 (0.56, 1.24)	0.358
**TV ad recognition**					
No	155/347 (44.7)	*Reference*		*Reference*	
Yes	154/324 (47.5)	1.12 (0.83, 1.52)	0.457	1.22 (0.85, 1.77)	0.281
**Radio ad recognition**					
No	240/511 (47.0)	*Reference*		*Reference*	
Yes	68/153 (44.4)	0.90 (0.63, 1.30)	0.583	0.79 (0.51, 1.22)	0.289
**Print ad recognition**					
No	221/468 (47.2)	*Reference*		*Reference*	
Yes	88/196 (44.9)	0.91 (0.65, 1.27)	0.584	0.96 (0.66, 1.40)	0.847

[a] Adjusted for age, ethnicity, marital status, education, monthly family income, BC history, BC screening history, TV ad recognition, Radio ad recognition, Print ad recognition

For peer review only

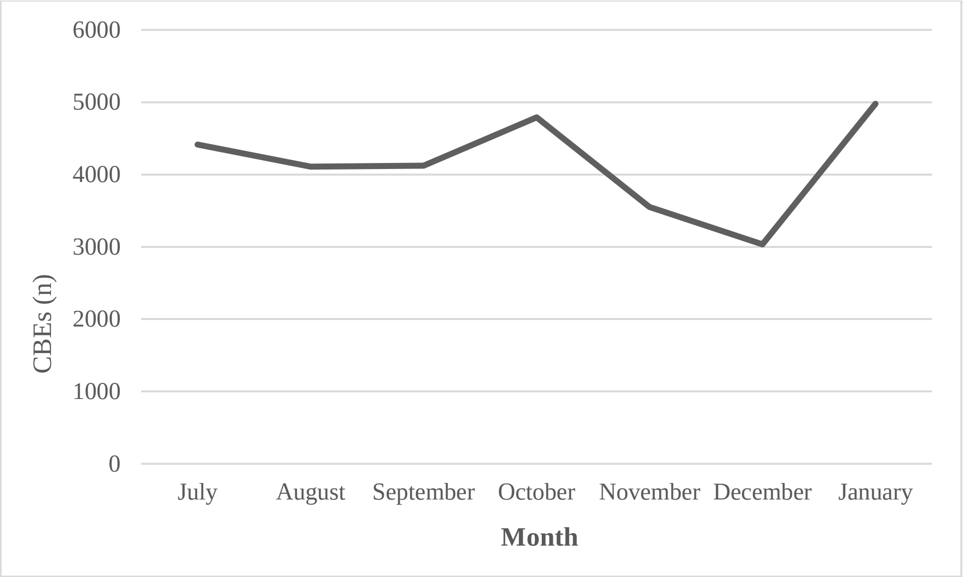

Figure 1 Clinical Breast Examinations (CBEs) undertaken in four public clinics (Petaling district) between July 2018 and January 2019

For peer review only

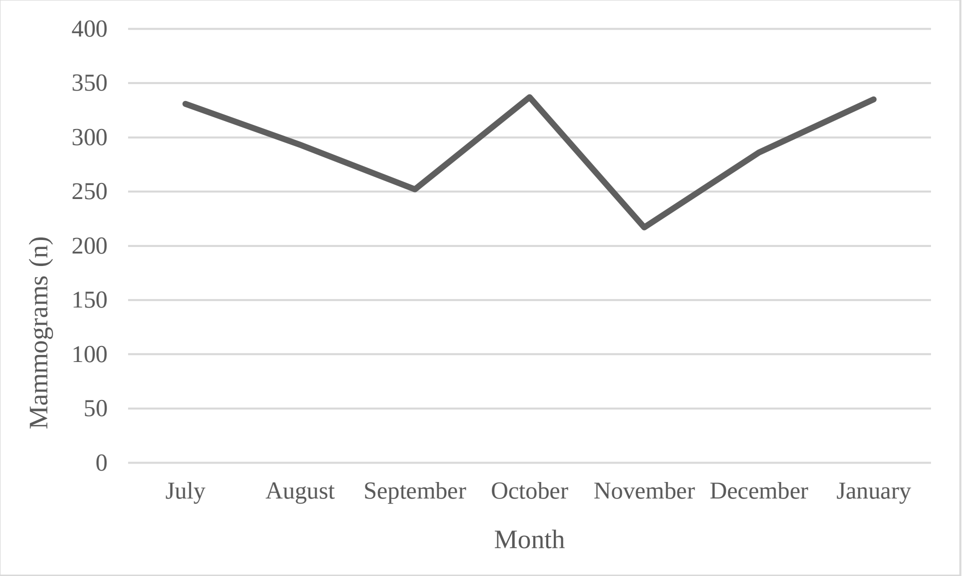

Figure 2 Mammograms undertaken in two public hospitals (Petaling district) between July 2018 and January 2019

For peer review only

Appendix Table 1 Information about all campaign activities and media used during BCAC-BC

**Time period** (2018)	**Media (Language)**	**Content**	**Implementation area**
**TV**			
24th September – 21st October<br>4 weeks<br>4-6 ads/ day	Advertisement, 30 seconds on TV3 (BM), TV9 (BM), 8TV (MAN) and YouTube (BM, MAN, ENG)	Cancer survivor highlighting the signs and symptoms she experienced, promoting early detection can save lives, highlights the importance of family support	Nationwide
**Radio**			
15th – 28th October<br>2 weeks<br>5-7 ads/ day	Advertisement, 30 seconds on I) Thr Raaga (TAM) and II) Lite FM (ENG)	Cancer survivors highlighting BC signs and symptoms, BC can be cured, highlighting that early detection can save lives and to see doctor urgently if symptoms are noticed, 9 out of 10 women survive BC if found and treated early, highlighting website and helpline	I) Peninsular Malaysia<br>II) Central Malaysia (including Petaling Jaya)
**Print materials**			
24th September – 28th October<br>5 weeks	Billboards (x7) (BM)	Displayed three BC survivors from each main ethnicity as well as Amber Chia (Malaysian supermodel) and the headline 'Don't be shy to check your breasts' and the slogan '9 out of 10 women can survive BC if found and treated early', information about the website and NCSM helpline	Petaling Jaya [a]
	Street buntings (x400) (BM)	*See billboards*	Petaling Jaya [a]
	Posters (BM and ENG)	*See billboards* + signs and symptoms of BC, see a doctor urgently if symptoms are noticed, early detection can save lives, BC can be cured	Clinics (A2 size x53) in Petaling Jaya [a]<br>Tropicana Shopping Mall (A1 size x 8)
	Roll-up banners	*See billboards*	Supermarkets (x5) in Petaling Jaya [a]
	Brochures (BM, ENG, MAN and TAM)	*See billboards* + signs and symptoms of BC, Breast-self-examination guide, early detection can save lives, BC survivor story	Clinics (x53) in Petaling Jaya [a]

For peer review only - http://bmjopen.bmj.com/site/about/guidelines.xhtml

8th October (x2), 14th October (x2), 28th October (x1)	5x Newspaper articles (BM and ENG), i.e. MY Metro Online, Harian Metro, The Star Online, Sunday Star, Berita Harian	*Key messages same as posters*	Respective areas of coverage
**Social media**			
24th September – 28th October<br>5 weeks	32 posts, including 11 boosted posts (BM, ENG)<br>2 x posts from Amber Chia (Malaysian supermodel)	What is BC, BCAC-BC campaign launch, signs and symptoms, breast self-examination, risk factors, prevalence of BC in Malaysia, importance of male support, prevention, campaign video, cancer survivor stories (x3), call to action, screening and diagnosis, early detection saves lives, quiz	Nationwide
**Website**			
	Link: www.becanceralert.com (BM and ENG)	Information about cancer in general, signs and symptoms, risk factors and early detection as well as access to all BCAC-BC materials, cancer survivor stories	Nationwide
**Launch event**			
27th September	1 hour launch event (ENG)	Stakeholders and media were invited to officially launch the BCAC-BC	University of Malaya, Kuala Lumpur

[a] Petaling Jaya was the study area for BCAC-BC

Ads – advertisement, BCAC – Be Cancer Alert Campaign, BM – Bahasa Melayu, BC – breast cancer, ENG- English, MAN – Mandarin, NCSM – National Cancer Society Malaysia, TAM- Tamil

For peer review only

Appendix Table 2 Change in average prompted knowledge score

	Pre Mean (SD)	Post Mean (SD)	Change Mean (SD)	P-value *(paired sample t-test)*	P-value *(independent sample t-test, equal variances assumed)*
All participants	7.45 (3.05)	7.84 (2.86)	0.39 (3.53)	0.005	-
BC history (n=16)	8.00 (2.99)	8.06 (2.91)	0.06 (3.26)	0.940	*Comparing BC history to no history* 0.711
No BC history (n=660)	7.44 (3.06)	7.83 (2.86)	0.39 (3.54)	0.004	
BC screening history (n=175)	7.65 (3.20)	8.31 (2.85)	0.66 (3.39)	0.011	*Comparing BC screening history to no screening history* 0.251
No BC screening history (n=498)	7.38 (3.00)	7.69 (2.83)	0.31 (3.54)	0.052	
BCAC recognisers (n=441)	7.68 (2.89)	8.26 (2.46)	0.59 (3.29)	<0.001	*Comparing BCAC recognisers to non-recognisers (equal variances not assumed)* 0.0501
BCAC non-recognisers (n=211)	6.98 (3.29)	6.96 (3.32)	-0.24 (3.88)	0.929	

Appendix Table 3 Number of CBEs (n=29,000) and mammograms (n=2,051) undertaken by ethnicity and age (July 2018-January 2019)

	**CBE** *n* (%)	**Mammogram**[a] *n* (%)
**Ethnicity**		
Malay	24 532 (84.6)	1152 (56.2)
Chinese	2 434 (8.4)	551 (26.9)
Indians	1 516 (5.2)	331 (16.1)
Others	518 (1.8)	17 (0.8)
**Age group**		
20-29	16 687 (57.5)	1 (0.1)
30-39	10 722 (37.0)	14 (1.6)
40-49	1 283 (4.4)	332 (37.0)
50-59	265 (0.9)	333 (37.1)
>60	43 (0.1)	-
60-69	-	172 (19.2)
>70	-	46 (5.1)

CBE – clinical breast examination

[a] Mammogram data on age is from hospital Sg Buloh only (n=898)

For peer review only

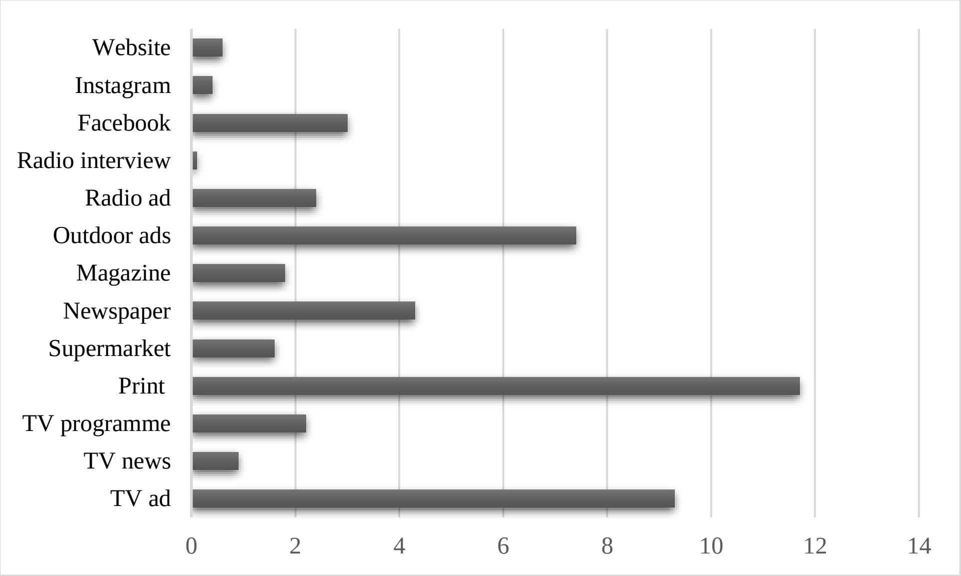

Appendix Figure 1 Advertisement channels through which participants noticed the BCAC-BC advertisements (unprompted)

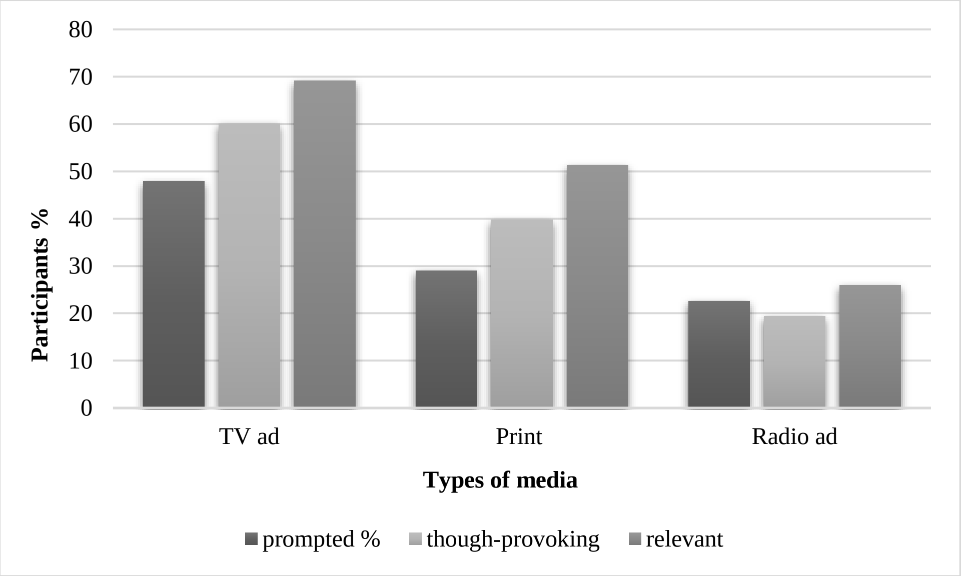

Appendix Figure 2 Advertisement channels through which participants noticed the BCAC-BC advertisements (prompted) and views on materials

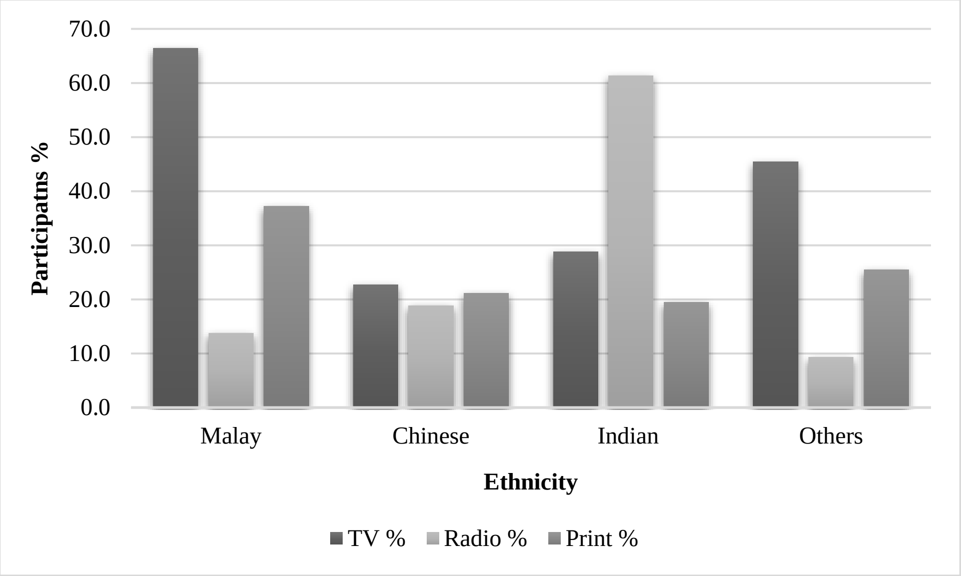

Appendix Figure 3 Difference in campaign material reach between ethnicities

For peer review only

STROBE 2007 (v4) checklist of items to be included in reports of observational studies in epidemiology*
Checklist for cohort, case-control, and cross-sectional studies (combined)

Section/Topic	Item #	Recommendation	Reported on page #
**Title and abstract**	1	(*a*) Indicate the study's design with a commonly used term in the title or the abstract	2
		(*b*) Provide in the abstract an informative and balanced summary of what was done and what was found	2
**Introduction**			
Background/rationale	2	Explain the scientific background and rationale for the investigation being reported	4
Objectives	3	State specific objectives, including any pre-specified hypotheses	4
**Methods**			
Study design	4	Present key elements of study design early in the paper	4,5
Setting	5	Describe the setting, locations, and relevant dates, including periods of recruitment, exposure, follow-up, and data collection	5
Participants	6	(*a*) *Cohort study*—Give the eligibility criteria, and the sources and methods of selection of participants. Describe methods of follow-up<br>*Case-control study*—Give the eligibility criteria, and the sources and methods of case ascertainment and control selection. Give the rationale for the choice of cases and controls<br>*Cross-sectional study*—Give the eligibility criteria, and the sources and methods of selection of participants	4,5
		(*b*) *Cohort study*—For matched studies, give matching criteria and number of exposed and unexposed<br>*Case-control study*—For matched studies, give matching criteria and the number of controls per case	
Variables	7	Clearly define all outcomes, exposures, predictors, potential confounders, and effect modifiers. Give diagnostic criteria, if applicable	5
Data sources/ measurement	8*	For each variable of interest, give sources of data and details of methods of assessment (measurement). Describe comparability of assessment methods if there is more than one group	5
Bias	9	Describe any efforts to address potential sources of bias	5,6
Study size	10	Explain how the study size was arrived at	6
Quantitative variables	11	Explain how quantitative variables were handled in the analyses. If applicable, describe which groupings were chosen and why	6
Statistical methods	12	(*a*) Describe all statistical methods, including those used to control for confounding	6
		(*b*) Describe any methods used to examine subgroups and interactions	6
		(*c*) Explain how missing data were addressed	6
		(*d*) *Cohort study*—If applicable, explain how loss to follow-up was addressed<br>*Case-control study*—If applicable, explain how matching of cases and controls was addressed	

For peer review only

		Cross-sectional study—If applicable, describe analytical methods taking account of sampling strategy	
		(*e*) Describe any sensitivity analyses	
**Results**			
Participants	13*	(a) Report numbers of individuals at each stage of study—eg numbers potentially eligible, examined for eligibility, confirmed eligible, included in the study, completing follow-up, and analysed	7
		(b) Give reasons for non-participation at each stage	7
		(c) Consider use of a flow diagram	
Descriptive data	14*	(a) Give characteristics of study participants (eg demographic, clinical, social) and information on exposures and potential confounders	7
		(b) Indicate number of participants with missing data for each variable of interest	19,20
		(c) *Cohort study*—Summarise follow-up time (eg, average and total amount)	
Outcome data	15*	*Cohort study*—Report numbers of outcome events or summary measures over time	
		Case-control study—Report numbers in each exposure category, or summary measures of exposure	
		Cross-sectional study—Report numbers of outcome events or summary measures	7,8
Main results	16	(*a*) Give unadjusted estimates and, if applicable, confounder-adjusted estimates and their precision (eg, 95% confidence interval). Make clear which confounders were adjusted for and why they were included	8-10, 20,22
		(*b*) Report category boundaries when continuous variables were categorized	19
		(*c*) If relevant, consider translating estimates of relative risk into absolute risk for a meaningful time period	
Other analyses	17	Report other analyses done—eg analyses of subgroups and interactions, and sensitivity analyses	7-10, 20-23
**Discussion**			
Key results	18	Summarise key results with reference to study objectives	9,10
Limitations	19	Discuss limitations of the study, taking into account sources of potential bias or imprecision. Discuss both direction and magnitude of any potential bias	10,11
Interpretation	20	Give a cautious overall interpretation of results considering objectives, limitations, multiplicity of analyses, results from similar studies, and other relevant evidence	9-11
Generalisability	21	Discuss the generalisability (external validity) of the study results	11
**Other information**			
Funding	22	Give the source of funding and the role of the funders for the present study and, if applicable, for the original study on which the present article is based	12

*Give information separately for cases and controls in case-control studies and, if applicable, for exposed and unexposed groups in cohort and cross-sectional studies.
Note: An Explanation and Elaboration article discusses each checklist item and gives methodological background and published examples of transparent reporting. The STROBE checklist is best used in conjunction with this article (freely available on the Web sites of PLoS Medicine at http://www.plosmedicine.org/, Annals of Internal Medicine at http://www.annals.org/, and Epidemiology at http://www.epidem.com/). Information on the STROBE Initiative is available at www.strobe-statement.org.

For peer review only
